# Asian summer monsoon orbital variability directly paced by CO2 and precession, not eccentricity

**DOI:** 10.1038/s41467-026-76856-y

**Published:** 2026-08-22

**Authors:** Jeanne Millot-Weil, Paul J. Valdes, Alexander Farnsworth

**Affiliations:** 1 School of Geographical Sciences, University of Bristol, Bristol, UK; 2 State Key Laboratory of Tibetan Plateau Earth System, Environment and Resources, Institute of Tibetan Plateau Research, Chinese Academy of Sciences, Beijing, 100101 China

**Keywords:** Palaeoclimate, Climate and Earth system modelling

## Abstract

The pacing and intensity of the Asian summer monsoon have long been debated. Geological records indicate either a dominant precession or eccentricity variability, while modelling studies have largely emphasised monsoon intensity rather than its temporal pacing. Here, we investigate both the temporal variability and changes in intensity of the Asian summer monsoon using a unique set of four series of full-complexity climate model equilibrium simulations spanning the last 800 kyr at 4-kyr resolution. The simulations have differing insolation, greenhouse gases, and ice sheet forcing. Here, we show that variations in greenhouse gases exert the dominant control on the 100-kyr variability via the thermal forcing of the Asian summer monsoon circulation, while precession directly impacts the 20-kyr variability. In contrast, the direct contributions of eccentricity forcing and ice sheet variability are smaller. Spatial heterogeneity in monsoon dynamics reveals different sensitivities to imposed forcings, which may help reconcile conflicting proxy records.

## Abstract (web summary)

Asian summer monsoon variability is investigated using climate model simulations covering the last 800 kyr. Greenhouse gases drive 100-kyr variability via its thermal forcing, while precession governs 20-kyr variability.

## Introduction

Asian monsoon variability (intensity and seasonality) over the last 800,000 years has been extensively investigated. Multiproxy geological records reveal variability on orbital time-scales (eccentricity  ≈ 100- or 400-kyr, obliquity  ≈ 40-kyr and precession  ≈ 20-kyr cycle)^1,2^. However, the large number of available records show that the dominant drivers of these orbital-scale variations remain controversial. This debate mainly reflects the diversity of modes of variability identified in different geological archives, that can often contradict one another. For example, a dominant  ≈ 100-kyr signal has been identified in continental loess records^3,4^, whereas oxygen isotope records from cave speleothems reveal a dominant  ≈ 20-kyr precession cycle^5^. This is consistent with other loess archives showing strong  ≈ 20-kyr variability^6^. Furthermore, mixed pacing has also been identified in benthic oxygen isotopes^7^, while stacked monsoon proxy records exhibit a combination of eccentricity, precession, and obliquity pacing^8^. This disagreement between records has more recently been coined the “Chinese 100-Kyr problem"^9^. Moreover, separating the Asian monsoon into its regional component monsoons, such as the South Asian Summer Monsoon (SASM), further complicates this picture. For example, stable oxygen and carbon isotope records from sediment cores in the Bay of Bengal exhibit strong obliquity and precession fluctuations^10^.

Variations in Earths orbit modulate incoming solar radiation (insolation) received at the top of the atmosphere leading to changes in the climate system. Power spectral analysis of this incoming radiation shows that at most latitudes and times of the year, precession is the dominant forcing with relatively weak power at eccentricity periods. Obliquity can be important at high latitudes. However, these variations in insolation lead to variations in ice sheet height and extent, and in atmospheric greenhouse gas concentrations^11^ and these are tightly coupled through multiple feedback processeses. Together, changes in orbital insolation, ice-sheet and greenhouse gas variability constitute three key forcings that drive changes in local, regional and global climate patterns, including the Asian monsoon.

The traditional description of the monsoon system (large-scale sea breeze driven by a strong temperature gradient between the land and ocean^12^), has been supported by various numerical modelling experiments which have underscored the role of various forcings. In particular, the impact of insolation forcing on the intensity of the land-sea thermal contrast has been highlighted^13–17^. However, this traditional concept of the monsoon has been challenged. Rather than a simple large-scale sea-breeze circulation, the monsoon system has been increasingly interpreted as a seasonal shift of the convergence zone (eddy-driven Intertropical Convergence Zone (ITCZ), or angular momentum conserving monsoon-like convergence zone to describe extra tropical monsoons) and associated seasonal displacement of moist static energy maxima^18^^,^^19^. Using both traditional and revised definitions, modelling studies have emphasised the importance of greenhouse gases in increasing monsoon precipitation^20^ and weakening the atmospheric circulation associated with the Asian monsoon system by affecting sea surface temperature (SST)^21^. Ice sheets can also thermodynamically impact Asian monsoon circulation by altering surface albedo in the Northern Hemisphere, leading to temperature changes over the land and thus altering the land-ocean thermal constrast that drives monsoon flow. Ice sheet growth can also modify hemispheric pressure gradients which can impact monsoon circulation^14^. In addition, ice sheet topography can perturb the westerly jet, affecting dry air advection and hence Asian monsoon intensity^22–24^.

Modelling studies have the potential for disentangling the relative influence of each of these drivers on Asian monsoon intensity for particular time periods, but have been more limited in their ability to evaluate the role of different forcings in the orbital pacing of the monsoon. This is because climate models can generally not be run for multiple orbital time periods. Recently, growth in computing power has enabled some model analysis of the periodicity. For instance, Lu et al.^25^ noted synchronous variations between CO2 concentration and East Asian summer monsoon precipitation during the last 21,000 years that have been supported by TraCE-21000 simulations. On the other hand, simulations with the intermediate complexity model LOVE-CLIM included different ice sheet configurations at perihelion and aphelion and argued that ice sheets play a primary role in regulating East Asian precipitation intensity^26^. But neither of these studies directly examined the pacing of the Asian monsoon. Recently Lyu et al.^24^, considered not only the East Asian monsoon intensity but also its pace over the last 800-kyr and showed a direct impact of ice sheet and precession cycle. However, this study used a Gaussian emulator, not direct simulations, based on previous HadCM3 simulations. For the SASM monsoon, Prell and Kutzbach^13^ argued for a direct role of solar (orbital) forcing itself in modulating precipitation intensity based on experiments which applied different forcings in climate model simulations, but these only extended over the last 21,000 years and so could not address longer time scales. More recently, Araya-Melo et al.^27^ used an emulator-based approach to show that precession and ice sheets are the primary drivers of monsoon precipitation intensity.

Thus, given the diversity of proposed drivers and pronounced regional heterogeneity recorded by geological data^1,9,28,29^, there is still much uncertainty in the driving mechanisms of Asian monsoon variations at orbital time scales. While proxy records constrain both the pacing and intensity of the Asian monsoon (and component monsoons), most modelling studies have focused on the impact of individual forcings on monsoon intensity at specific orbital configurations, limiting direct and consistent model-proxy comparisons.

Here, we focus on the summer monsoon and exploit the recently updated ocean-atmosphere general circulation model HadCM3B^30,31^ to simulate climate dynamics over the last 800 kyr at high temporal resolution ( ≈ every 4 kyr; 219 snapshot simulations) using four distinct forcing configurations. The first, called All_forcings, includes time-varying insolation, ice sheets and greenhouse gases, and provide a timeseries that can be directly compared to the geologic record. The second, called Orb_Ghg, prescribes a constant pre-industrial ice sheet throughout the study period, while insolation and greenhouse gases concentration are varying. The third, called Orb_Ice, holds greenhouse gases fixed at pre-industrial concentrations over the full 800-kyr period, but allows ice sheets and insolation to vary. Finally, the fourth set (Table 1), Orb, only has insolation varying, with ice sheet and greenhouse gases constant at pre-industrial levels throughout the 800-kyr. Together, these experiments enable the individual and combined influences of orbital forcing, ice-sheet variability, and atmospheric CO2 concentration to be isolated and quantified for Asian monsoon variability.

**Table 1:** Summary of simulation sets characteristics

Schemes	Ice sheet	Greenhouse gases	Orbital parameters
All_forcings	Changing	Changing	Changing
Orb_Ghg	Constant	Changing	Changing
Orb_Ice	Changing	Constant	Changing
Orb_Only	Constant	Constant	Changing

## Results and discussion

### Assessment of the Quaternary Asian summer monsoon in HadCM3

HadCM3 has been shown to reproduce key features of the modern Asian monsoon system compared to observations, outperforming many other CMIP5 models^32^. However, it is also necessary to evaluate how the model represents the Asian monsoon, specifically the summer monsoon, across the full range of climate states over the last 800 kyr. Traditionally, the summer monsoon in Asia has been described as a large-scale sea-breeze circulation, in which strong land-sea thermal contrast drives moist onshore flow over the continent, leading to heavy precipitation over land^12^. More recent studies tend to describe the different monsoon systems as components of a global system characterised by the position of the summer convergence zone that results in heavy precipitation^18^^,^^19^. This global approach, however, does not exclude the impact of regional characterisitcs that can further modulate monsoon behaviour resulting from different land-sea distributions, topographies and ocean circulation patterns^33^. Notably, Geen et al.^19^ distinguished two regimes of meridional overturning circulation among regional monsoons: an ITCZ regime characterised by a near-equatorial convergence zone (approximately 10°N), and a monsoon regime (or extratropical monsoon), in which the convergence zone is displaced much farther northward (approximately 20–30°N).

To assess which of these two regimes the Asian summer monsoon belongs to, we compare changes in the Hadley circulation over the Indo-Asian (Supplementary Fig.4), East Asian (Supplementary Fig.5), and broader Asian (Supplementary Fig.6) domains between periods of maximum and minimum summer precipitation. Minimum precipitation is defined as values below the first quantile of the simulations ensemble, and maximum summer precipitation as values above the third quantile. With exception of the weakest summer-monsoon cases in the Indo-Asian sector, the convergence zone typically lies North of 10^*o*^ N, as indicated by both the zero crossing of the streamfunction and the latitude of maximum subcloud moist static energy (MSE). Over East Asia, the convergence zone is located much farther poleward (North of 30 ^*o*^N) than over the Indian sector (near or South of 20 ^*o*^N), consistent with the development and progression of the Meiyu-Baiu front, a characteristic frontal zone that interacts with the westerly jet and the Tibetan Plateau^34,35^, and which brings monsoon precipitation to higher latitudes. These analyses are consistent with the precipitation distribution shown in Supplementary Fig.2, which extends precipitation beyond 20°N in most simulations, except over the Indian sector for simulations showing the lowest summer precipitation. Hence, overall in our simulations the Asian summer monsoon generally falls within the monsoon regime identified by Geen et al.^19^, except for the weakest Indo-Asian cases.

**Fig. 1:**
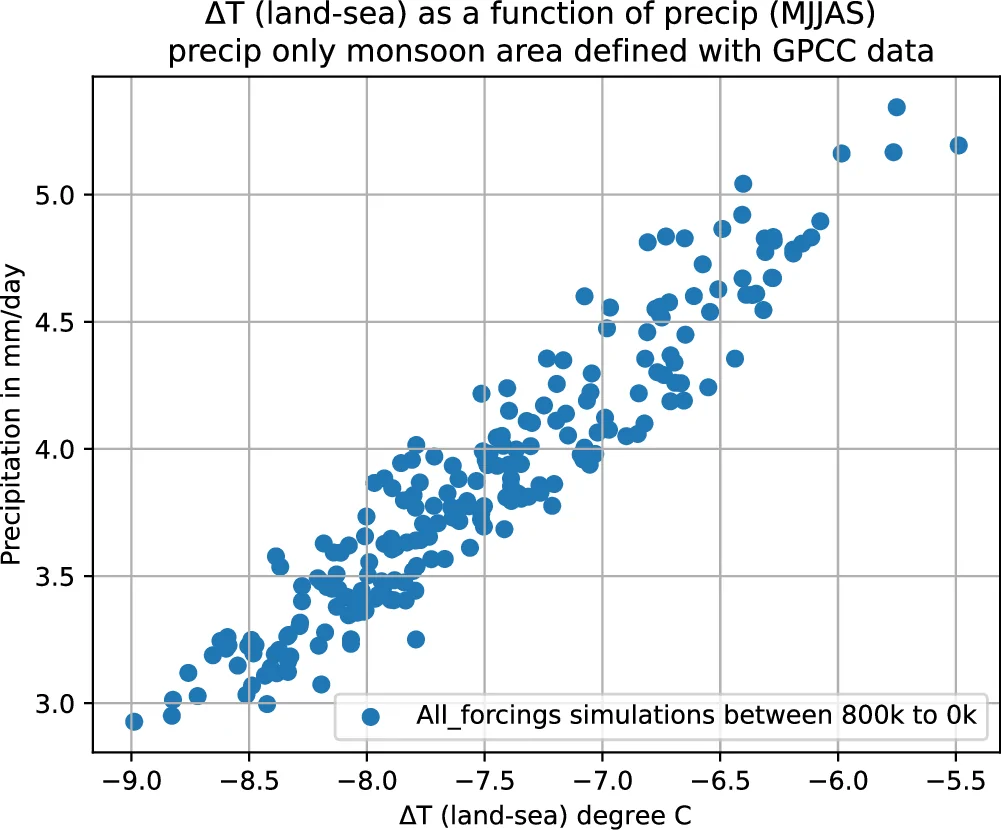
Asian summer monsoon precipitation-temperature relationship. Asian Summer monsoon precipitation (mm/day) (cf blue shaded area Fig. 2) versus the Northern Hemisphere summer (May-September; MJJAS) land-sea temperature (°C) contrast, calculated over the region presented in Fig. 2 (lat : 0/50 N, lon : 60/130 E). Each point represents one of the 219 All_forcings snapshot simulations.

**Fig. 2:**
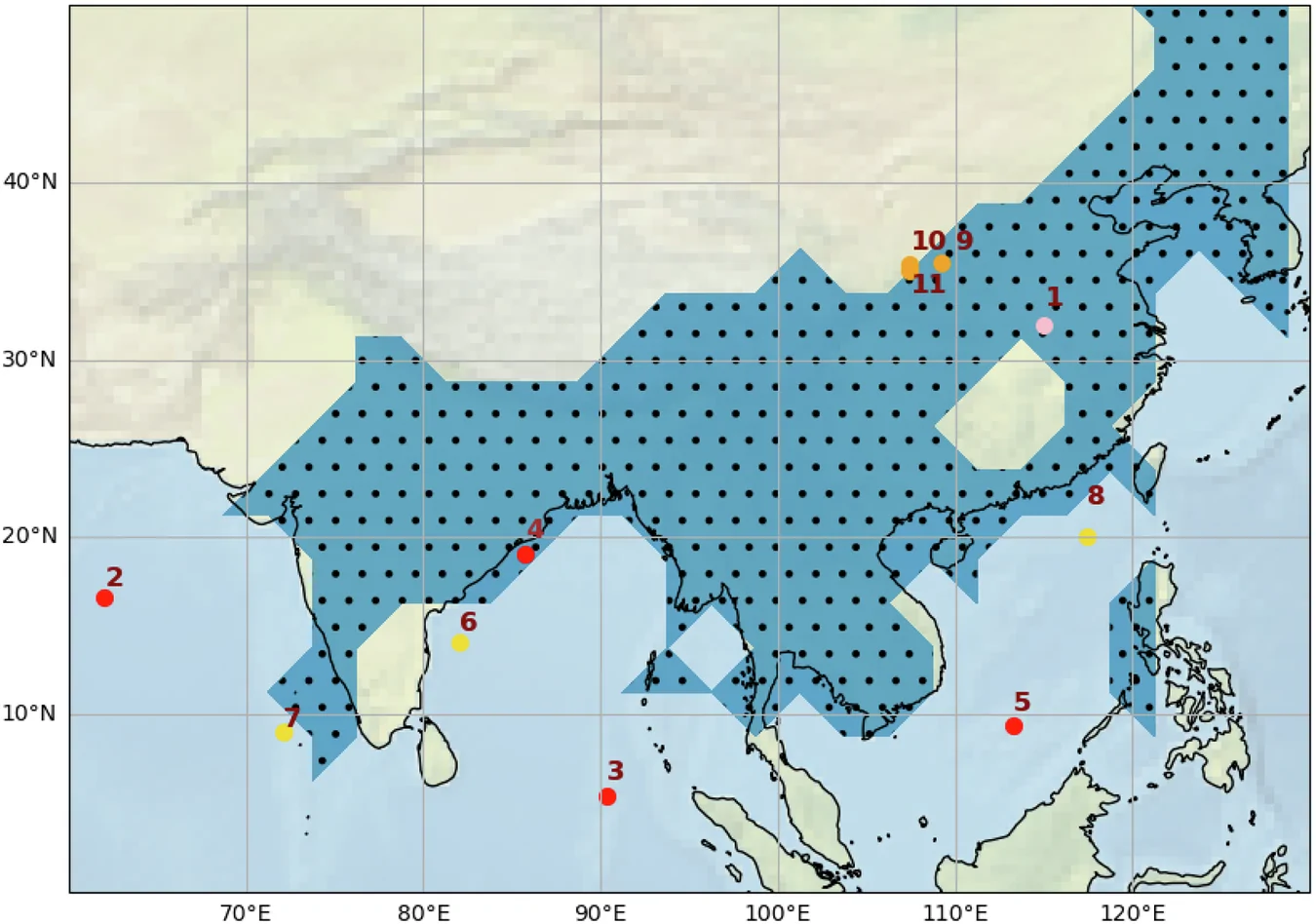
Study region and location of proxy records. (1) Speleothem *δ*18O records from Hulu/Sanbao caves^56^, (2) Benthic *δ*18O (*δ*18O_*b*_) record from ODP 722^72^, (3) *δ*18O_*b*_ record from ODP 758^73^, (4) Sea water *δ*18O (*δ*18O_*sw*_) derived from planktonic *δ*18O record from ODP U1446^74^, (5) *δ*18O_*b*_ record from ODP U1443^7^, (6) Foraminiferal Mg/Ca record from core SK218/1 (SST proxy)^76^, (7) Foraminiferal Mg/Ca record from SK221-05 gravity core (SST proxy)^75^, (8) foraminiferal Mg/Ca record from ODP 1144 (Sea Surface Temperature (SST) proxy)^45^, (9) glycerol dialkyl glycerol tetraether-derived temperature from Lingtai loess-paleosol sequence^42^, (10) and (11) branched glycerol dialkyl glycerol tetraether-derived temperature from Luochan and Xifeng loess paleosol sequence respectively^41^. The blue shaded area indicates the summer (MJJAS) Asian monsoon precipitation domain^70^ derived from GPCC observations ^69^ and regridded to HadCM3 resolution. Map created using cartopy^77^.

The monsoon regime is highly sensitive to thermal forcing, reflecting the energy budget required for monsoon-like development^19^^,^^36^. Therefore, we use the “All_forcings" simulation series to evaluate the thermal dependence of the monsoon precipitation over the last 800 kyr. Fig. 1 shows the relationship between summer (May to September - MJJAS) monsoon precipitation (Fig. 2 blue shaded area) and the land-sea summer temperature contrast over the domain Fig. 2. Each point in the scatter plot represents one simulation among the 219. Modelled precipitation and land-sea temperature contrast are strongly correlated (Pearson test given a high correlation coefficient of 0.93 (associated *p*_value = 3.1e-97 with a 95% confidence level)). In addition, Asian summer monsoon precipitation (across East Asian, South Asian, and combined domains) in the model is highly correlated with summer latent heat flux and spring sensible heat flux over the Tibetan Plateau (Supplementary Fig.7), supporting the role of latent heating in monsoon intensification^37^ and the role of spring uplifted sensible heating in monsoon initiation^38^^,^^39^. Consistent with the above results, summer land-sea temperature contrast strongly correlates with summer latent and spring sensible heat fluxes over the Tibetan Plateau (Supplementary Fig.7). As such, the summer land-sea temperature contrast emerges as an important driver of the initiation and intensification of the Asian summer monsoon in our simulations, providing sufficient energy to extend the circulation farther poleward of the ITCZ location. Since we found in the first order similar relationships between summer precipitation in East Asian, South Asian and combined domains with the driving heat fluxes of the monsoon (Supplementary Fig.7), the following results will focus on the broad domain shown in Fig. 2.

**Fig. 3:**
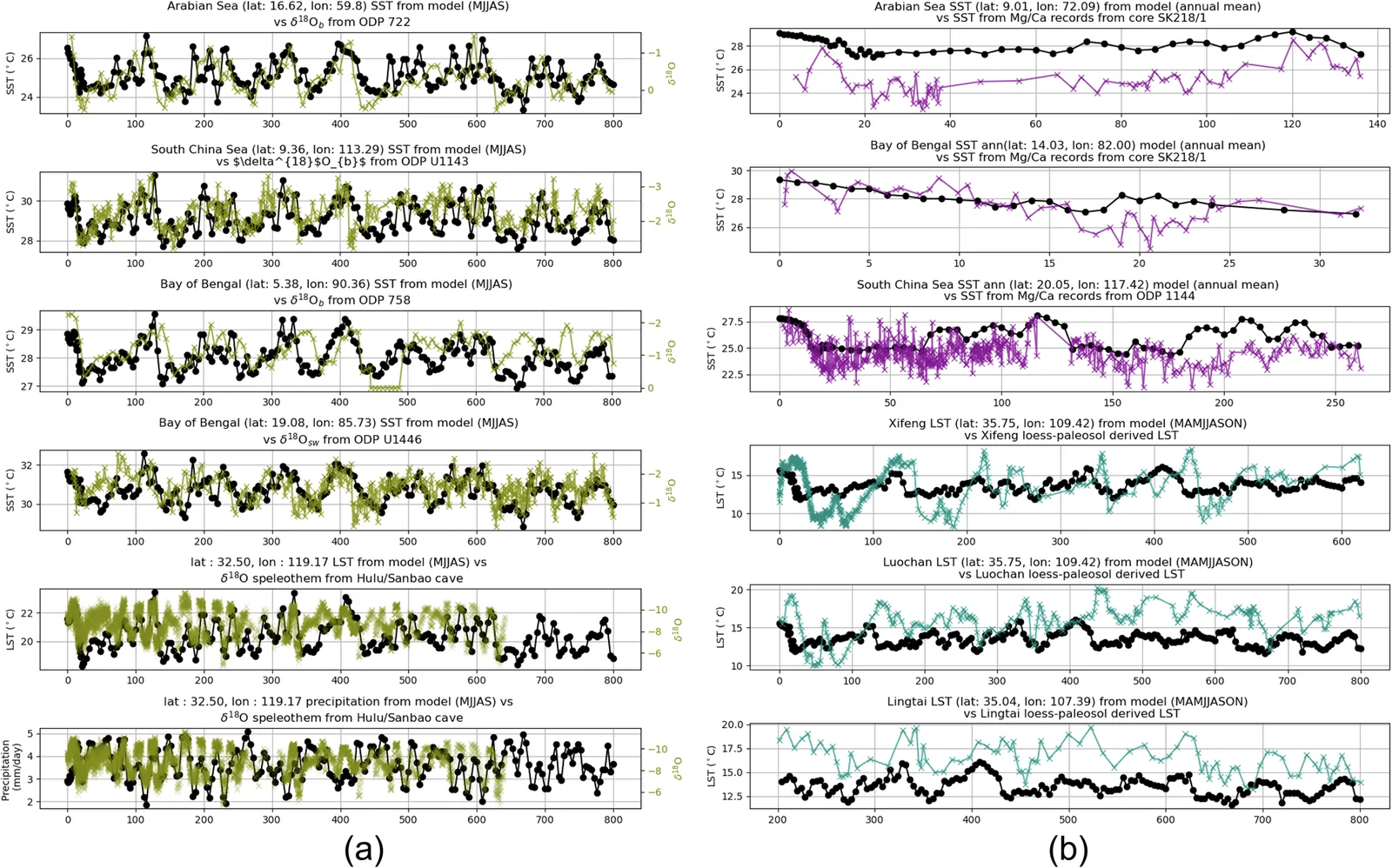
Model-proxy comparison. Timeseries from the All_Forcings simulation set (black) and geological records. In (**a**), *δ*^18^O records (green) are compared with simulated summer (MJJAS) mean SST from the model for marine sites with simulated MJJAS precipitation (mm/day) and land surface temperature (LST; (^∘^C)) for speleothem sites (**b**) Mg/Ca records (purple) are compared with simulated annual mean SSTs, and loess-paleosol records (blue) are compared with simulated March-November (MAMJJASON) mean LST. (b)). Records location and references are seen in Fig. 2.

To verify whether this model behaviour across the last 800 kyr is consistent with proxy-observations, we make a comprehensive comparison against available *δ*^18^O, Mg/Ca and loess records. *δ*^18^O data from marine cores and speleothem can capture orbital variations at high spatiotemporal resolution through the Quaternary^40^. Accordingly, we compare simulated summer (MJJAS) surface temperature with marine *δ*^18^O records to evaluate whether the model accurately reproduces the orbital-scale variability. Specifically, we average basin-mean model sea surface temperature (SST) for the Arabian sea (lat: 0/25N, lon: 45/78E), Bay of Bengal (lat: 0/25N, lon: 78/100E) and South China Sea (lat: 0/25N, lon: 105/130E), and compare the resulting SST variations with marine *δ*^18^O proxies which are typically interpreted as representing temperature variations (see Fig. 3, records 2), 3), 4) and 5)). In addition, we compare land surface temperature (LST) and land precipitation variations over the broader region (Fig. 2, record 1)) with speleothem records from Hulu/Sanbao cave, which have widely been used as indicators of regional hydroclimate variability^1^). Furthermore, we compare temperature values from the model against additional, direct temperature proxies. For SST, we use three temperature reconstructions from Mg/Ca (see Fig. 3, records 7), 6) and 8)) and compare them against simulated annual SST. For LST, we use temperature reconstruction derived from loess records ((see Fig. 3, records 9), 10) and 11)) and compare with LST averages between March and November (bacterial growth season^41^^,^^42^) sampled at the records location. Fig. 3a shows the comparison between model timeseries and *δ*^18^O, Fig. 3b shows the comparison between the model timeseries and temperature proxies.

Pearson’s correlation tests (95% confidence) between simulated SST variations and marine *δ*^18^O records (Table 2) indicate significant agreement between geological and model data. Correlations for the Bay of Bengal are weaker than for the other basins, potentially reflecting regional influences that can decouple *δ*^18^O from basin average SST, including surface runoff ^43^ and boundary current variability. Additionally, we perform the exact same analysis (not shown) but, instead of comparing individual proxy records to regional model averages, we use the closest grid-box to the marine core or speleothem location. We obtain comparable results for the temperature timeseries, whereas the correlation between the Hulu/Sanbao cave speleothem and precipitation yield weaker coefficient (0.36, *p*_value : 5.1 e^−8^) when we compare with local grid box outputs rather than with a regional average. This result suggests that the speleothem may give information about regional precipitation or moisture rather than local^44^. The consistency between the model response and the *δ*^18^O record over the last 800 kyr indicates that the model accurately reproduces the pacing of orbital-scale variability over the region. With respect to the temperature values, Fig. 3b reveals good agreement between the model and proxy timeseries. The average bias between model and proxy SSTs is +1.73 °C, and the average bias between model and proxy LST is −1.71 °C (outside the uncertainty range of the loess reconstruction  ± 0.8 °C given by ref. ^41^, and of the Mg/Ca reconstruction  ± 0.5 °C from ref. ^45^). Pearson’s correlation Table 2 between the geological data and model show significant correlation between proxy and model temperatures, although higher coefficients are found for the SST rather than for LST. This may reflect greater chronologic uncertainty typically associated with loess records than compared with Mg/Ca reconstructions, and/or limitations in the model representation of complex land-sea interactions. Correlations were not significant according to the *p*_value between model SST and Mg/Ca records over the Bay of Bengal, neither between model LST and Luochuan loess-paleosol sequence.

**Table 2:** Model-proxy data temperature correlation

		All forcings
Geological Records	Correlation Coefficient	*p*-value
Sea Surface Temperature MJJAS (averaged over each basin)		
Arabian Sea - *δ*18O_*b*_ - ODP 722^72^	0.65	1.8 e-18
South China Sea - *δ*18O_*b*_ - ODP U1443^7^	0.52	2.2 e-16
Bay of Bengal - *δ*18O_*b*_ - ODP 758^73^	0.30	3.6 e-4
Bay of Bengal - *δ*18O_*sw*_ - ODP U1446^74^	0.39	1.5 e-9
Sea Surface Temperature ANN (local grid point)		
Arabian Sea - Mg/Ca - SK221-05^75^	0.55	2.04 e-5
Bay of Bengal - Mg/Ca - SK218/1^76^	-	not significant
South China Sea - Mg/Ca - ODP 1144^45^	0.62	4.7 e-10
Land Surface Temperature MJJAS (lat : 0/50 N, lon : 40/130 E		
speleothem *δ*18O - Hulu/Sanbao caves^56^	0.46	4.2 e-13
Land precipitation MJJAS (lat : 0/50 N, lon : 40/130 E		
speleothem *δ*18O - Hulu/Sanbao caves^56^	0.60	7.29 e-23
Land Surface Temperature MAMJJASON (local grid point)		
Lingtai loess-paleosol sequence^42^	0.41	4.4 e-5
Xifeng loess-paleosol sequence^41^	0.30	1.0 e-4
Luochuan loess-paleosol sequence^41^	-	not significant

Pearson correlation results (correlation coefficient and associated p_value with 95% confidence level) between All_forcings model outputs and geological records shown in Fig. 2. Summer (May to September - MJJAS) sea surface temperature (SST) from the model is compared with marine *δ*18O records (Fig. 2 - red symbols). Model annual mean SST are compared with Mg/Ca records (Fig. 2 - yellow symbols over the ocean). Summer (MJJAS) land surface temperature (LST) and precipitation from the model are compared with speleothem *δ*18O records (Fig. 2 - pink symbols). Simulated Land Surface Temperature (LST) during bacterial growth season (March to November; MAMJJASON) are compared with loess temperature reconstructions records (Fig. 2 - yellow symbols over land.)

Overall, the model appears to skilfully represent the Asian summer monsoon variability and the process driving orbital-scale variations. However, it remains unclear whether variability is dominated by a single forcing (ice sheet height and extent, CO2 or orbital variation) or whether the dominant driver shifts through the Quaternary.

### Disentangling each driver’s impacts on Monsoon Variability

Here we disentangle the impacts of four forcings combinations (i) All_forcings, (ii) Orb_Ghg, (iii) Orb_Ice and (iv) Orb on Asian summer monsoon variability on orbital timescales. Across the four simulation sets, the distributions of stronger ( > 3rd quantile) and weaker ( < 1st quantile) monsoon states are broadly comparable. In particular, 850 hPa wind shows similar magnitude and intensity for all sets (Supplementary Fig.1). Surface temperature patterns are also similar among the four sets (Supplementary Fig.1), as are the overturning streamfunction over the Indo-Asian and East-Asian domain (Supplementary Fig.5 and Supplementary Fig.6). Differences in precipitation and in temperature between the strongest and the weakest summer monsoons also show comparable spatial distributions (Supplementary Fig.2 and Supplementary Fig.3). However, differences in intensity are found. In particular, mean temperatures associated with both the strongest and weakest monsoon states progressively decrease from Orb (Tmin_*Orb*_ = 24.5°, Tmax_*Orb*_ = 25.8°) to Orb_Ice (Tmin_*Orb*_*Ice*_ = 24.1°, Tmax_*Orb*_*Ice*_ = 25.5°), Orb_Ghg (Tmin_*Orb*_*Ghg*_ = 22.9°, Tmax_*Orb*_*Ghg*_ = 24.9°), and All (Tmin_*All*_ = 22.6°, Tmax_*All*_ = 24.9°), with the largest temperature contrast occurring between Orb_Ice and Orb_Ghg (Supplementary Fig.1). This pattern suggests that greenhouse gas variability is the primary driver of temperature variability simulated in All_forcings.

The amplitude of change between the strongest and weakest summer monsoons also varies between the sets, with the largest amplitudes simulated in All (ΔT_*All*_ = 2.3°, ΔPrecip_*All*_ = 0.78 mm/day), compared to Orb_Ghg (ΔT_*Orb*_*Ghg*_ = 2.0°, ΔPrecip_*Orb*_*Ghg*_ = 0.75 mm/day), Orb_Ice (ΔT_*Orb*_*Ice*_ = 1.4°, ΔPrecip_*Orb*_*Ice*_ = 0.73 mm/day) and Orb (ΔT_*Orb*_ = 1.3°, ΔPrecip_*Orb*_ = 0.71 mm/day) for the weakest states, in both temperature and in precipitation (Supplementary Fig.3). The largest difference in temperature occurs between the Orb_Ghg and Orb_Ice simulations, whereas the largest precipitation contrast is found between Orb_Ghg and All_forcings. This suggests that greenhouse gases seem to play a more significant role compared to ice sheets on temperature variability, whereas the relative contributions to precipitation variability is less clear.

Additionally, while the correlation between summer latent heat flux above the Tibetan Plateau and summer monsoon precipitation (as well as with the land-sea thermal contrast) stays equally strong among the four simulation sets (Supplementary Figs.7 to 10), the correlation coefficient (r) with the spring sensible heat flux above the Tibetan plateau progressively decreases from OrbGhg (Supplementary Fig.8; r_*Orb*_*Ghg*_ = 0.69, p_value = 7.89e-32), to All_forcing (Supplementary Fig.7; r_*All*_ = 0.63, *p*_value = 6.55e-26), Orb (Supplementary Fig.9; r_*Orb*_ = 0.57, *p*_value = 1.49e-20) and Orb_Ice (Supplementary Fig.9; r_*OrbIce*_ = 0.57, p_value = 1.49e-20), suggesting an important role played by the greenhouse gases in the monsoon initiation. Consistently with this, greenhouse gases variations applied to the snapshots show significant correlation with sensible heat fluxes from both All_forcing (Supplementary Fig.7; r_*All*_ = 0.25, *p*_value = 1.74e-4) and Orb_Ghg (Supplementary Fig.8; r_*Orb*_*Ghg*_ = 0.31, *p*_value = 2.98e-6). Conversely, prescribed ice sheet volume variations shows a significant correlation coefficient with sensible heat flux in All_forcing (Supplementary Fig.7; r_*All*_ = −0.25, *p*_value = 1.64e-4) but this relation vanishes in Orb_Ice (Supplementary Fig.9; Pearson’s correlation test not significant at 95%). Nevertheless, ice sheet volume variations stronger correlates with the summer latent heat flux in Orb_Ice (Supplementary Fig.9; r_*OrbIce*_ = −0.39, *p*_value = 1.63e-09) than greenhouse gases variations in Orb_Ghg (Supplementary Fig.8; r_*OrbGhg*_ = −0.33, *p*_value = 4.97e-07), indicating a higher impact of ice sheet than greenhouse gases on monsoon intensity.

**Fig. 4:**
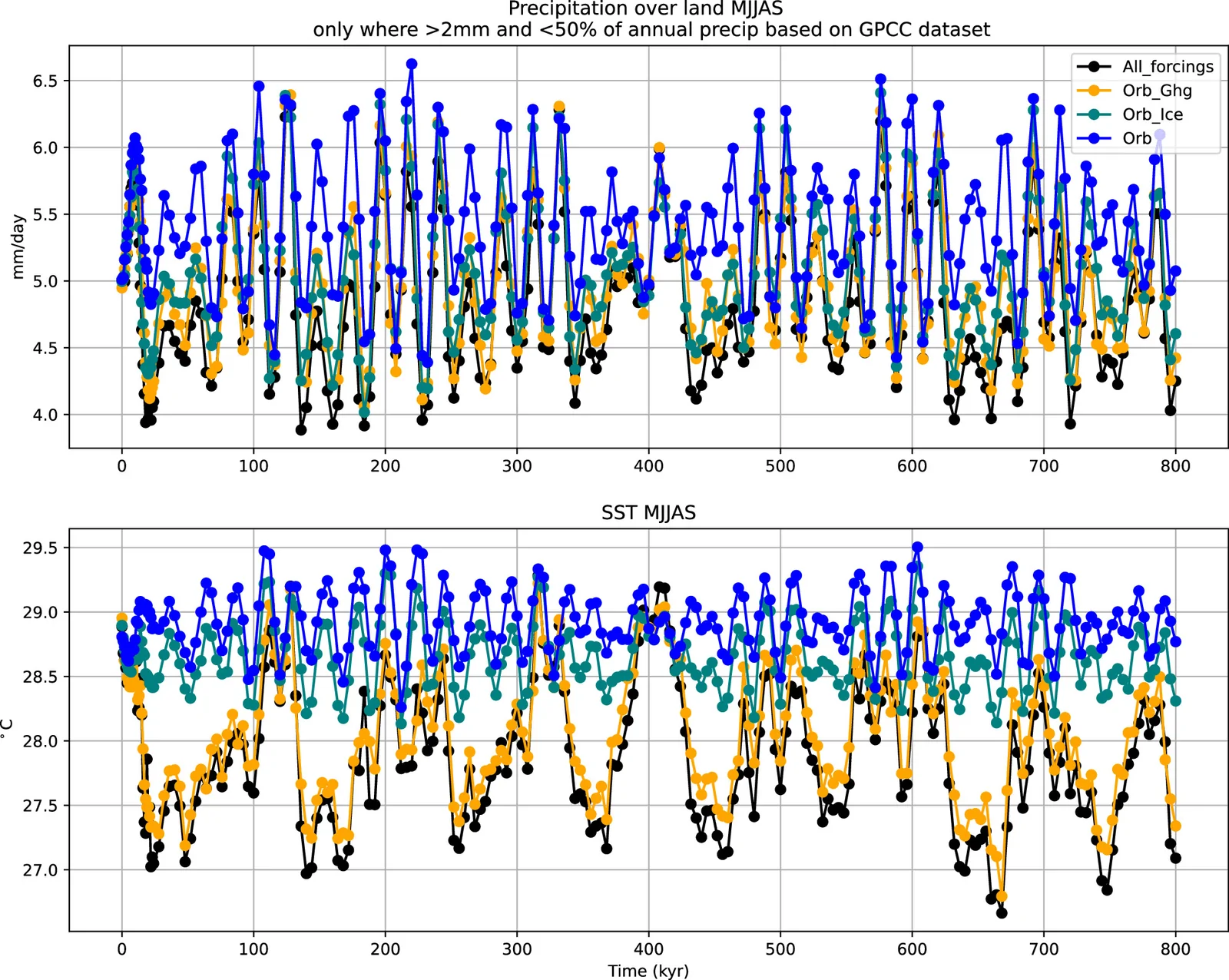
Summer monsoon Precipitation and sea surface temperature (SST) timeseries for each simulation set (May to September; MJJAS). Precipitation (mm/day) is averaged over the blue-shaded monsoon domain shown in Fig. 2 and SST is averaged over 0^∘^-50^∘^N, 60^∘^-130^∘^E. All_forcings is shown in black, Orb_Ghg in green, Orb_Ice in purple and Orb in blue.

To better understand these variations, we now focus on changes in the pacing of the Asian monsoon through time. Figure 4 shows the simulated summer monsoon precipitation (averaged over the blue-shaded area in Fig. 2 together with SST time series over the study domain for each forcings set. A clear 20-kyr periodicity is resolved in all simulations, but with some additional variability at longer,  ≈ 100-kyr periodicity. In addition, these results suggest that greenhouse gas variability exerts a strong control on SST variability at eccentricity timescales. This is illustrated by the marked decoupling between the Orb and Orb_Ghg simulations, with SSTs cooling from 28.9 ^°^C in Orb to 28.0 ^°^C in Orb_Ghg, approaching the mean SST of the All_forcings simulation (27.9 ^°^C). Adding ice-sheet forcing to the orbital forcing also leads to cooler mean SSTs, but to a lesser extent than greenhouse gas variations (28.7 ^°^C in Orb_Ice), and without altering the dominant pacing observed in the Orb timeseries.

For precipitation, both greenhouse gas and ice-sheet forcing produce a drying tendency relative to the Orb simulation, although to a lesser magnitude of drying and does not reach the mean precipitation level of the All_forcings simulations (5.4 mm/day for Orb, 5.1 mm/day for Orb_Ice, 5.0 mm/day for Orb_Ghg). The forcing set that most closely matches the mean monsoon period precipitation in All_forcing (4.8 mm/day for All) is Orb_Ghg. Taken together, the SST and precipitation response therefore suggests that greenhouse gas variability is the dominant contributor to the mean state simulated in All_forcings. This is consistent with the moist static energy (MSE) profiles in Supplementary Figs. 4 to 6, where Orb_Ghg and All_forcings exhibit the most similar structures, suggesting that greenhouse gases play a leading role in shaping Asian summer monsoon dynamics, in part by modulating the land-sea temperature gradient^21^.

**Fig. 5:**
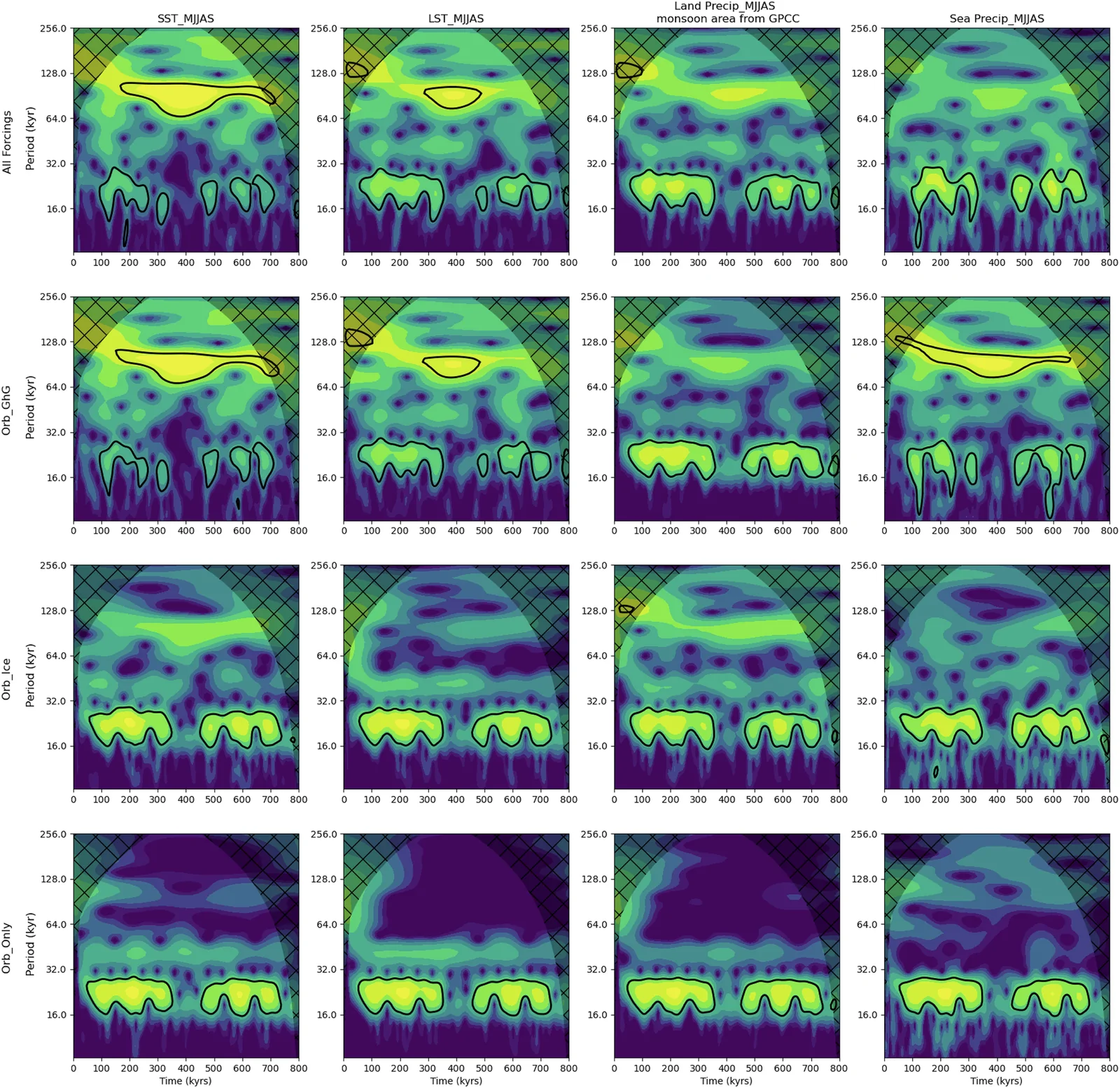
Wavelet Power Spectra for timeseries from each simulation set. Thick black line contour depicts regions at the 95% confidence level relative to a red-noise process. Shaded and hatched regions denote the cone of influence of the morlet mother wavelet. Rows show, from top to bottom, results from All_forcings, Orb_Ghg, Orb_Ice and Orb_Only simulation sets. Columns show, from left to right, summer (MJJAS) Sea Surface Temperatures (SST), summer Land Surface Temperature (LST) (calculated over the entire domain shown in (Fig. 2), summer monsoon precipitation over the land (calculated over the blue shaded area from Fig. 2) and precipitation over the sea (calculated over the entire domain of Fig. 2.

While the relative influence of each forcing can be clearly identified in SST variability by looking at simple timeseries, their individual roles are less clearly expressed in the pacing of the Asian summer monsoon precipitation over the 800-kyr period. Therefore, to further investigate the impact of each forcing on the pacing of the Asian summer monsoon, we decompose the signals into periodic components and identify the dominant modes of variability using wavelet power spectrum analysis^46^. This analysis is applied to SST, land surface temperature (LST), and monsoon precipitation time series over land (blue-shaded area in Fig. 2) and over the ocean for all simulation sets. Fig. 5 shows the wavelet power spectrum for each variable and for the four simulation sets (All_forcings, Orb_Ghg, Orb_Ice and Orb).

Wavelet analyses show significant power at precession timescales ( ≈ 21kyr) throughout the 800 kyr apart from around 400 kyr, for each variable (SST, LST, monsoon precipitation over the land and ocean precipitation), for all simulation sets. This confirms what has been observed in Fig. 4 and suggests that precession variations have a direct impact on temperature and precipitation over the domain presented in Fig. 2. This precession signal is the only significant pacing identified in the spectra in both Orb_Only and Orb_Ice (third and fourth row). When greenhouse gas variability is added to the orbital forcing (second row in Fig. 5), contrasting behaviour emerges between the sea and the land. A pronounced 100 kyr signal is appearent in simulated SSTs and precipitation over the ocean, while the precession signal weakens, although it remains significant. Eccentricity does not have a large effect on LSTs and precipitation. Although a small 100 kyr signal on the LST plot emerges at 400 kyr with the 21 kyr power slightly weakened. Precipitation over land does not change between Orb_Only and Orb_Ghg with no significant power at 100 kyr and neither a weakening of the 21 kyr period. These results suggest that eccentricity influences temperature and precipitation over the ocean indirectly through greenhouse gas variations, whereas greenhouse gas changes exert a comparatively limited influence over land within the study region.

Finally, when ice sheet variability is included with orbital parameters and greenhouse gas variations (All_Forcings Fig. 5 top row), we obtain similar power spectra as those for Orb_Ghg apart from precipitation over the sea, where no significant eccentricity signal is observed. Therefore, combining the variations of the ice sheet with greenhouse gases and orbital parameters does not strongly affect the pace of LST and precipitation.

Our results confirm the direct impact of precession variability on temperature and precipitation over the Asian region as argued previously in refs. ^47^ and^48^, implying that the Asian summer monsoon is directly paced by precession. Greenhouse gas variability drives a strong 100 kyr signal on SSTs, without requiring a direct eccentricity control. Because SST exerts a strong influence on extratropical monsoon development (Fig. 1, refs. ^21,36^), greenhouse gases appear to be a key modulator of Asian summer monsoon variability through their affect on SST. Ice sheet variations moderate the impact of precession and greenhouse gases without altering the signal’s pace, and the significant 100 kyr power in ocean precipitation is reduced or removed when ice sheet forcing is included.

**Fig. 6:**
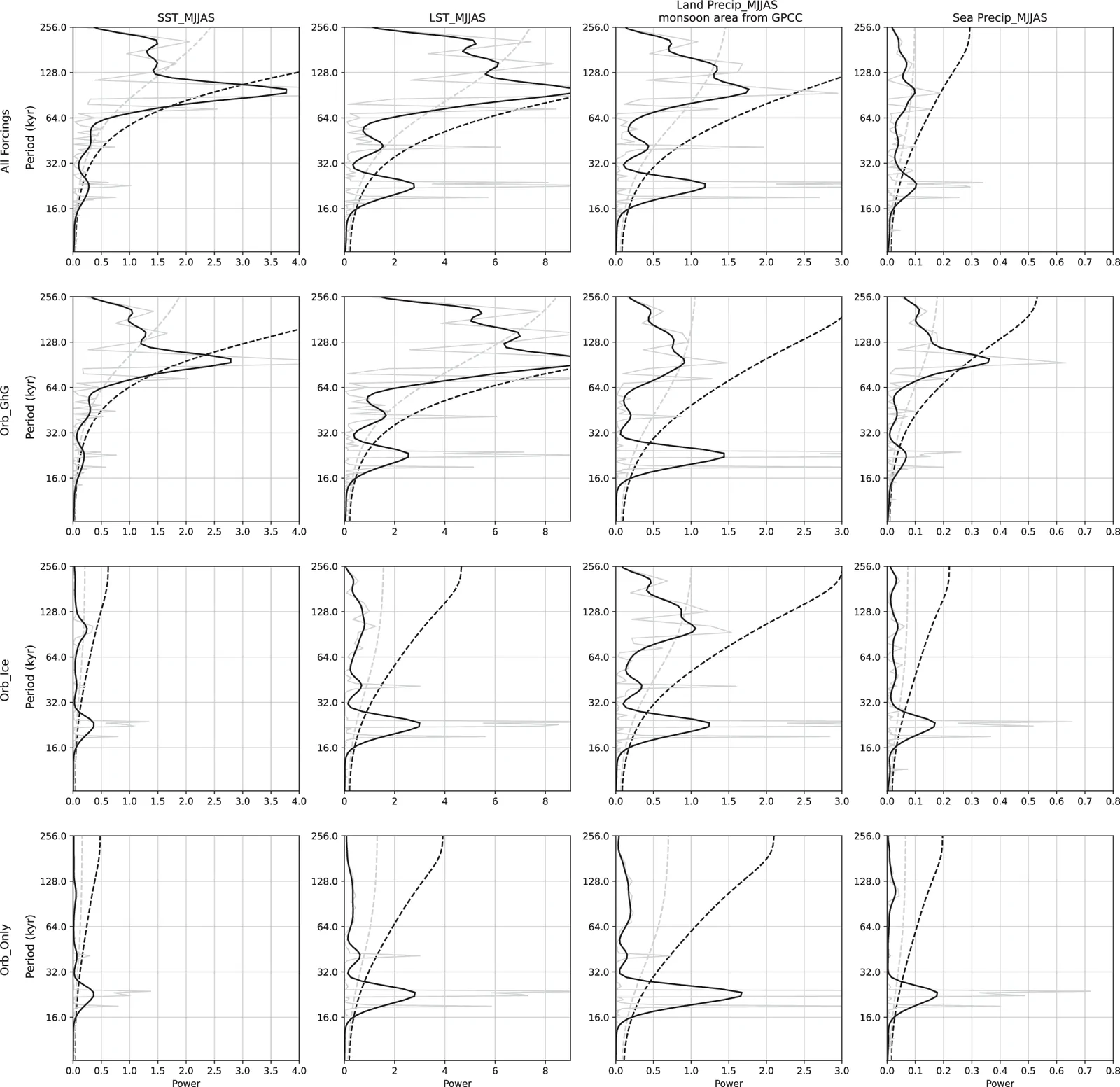
Global Wavelet Spectra corresponding to Fig. 5. The black dash lines marks the 95% confidence level. Grey solid lines are the red-noise and grey dash lines indicates the mean red-noise spectrum. As shown in Fig. 5. Rows show, from top to bottom, All_forcings simulations, Orb_Ghg, Orb_Ice and Orb_Only. Columns (left to right) shows results for SST, LST, monsoon precipitation over the land and, precipitation over the sea.

To better quantify the strength of the dominant periodicities identified for each variable timeseries and simulation set, we now focus on the global wavelet spectra Fig. 6 corresponding to the wavelet spectra shown in Fig. 5. Overall, spectral power integrated over the 800 kyr interval is weaker Fig. 6 for precipitation (especially for ocean precipitation) than for temperatures. Therefore, in our simulations which encompass a large Asian domain Fig. 2, precipitation variability is influenced to a lesser extent by direct or indirect orbital variations compared to temperatures. Additionally, the global wavelet spectrum for land precipitation reveals an emerging peak at  ≈ 100 kyr for both Orb_Ice and Orb_Ghg which is also visible and stronger for All_forcings, although not strong enough to be significant. Because summer monsoon circulation patterns differs markedly between India and East Asia (Supplementary Figs. 4 and 5), averaging precipitation over a broad region likely dampens consistent signals. In this respect, Supplementary Fig.11 shows precipitation time series for four subdomains and reveals a similar decoupling between simulation sets as observed for the SSTs (Fig. 4) over the southeastern Asian summer monsoon domain, but not over the Western or Northern subdomain where precession remains the dominant period for each set. The similarity in variability between SST and precipitation over Southeast Asia is consistent with the well-documented thermal forcing of summer monsoon precipitation in this region^34^.

### Discussion

Numerous geological records collected across the Asian monsoon domain have highlighted the impact of Milankovitch cycle variability. However, only a few modelling studies^24^^[,49^ have investigated the individual impact of the drivers of the Asian monsoon’s pace. Our simulations provide valuable insights into this subject, indicating that both precession and greenhouse gas variability directly shape the pacing of the Asian summer monsoon. This challenges the commonly accepted paradigm that Quaternary Asian monsoon variability is driven primarily by changes in ice sheet extent^17^^[,14^^[,50^^[,51^^[,52^^[,27^^[,23^. However, our results do not contradict such studies because they emphasise the role of ice sheets on monsoon dynamics and intensity for one (or a small handful of) time-slices whereas our study focuses on monsoon pacing across the whole of the past 800 kyr. Consistent with this distinction, we also find that ice sheet forcing modulates the amplitude of the monsoon response (Supplementary Figs. 1–3).

When performing singular “snapshot" simulations, it is relatively simple to isolate the importance of different forcings and detailed mechanisms which impact the amplitude/intensity of the monsoon system at a particular time. It is more challenging to isolate the detailed mechanisms which control the pacing of the monsoon. Our simulations have highlighted the importance of greenhouse gases, and shown that precipitation over the entire region is strongly correlated with the land-ocean temperature gradient. However, our analysis encompasses a wide domain, whereas the Asian summer monsoon comprises two distinct subsystems (South Asian sector and East Asian sector) which differ in their dynamics and are often studied separately^24^^,^^27^. Consequently, direct comparison between this study and previous modelling efforts, which cover different time ranges and geographical scales, is not straightforward.

Nevertheless, a few previous modelling studies have attempted to examine the pacing of the Asian monsoon. Lyu et al.^24^ adopted a timeseries approach similar to ours, examining the respective roles of insolation, greenhouse gases, and ice sheet on the East Asian summer monsoon pace through the last 800 kyr. Their analysis however used an emulator trained on significantly fewer (61 in total) snapshot simulations of HadCM3. Additionally, Su et al.^49^ have recently published four 800-kyr long accelerated forcing transient simulations (one (ALL) including orbital parameters, ice sheet and greenhouse gas variability, as well as a set of accelerated forcing transient sensitivity simulation sets (ORB, ICE and CO2)) using CESM1.2.2 Earth System Model. These simulations use 100x acceleration of the forcings and do not change the land sea mask or river routing. The model did not include vegetation feedbacks.

**Fig. 7:**
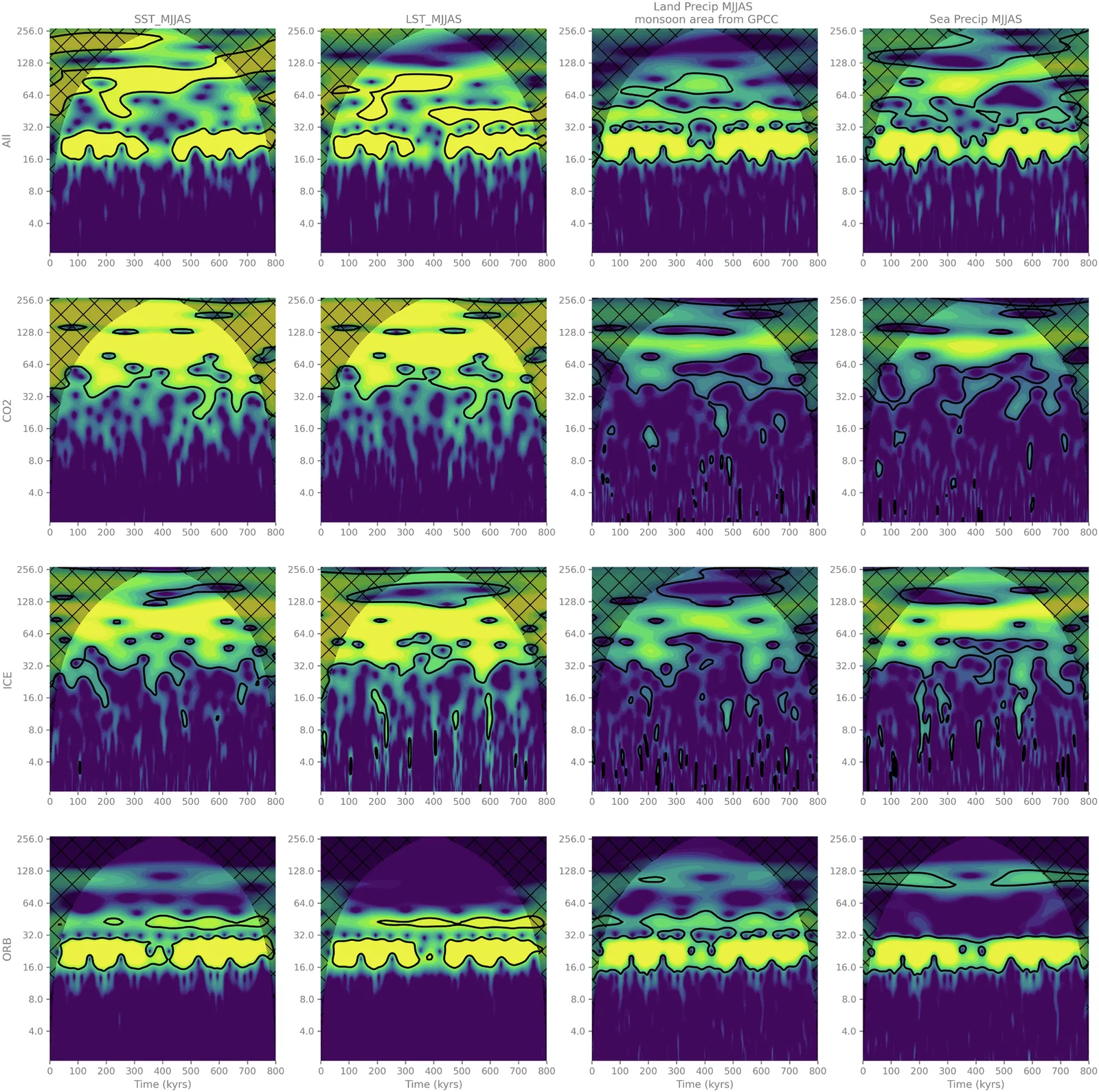
Wavelet Power Spectrum from accelerated forcing transient simulations using CESM1.2 ^49^(0^∘^–/50^∘^N, 60^∘^/130^∘^E). Thick black contours indicate regions significant at the 95% confidence level as determined by a red-noise process. Shaded and hatched areas denote the cone of influence of the mother wavelet. Rows show, from top to bottom, the All, CO2, ICE, and ORB experiments. Columns show, from left to right, SST, LST, monsoon precipitation over land, and precipitation over the ocean.

These studies allow us to perform an ad hoc model uncertainty analysis of our results. We perform wavelet spectra analysis for the simulations of Su et al.^49^ (Fig. 7) which can be compared to our results (Fig. 5). The top and bottom row of both figures are directly comparable but the middle two rows in Fig. 7) are not directly comparable because Su et al.^49^ perform simulations with ice and CO2 forcing only whereas we include orbital forcing in our simulations.

Overall, there is good agreement between the two sets of model results from the all forcing and orbit only simulations. Both show considerable power for all variables at  ≈ 20 kyr. The orbit only simulation show relatively weak power at  ≈ 100 kyr periods whereas the all forcing simulations show much stronger response. Similarly, all simulation show the reduction in precession power at around 400 kyr BP (due to the Earth’s orbit being near circular then). Comparing the CO2 and ice forcings, all simulations confirm that the greenhouse gases’s produce the strongest temperature power at 100 kyr, thus confirming one of our main conclusions. The response in precipitation is more complicated but both simulations show more comparable amplitude response at 100 kyr.

The biggest differences are that our simulations show more power at 100 kyr for ocean precipitation from greenhouse gases forcing, whereas the Su et al.^49^ simulation shows the 100 kyr power spectrum for ocean precipitation to be more forced by ice sheets. The reasons for such differences are hard to determine. They could be related to model structure (i.e., HadCM3B which includes convective parameterisation improvements, interactive vegetation and higher horizontal resolution in the ocean whereas CESM1.2.2 uses fixed vegetation but higher vertical resolution in the ocean), ice sheet forcing (HadCM3B simulations use global ice sheet reconstructions from de Boer et al.^53^, CESM1.2.2 uses a different reconstruction and for the Northern Hemisphere only), or from the different simulation method (multiple snapshots versus accelerated forcing). The snapshot approach assumes that for each time slice, climate is in equilibrium with the imposed forcing. This is likely to be valid for the atmosphere and near surface ocean but not for the deeper ocean. Similarly, accelerated forcing simulations can bias aspects of the climate response due to the long adjustment times for forcings (deep ocean, ice sheets, carbon cycle), especially when ice sheet variability is a major driver^54^^55^. It can also distort aspects of the spectrum because climate models have some natural variability at decadal to centennial scales which can then get interpreted as 100x longer time scale. Su et al.^49^ acknowledge that the pacing and intensity of the Asian summer monsoon response may differ between non-accelerated forcings (as well as more reduced accelerated factor) and snapshot model configurations.

These limitations are important to consider when investigating ocean circulation, which has a longer memory than the atmosphere. Previous studies have highlighted a tight linkage between the Atlantic Meridional Overturning Circulation (AMOC) and the Asian monsoon variability^56^^[,57^. As such, an inaccurate representation of the ocean’s memory may affect the Asian monsoon’s response. In particular, Liu et al.^58^ demonstrated, using CESM1.2.2 like Su et al.^49^, that accelerating orbital forcing with only a factor of 2 in transient simulations can substantially distort AMOC variability. On the other hand, our snapshot simulations do not have a fully equilibrated ocean (would require to run during about 3500 model years^58^) and have no memory of past ocean states, which can affect the AMOC. Only a non-accelerated transient simulation could adequately represent the interactions between AMOC and Asian monsoon. However, because each snapshot has been run independently, biases remain confined to individual simulations rather than accumulating throughout the time series, unlike in transient simulations with accelerated forcings.

**Fig. 8:**
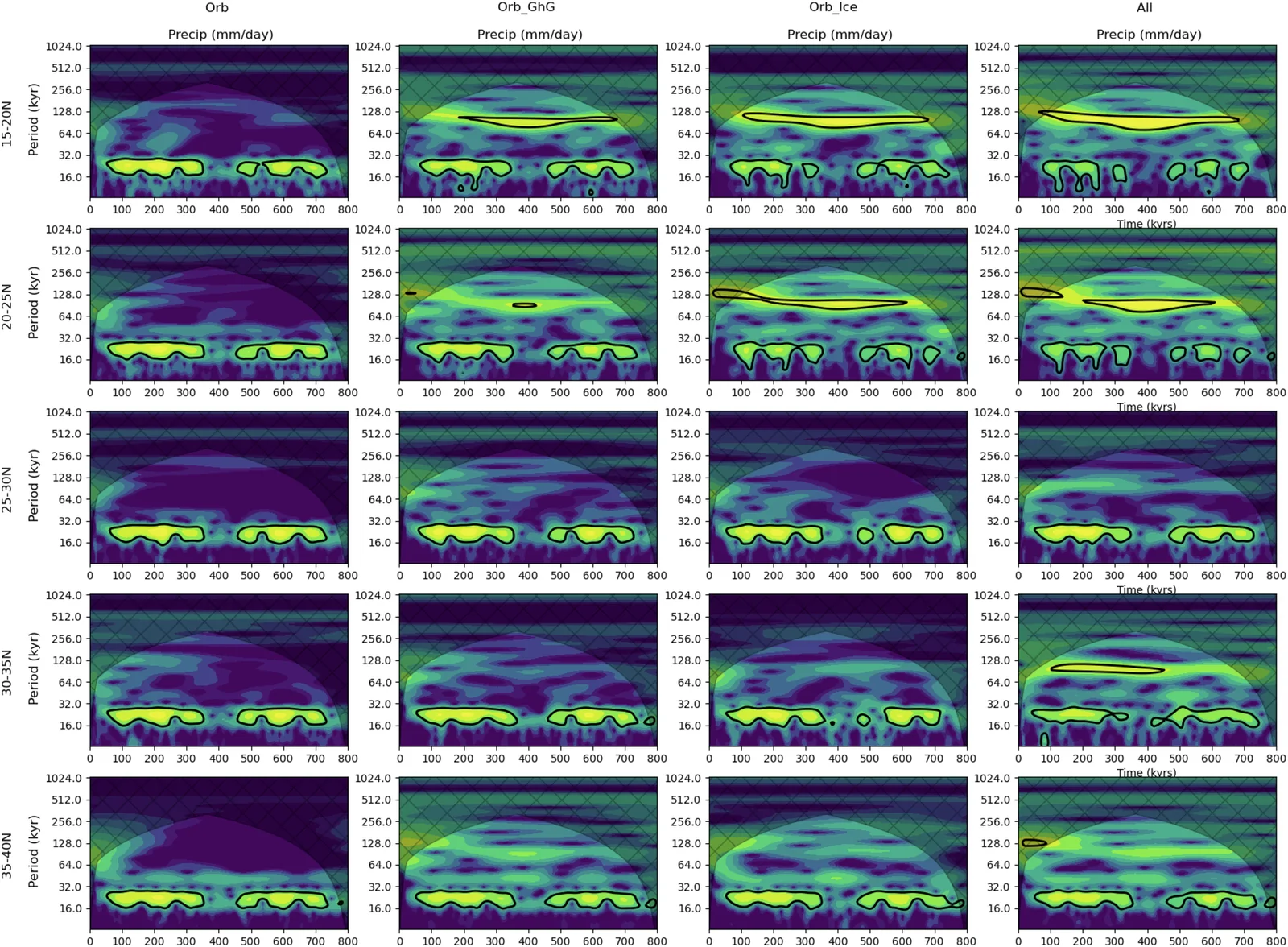
Wavelet power spectrum for June–July–August precipitation (15^∘^–40^∘^N, 105^∘^/120^∘^E). Thick black contours denote regions significant at the 95% confidence level as determined by a red-noise process. Shaded and hatched areas indicate the cone of influence of the mother wavelet. Columns show, from left to right, Orb_Only, Orb_Ghg, Orb_Ice and All_forcings, respectively. Rows show, from top to bottom, the latitude bands 15^∘^–20^∘^N, 20^∘^–25^∘^N, 25^∘^–30^∘^N, 30^∘^–35^∘^N and 35^∘^–40^∘^N.

We next narrow our analysis of the East Asian area to compare our simulations with Lyu et al.^24^. Lyu et al.^24^ analysed Asian summer monsoon precipitation spanning 105^°^E to 120^°^E and from 15^°^N to 40^°^N (subsequently sub-divided into five smaller domains) using a wavelet spectrum analysis of summer precipitation (defined as JJA) time series driven by different forcings. They found that ice sheets play a significant role in generating 100-kyr cycles on the southern region of the domain whereas CO_2_ appears to have an insignificant effect. They also found a strong precessional signal. To allow like-for-like comparisons, we reproduce their analyses for June-July-August precipitation, shown in Fig. 8.

Our results show a comparable pattern in our Orb power spectrum, with significant  ≈ 20-kyr power across the whole domain Fig. 8. Additionally, our All_forcings power spectrum is very similar to the OrbCO_2_Ice reported by Lyu et al.^24^. A persistent 100-kyr signal emerges in the southern part of the East Asian region (15–20^°^N), as well as a precession signal across the entire domain Fig. 8. Both simulations also exhibit eccentricity signals from both the OrbCO2 and OrbIce forcing at low latitudes (15–20N, and 20–25N) which diminishes further poleward, although the relative strengths are different. Both suggest that the ice sheet is stronger than the CO2 effect, but for our model the difference is small whereas their simulations show a dominance of the ice sheet. There are some further differences between the simulations. For instance, an eccentricity signal is apparent in our All_forcings results at 30–35^°^N, whereas it is not apparent in the equivalent simulation by Lyu et al.^24^. Similarly, there is only a weak precession signal at 20–25N in the Lyu et al. OrbIce simulation which is not the case for our simulation. Surprisingly, they also see strong precession signal at around 400kyr despite the orbit being near circular.

These results show that the response of the Asian summer monsoon to orbital variability depends on spatial scale, and that the combination of driving forcings can vary across regions. They support the interpretation that greenhouse gas variability is an important contributor to the 100-kyr cycle on the Asian summer monsoon, though its strength depends on the location and variable being considered. Our results are consistent at the regional scale, showing that variability at lower latitudes is more sensitive to eccentricity-scale forcing than at higher latitudes in western Asia. This confirms that the 100-kyr signal is more pronounced closer to the ocean than over land, as suggested by Fig. 6 and Supplementary Fig.12.

Several methodological differences between Lyu et al.^24^ and our study can potentially explain the disagreement of the impact in greenhouse gas forcing. First, Lyu et al.^24^ reconstructed their 800-kyr time series from an emulator trained on only 61 HadCM3 snapshots simulations without any vegetation feedbacks. We use direct simulation and run an improved version of the model to cover the whole 800-kyr with 219 individual snapshot simulations with time-specific boundary conditions and a model that represents vegetation change. The emulator used in Lyu et al.^24^ was constructed from a model version that doesn’t include the paleoconditioning detailed in ref. ^31^. This conditioning, by improving the model convection scheme, significantly improves the representation of African monsoon precipitation and dynamics^31^ and also better represents Asian monsoon behaviour (Fig. 1, further improving proxy-model comparison Table 2). Additionally, their emulator uses a single metric to represent ice sheet variations whereas, in reality, the Laurentide and Fennoscandinavian ice sheets vary in different ways at different times. Our full climate model simulations are able to represent such diverse variability.

### Summary and prospects

Using a unique set of 219 snapshots simulations covering the last 800 kyr, we demonstrate the ability of the improved version of HadCM3B model to accurately reconstruct Asian monsoon system variability on orbital timescales in broad agreement with proxy observations. Our results reveal a strong correlation between summer monsoon precipitation and land-sea thermal contrast in the northern hemisphere summer, consistent with a thermally driven monsoon response.

In addition, we performed a set of targeted sensitivity experiments to isolate the drivers of Asian summer monsoon variability, using four distinct simulation sets: orbital forcing only, orbital forcing plus ice-sheet variability, orbital forcing plus greenhouse gas variability, and lastly, all three forcings combined. Wavelet analysis of four variables (SST, LST, summer monsoon precipitation over land, and precipitation over the ocean) reveals a dominant precession signal (23-kyr) linked to orbital forcing for all variables, and an eccentricity-scale (100-kyr) signal associated with greenhouse gas variability through its direct effect on the land-sea thermal contrast and spring uplifted sensible heating. Adding ice-sheet variations to orbital and greenhouse gas forcing has little impact on the large-scale monsoon pacing but does help explain regional variability over Southeast Asia. Overall, these results indicate that the long-term pacing of the Asian summer monsoon is governed primarily by precession and greenhouse gas variability, whereas ice sheets play a secondary role, mainly damping the amplitude of variability through its impact on the summer uplifted latent heat flux rather than altering its dominant pace.

While our analysis focuses on the large-scale Asian summer monsoon response, the response to orbital variability is more complex at regional scales. This underscores that care is needed when comparing and interpreting signals across different sites and proxy types, where local dynamics and sensitivities can modulate the apparent pacing and amplitude of monsoon variability.

This work demonstrates the value of large ensembles of equilibrium snapshot simulations covering varying climate states through the Quaternary to investigate orbital-scale climate variability in particular to pursue the study of time dependant, orbital-scale changes in the Asian summer monsoon. These results also motivate further process-based analysis to explore how orbital-scale forcings shape atmospheric dynamics that underpin monsoon behaviour. Such efforts should help to explain the regional heterogeneity recorded in geological archives to improve the robustness of model-proxy comparisons.

## Methods

### Model description—HadCM3B

The simulations presented here are performed with a recently updated version of HadCM3B, a variant of the Hadley Centre unified model^59^^,^^60^. HadCM3B is a coupled atmosphere-ocean-vegetation model. The specific version used for this work, HadCM3B-M2.1aD is described in detail in refs. ^30^^,^^31^. The atmosphere has a horizontal resolution of 2.5^°^x 3.75^°^ and 19 vertical levels and 1.25^°^x 1.25^°^ in the ocean with 20 vertical levels with finer resolution near the surface. Despite its relatively coarse resolution and low complexity compared to recent state-of-the-art CMIP6 models, HadCM3 has been shown to accurately simulate the modern climate system (sitting in the middle of the CMIP6 pack^30^) as well as Quaternary and Deep-time climate^31^^[,61^. Its main advantage is its computational efficiency which permits long simulations and large ensemble and sensitivity studies. The model includes a land surface scheme MOSES2.1^62^ which simulates energy fluxes and physiological processes. Nine surface types are incorporated by MOSES2.1 and are simulated by the vegetation component Top-down Representation of Interactive Foliage and Flora Including Dynamics (TRIFFID)^63^. No interactive greenhouse gases cycle or ice model are included in this study, thus they are imposed by the boundary conditions described below. Moreover, the model version used here has been updated to include paleoclimate-informed changes to atmospheric convection and dynamic vegetation as described in ref. ^61^. This update allows the model to simulate more accurately Holocene tipping points (recorded by geological data), without affecting its performance in simulating pre-industrial climate^31^). Furthermore, HadCM3 was found to be one of the best performing models for reproducing key features of the Asian monsoon system compared to CMIP5 models^32^.

### Boundary conditions and experimental design

The simulations as well as the boundary conditions for each time slice are as described in ref. ^64^, see paper for more details. A brief description is given here.

The simulations have been forced by orbital parameters variations based on ref. ^65^). Ice sheets are treated as a model boundary condition for each period based on the reconstruction from ref. ^53^. Land-sea distribution, topography, ice sheet extent and river routing are also modified based on the ice sheet reconstruction. Greenhouse gas concentrations (CO_2_, CH_4_ and N_2_O) were taken from Vostok Ice Core^66^,^67^,^68^. These boundary conditions have been included in 219 snapshot experiments to span 800 kyr from pre-industrial. For the first 24 kyr BP, these are set at 1 kyr intervals then, from 24 kyr until 800 kyr BP at 4 kyr intervals. Each simulation has been run for 500 years reaching a quasi-equilibrium state for the atmosphere and near surface ocean. The last 100 years of each simulation were used to calculate the climate means.

In order to identify the main drivers between the three different forcings of the Asian monsoon variability at orbital time scales, four sensitivity snapshot sets were performed. The first one (All_forcings) includes the all three forcings (orbital variations, greenhouse gas variations and variable ice sheet elevation and extent). The second one (Orb_Ghg) incorporates both orbital and greenhouse gas forcings but a constant pre-industrial ice sheet extent and elevation. The third one (Orb_Ice) has orbital and ice sheet forcing but constant pre-industrial greenhouse gas concentrations ([*CO*_2_] = 280ppm, [*CH*_4_] = 760 ppbv, [*N*_2_*O*] = 270 ppbv). Finally, the fourth (Orb_Only) set only includes orbital variations, with constant pre-industrial greenhouse gases and constant pre-industrial ice sheet. All sets characteristics are summarised in Tab. 1.

### Definition of the Asian summer monsoon area

Our study area is shown in Fig. 2. The monsoon land precipitation area corresponds to the blue shaded field on the figure and has been defined using the Global Precipitation Climatology Centre (GPCC) between 1951 and 2000^69^ (the less recent record to avoid most of the effects of recent climate change). GPCC data was then regridded onto the HadCM3 resolution. More precisely, we have adopted a monsoon definition that is used within the latest IPCC AR6 report whereby summer precipitation (between May and September) exceed 55% of the annual total and local summer minus winter precipitation exceed 2.0 mm/day^70^. For the other variables used in this study, such as land and sea surface temperature, as well as precipitation over the sea, the entire domain in Fig. 2 is taken into account.

We do not account for the paleo-calendar effect^71^ when averaging variables across the summer months. However, we performed additional analyses (not shown) including this effect and found that its impact in this study is minimal and largely smoothed out (smaller than the interannual variability), because we average over five months and focus on mid to low latitude regions.

## Supplementary information

Supplementary information

Transparent Peer Review file

## Data Availability

The outputs of the 876 (4 x 219) simulations used to support the findings of this study are available on Zenodo through the following link: https://doi.org/10.5281/zenodo.21310840.

## Code availability

The Python scripts elaborated to perform the analysis supporting the results of this paper are available on a public repository and can be access via the following link: https://www.paleo.bristol.ac.uk/ummodel/scripts/papers/software/Millot-Weil_et_al_2025_monsoon_codes.tar.

## References

[1] Sun, Y. et al. A review of orbital-scale monsoon variability and dynamics in East Asia during the Quaternary. *Quat. Sci. Rev.* **288**, 107593 (2022).

[2] Zhisheng, A. et al. Global monsoon dynamics and climate change. *Annu. Rev. Earth Planet. Sci.* **43**, 29–77 (2015).

[3] Liu, T., Ding, Z. & Rutter, N. Comparison of Milankovitch periods between continental loess and deep sea records over the last 2.5 Ma. *Quat. Sci. Rev.* **18**, 1205–1212 (1999).

[4] Liu, C. et al. Eccentricity forcing of east asian monsoonal systems over the past 3 million years. *Proc. Natl. Acad. Sci. USA* **118**, e2107055118 (2021). 10.1073/pnas.2107055118 PMC8639371 34670836

[5] Wang, Y. et al. Millennial- and orbital-scale changes in the east asian monsoon over the past 224,000 years. *Nature* **451**, 1090–1093 (2008). 10.1038/nature06692 18305541

[6] Li, X. et al. Loess deposits in the low latitudes of East Asia reveal the ~20-kyr precipitation cycle. *Nat. Commun.* **15**, 1023 (2024). 10.1038/s41467-024-45379-9 PMC10838313 38310099

[7] Tian, J., Wang, P., Cheng, X. & Li, Q. Astronomically tuned pliĉpleistocene benthic n18o record from South China Sea and atlantiĉpaci¢c comparison. *Earth Planetary Sci. Lett.* **203**, 1015–1029 (2002).

[8] Sun, Y., Clemens, S. C., An, Z. & Yu, Z. Astronomical timescale and palaeoclimatic implication of stacked 3.6-myr monsoon records from the Chinese loess plateau. *Quat. Sci. Rev.* **25**, 33–48 (2006).

[9] Cheng, H. et al. Orbital-scale asian summer monsoon variations: paradox and exploration. *Sci. China Earth Sci.* **64**, 529–544 (2021).

[10] Chen, J., Farrell, J. W., Murray, D. W. & Prell, W. L. Timescale and paleoceanographic implications of a 3.6 m.y. oxygen isotope record from the northeast indian ocean (Ocean Drilling Program site 758). *Paleoceanography* **10**, 21–47 (1995).

[11] Petit, J. R. et al. Climate and atmospheric history of the past 420,000 years from the Vostok ice core, Antarctica. *Nature* **399**, 429–436 (1999).

[12] Halley, E. An historical account of the trade winds, and monsoons, observable in the seas between and near the tropicks, with an attempt to assign the physical cause of the said winds. *Philos. Trans. R. Soc. Lond.* **16**, 153–168 (1686).

[13] Prell, W. L. & Kutzbach, J. E. Sensitivity of the indian monsoon to forcing parameters and implications for its evolution. *Nature* **360**, 647–652 (1992).

[14] Felzer, B., III, T. W. & Oglesby, R. J. The impact of ice sheets, co, and orbital insolation on late Quaternary climates: sensitivity experiments with a general circulation model. *Quaternary Sci. Rev.* **17**, 507–534 (1998).

[15] Braconnot, P., Marzin, C., Gregoire, L., Mosquet, E. & Marti, O. Monsoon response to changes in Earth’s orbital parameters: comparisons between simulations of the Eemian and of the Holocene. *Clim. Past* **4**, 281–294 (2008).

[16] Prell, W. L. & Kutzbach, J. E. Monsoon variability over the past 150,000 years. *J. Geophys. Res. Atmos.* **92**, 8411–8425 (1987).

[17] Kutzbach, J. E. & Guetter, P. J. The influence of changing orbital parameters and surface boundary conditions on climate simulations for the past 18 000 years. *J. Atmos. Sci.* **43**, 1726–1759 (1986).

[18] Gadgil, S. The monsoon system: Land–sea breeze or the ITCZ? *J. Earth Syst. Sci.* **127**, 1 (2018).

[19] Geen, R., Bordoni, S., Battisti, D. S. & Hui, K. Monsoons, ITCZs, and the concept of the global monsoon. *Rev. Geophys.* **58**, e2020RG000700 (2020).

[20] Zhang, S., Qu, X., Huang, G. & Hu, P. Asymmetric response of south asian summer monsoon rainfall in a carbon dioxide removal scenario. *npj Clim. Atmos. Sci.* **6**, 10 (2023).

[21] Ma, J. & Yu, J. Paradox in south asian summer monsoon circulation change: Lower tropospheric strengthening and upper tropospheric weakening. *Geophys. Res. Lett.* **41**, 2934–2940 (2014).

[22] Kutzbach, J. E. Monsoon climate of the early Holocene: climate experiment with the Earth’s orbital parameters for 9000 years ago. *Science* **214**, 59–61 (1981). 10.1126/science.214.4516.59 17802573

[23] Gao, Y., Liu, Z. & Lu, Z. Dynamic effect of last glacial maximum ice sheet topography on the east asian summer monsoon. *J. Clim.* **33**, 6929–6944 (2020).

[24] Lyu, A. Q., Yin, Q. Z., Crucifix, M. & Sun, Y. B. Diverse regional sensitivity of summer precipitation in East Asia to ice volume, CO _2_ and astronomical forcing. *Geophys. Res. Lett.* **48**, e2020GL092005 (2021).

[25] Lu, H. et al. Variation of east asian monsoon precipitation during the past 21 k.y. and potential CO2 forcing. *Geology* **41**, 1023–1026 (2013).

[26] Yin, Q. Z., Berger, A. & Crucifix, M. Individual and combined effects of ice sheets and precession on MIS-13 climate. *Clim. Past* **5**, 229–243 (2009).

[27] Araya-Melo, P. A., Crucifix, M. & Bounceur, N. Global sensitivity analysis of the indian monsoon during the Pleistocene. *Climate* **11**, 45–61 (2015).

[28] Liu, Z. et al. Chinese cave records and the East Asia summer monsoon. *Quat. Sci. Rev.* **83**, 115–128 (2014).

[29] Sun, Y. et al. Diverse manifestations of the mid-Pleistocene climate transition. *Nat. Commun.* **10**, 352 (2019). 10.1038/s41467-018-08257-9 PMC6341081 30664647

[30] Valdes, E. et al. The BRIDGE HadCM3 family of climate models: HadCM3@bristol v1.0. *Geosci. Model Dev.* **10**, 3715–3743 (2017).

[31] Hopcroft, P. O. & Valdes, P. J. Paleoclimate-conditioning reveals a North Africa land–atmosphere tipping point. *Proc. Natl. Acad. Sci. USA* **118**, e2108783118 (2021). 10.1073/pnas.2108783118 PMC8609301 34725155

[32] Sperber, K. R. et al. The asian summer monsoon: an intercomparison of CMIP5 vs. CMIP3 simulations of the late 20th century. *Clim. Dyn.* **41**, 2711–2744 (2013).

[33] Wang, P. X. et al. The global monsoon across time scales: mechanisms and outstanding issues. *Earth-Sci. Rev.* **174**, 84–121 (2017).

[34] Ding, Y. & Chan, J. C. L. The east asian summer monsoon: an overview. *Meteorol. Atmos. Phys.* **89**, 117–142 (2005).

[35] Sampe, T. & Xie, S.-P. Large-scale dynamics of the meiyu-baiu rainband: environmental forcing by the westerly jet*. *J. Clim.* **23**, 113–134 (2010).

[36] Walker, J. M. & Bordoni, S. Onset and withdrawal of the large–scale south asian monsoon: A dynamical definition using change point detection. *Geophys. Res. Lett.* **43** 10.1002/2016GL071026 (2016).

[37] Diaz, M. & Boos, W. R. Monsoon depression amplification by moist barotropic instability in a vertically sheared environment. *Q. J. R. Meteorol. Soc.* **145**, 2666–2684 (2019).

[38] Li, J. et al. Impacts of surface sensible heating over the Tibetan Plateau on cloud fraction and surface radiation budget in surrounding Asia. *J. Geophys. Res. Atmos.* **131**, e2025JD045385 (2026).

[39] Wu, G. X. et al. Multi-scale forcing and the formation of subtropical desert and monsoon. *Annales Geophys.* **27**, 3631–3644 (2009).

[40] Pisias, N. & Moore, T. The evolution of Pleistocene climate: a time series approach. *Earth Planet. Sci. Lett.* **52**, 450–458 (1981).

[41] Lu, H. et al. 800-kyr land temperature variations modulated by vegetation changes on chinese loess plateau. *Nat. Commun.* **10**, 1958 (2019). 10.1038/s41467-019-09978-1 PMC6488643 31036861

[42] Lu, H. et al. Decoupled land and ocean temperature trends in the early-Middle Pleistocene. *Geophys. Res. Lett.* **49**, e2022GL099520 (2022).

[43] Rahman, A., Khan, M. A., Singh, A. & Kumar, S. Hydrological characteristics of the bay of bengal water column using 18O during the indian summer monsoon. *Continental Shelf Res.* **226**, 104491 (2021).

[44] Wang, Y. J. et al. A high-resolution absolute-dated late Pleistocene monsoon record from Hulu Cave, China. *Science* **294**, 2345–2348 (2001). 10.1126/science.1064618 11743199

[45] Wei, G., Deng, W., Liu, Y. & Li, X. High-resolution sea surface temperature records derived from foraminiferal mg/ca ratios during the last 260 ka in the northern South China Sea. *Palaeogeogr. Palaeoclimatol. Palaeoecol.* **250**, 126–138 (2007).

[46] Torrence, C. & Compo, G. P. A practical guide to wavelet analysis. *Bull. Am. Meteorol. Soc.* **79**, 61–78 (1998).

[47] Clemens, S., Prell, W., Murray, D., Shimmield, G. & Weedon, G. Forcing mechanisms of the indian ocean monsoon. *Nature* **353**, 720–725 (1991).

[48] Bosmans, J. et al. Response of the asian summer monsoons to idealized precession and obliquity forcing in a set of GCMs. *Quat. Sci. Rev.* **188**, 121–135 (2018).

[49] Su, B., Sun, Y. & Zhou, M. Orbital-accelerated transient simulations of glacial-interglacial climate cycles for the last 800,000 years. *Sci. Data* **12**, 961 (2025). 10.1038/s41597-025-05297-x PMC12149303 40490441

[50] Yanase, W. & Abe-Ouchi, A. The LGM surface climate and atmospheric circulation over East Asia and the North Pacific in the PMIP2 coupled model simulations. *Clim. Past* **3**, 439–451 (2007).

[51] Braconnot, P. et al. Results of PMIP2 coupled simulations of the mid-Holocene and last glacial maximum –part 1: experiments and large-scale features. *Climate* **3**, 261–277 (2007).

[52] Qiuzhen Yin, A., Driesschaert, E., Goosse, H., Loutre, M. F. & Crucifix, M. The Eurasian ice sheet reinforces the east asian summer monsoon during the interglacial 500,000 years ago. *Climate* **4**, 79–90 (2008).

[53] De Boer, G., Bauer, S. E., Toto, T., Menon, S. & Vogelmann, A. M. Evaluation of aerosol-cloud interaction in the GISS ModelE using ARM observations. *J. Geophys. Res. Atmos.* **118**, 6383–6395 (2013).

[54] Lunt, D. J., Williamson, M. S., Valdes, P. J., Lenton, T. M. & Marsh, R. Comparing transient, accelerated, and equilibrium simulations of the last 30,000 years with the GENIE-1 model. *Clim. Past* **2**, 221–235 (2006).

[55] Varma, V., Prange, M. & Schulz, M. Transient simulations of the present and the last interglacial climate using the community climate system model version 3: effects of orbital acceleration. *Geosci. Model Dev.* **9**, 3859–3873 (2016).

[56] Cheng, H. et al. The asian monsoon over the past 640,000 years and ice age terminations. *Nature* **534**, 640–646 (2016). 10.1038/nature18591 27357793

[57] Dong, X. et al. Early warning signals for asian summer monsoon tipping and implications for future monsoon changes. *Innov. Geosci.* **3**, 100158 (2025).

[58] Liu, H. et al. Collapse of the atlantic meridional ocean circulation induced by precession: sensitivity to orbital acceleration. *Geophys. Res. Lett.* **52**, e2025GL115941 (2025).

[59] Pope, V. D., Gallani, M. L., Rowntree, P. R. & Stratton, R. A. The impact of new physical parametrizations in the Hadley Centre climate model: HadAM3. *Clim. Dyn.* **16**, 123–146 (2000).

[60] Gordon, C., Gregory, J. M. & Wood, R. A. The simulation of SST, sea ice extents and ocean heat transports in a version of the Hadley Centre coupled model without flūx adjustments. *Clim. Dynamics* **16**, 147–168 (2000).

[61] Hopcroft, P. O., Valdes, P. J. & Ingram, W. Using the mid-Holocene ’greening’ of the Sahara to narrow acceptable ranges on climate model parameters 10.1002/essoar.10505398.1 (2020).

[62] Essery, R., Best, M. & Cox, P. *MOSES 2.2 Technical Documentation. Hadley Center technical note 30* (2001).

[63] Cox, P. Description of the TRIFFID dynamic global vegetation model. *Hadley Centre Technical Note* 24 (2001).

[64] Armstrong, E., Tallavaara, M., Hopcroft, P. O. & Valdes, P. J. North African humid periods over the past 800,000 years. *Nat. Commun.* **14**, 5549 (2023). 10.1038/s41467-023-41219-4 PMC10491769 37684244

[65] Berger, A. Long-term variations of daily insolation and Quaternary climatic changes. *J. Atmos. Sci.* **35**, 2362–2367 (1978).

[66] Berger, A., Loutre, M. F. & Gallée, H. Sensitivity of the LLN climate model to the astronomical and CO 2 forcings over the last 200 ky. *Clim. Dyn.* **14**, 615–629 (1998).

[67] Loulergue, L. et al. Orbital and millennial-scale features of atmospheric CH4 over the past 800,000 years. *Nature* **453**, 383–386 (2008). 10.1038/nature06950 18480822

[68] Spahni, R. et al. Atmospheric methane and nitrous oxide of the late Pleistocene from Antarctic ice cores. *Science* **310**, 1317–1321 (2005). 10.1126/science.1120132 16311333

[69] Schneider, U., Hänsel, S., Finger, P., Rustemeier, E. & Ziese, M. GPCC climatology version 2022 at 2.5^°^: Monthly land-surface precipitation climatology for every month and the total year from rain-gauges built on GTS-based and historical data. https://opendata.dwd.de/climate_environment/GPCC/html/gpcc_normals_v2022_doi_download.html. Artwork Size: min. 200 KByte - max. 10 MByte per gzip archive Pages: min. 200 KByte - max. 10 MByte per gzip archive (2022).

[70] Wang, B., Liu, J., Kim, H.-J., Webster, P. J. & Yim, S.-Y. Recent change of the global monsoon precipitation (1979–2008). *Clim. Dyn.* **39**, 1123–1135 (2012).

[71] Bartlein, P. J. & Shafer, S. L. Paleo calendar-effect adjustments in time-slice and transient climate-model simulations (PaleoCalAdjust v1.0): impact and strategies for data analysis. *Geosci. Model Dev.* **12**, 3889–3913 (2019).

[72] Herbert, T. D., Peterson, L. C., Lawrence, K. T. & Liu, Z. Tropical ocean temperatures over the past 3.5 million years. *Science* **328**, 1530–1534 (2010). 10.1126/science.1185435 20558711

[73] Proceedings of the Ocean Drilling Program, 121 scientific results (1991). http://www-odp.tamu.edu/publications/121_SR/121TOC.HTM (1991).

[74] Clemens, S. C. et al. Remote and local drivers of pleistocene south asian summer monsoon precipitation: a test for future predictions. *Sci. Adv.* **7**, eabg3848 (2021). 10.1126/sciadv.abg3848 PMC8177704 34088672

[75] Tarique, M. et al. Enhanced CO2 degassing from the tropical indian ocean during cold climatic events of the last glacial cycle. *Paleoceanogr. Paleoclimatol.* **38**, e2022PA004570 (2023).

[76] Govil, P. & Divakar Naidu, P. Variations of indian monsoon precipitation during the last 32kyr reflected in the surface hydrography of the western Bay of Bengal. *Quat. Sci. Rev.* **30**, 3871–3879 (2011).

[77] Met Office. *Cartopy: a cartographic Python library with a Matplotlib interface*. Exeter, Devon https://cartopy.readthedocs.io (2010–2015).

