# Author Correction: Asian summer monsoon orbital variability directly paced by CO2 and precession, not eccentricity

**DOI:** 10.1038/s41467-026-78966-z

**Published:** 2026-11-04

**Authors:** Jeanne Millot-Weil, Paul J. Valdes, Alexander Farnsworth

**Affiliations:** 1 School of Geographical Sciences, University of Bristol, Bristol, UK; 2 State Key Laboratory of Tibetan Plateau Earth System, Environment and Resources, Institute of Tibetan Plateau Research, Chinese Academy of Sciences, Beijing, 100101 China

**Keywords:** Palaeoclimate, Climate and Earth system modelling

Correction to: *Nature Communications* https://doi.org/10.1038/s41467-026-76856-y, published online 22 August 2026

In the version of this article initially published, there were errors in the values presented in the upper panel of Fig. 4 and associated text. In the fifth paragraph of the “Disentangling each driver’s impacts on Monsoon Variability” section, in the text now reading “For precipitation, both greenhouse gas and ice-sheet forcing produce a drying tendency relative to the Orb simulation, although to a lesser magnitude of drying and does not reach the mean precipitation level of the All_forcings simulations (5.4 mm/day for Orb, 5.1 mm/day for Orb_Ice, 5.0 mm/day for Orb_Ghg). The forcing set that most closely matches the mean monsoon period precipitation in All_forcing (4.8 mm/day for All) is Orb_Ghg,” the values 5.4, 5.1, 5.0 and 4.8 mm/day replace the original 28.9, 28.7, 28.0 and 27.9, respectively. The figure and text are amended in the HTML and PDF versions of the article. For comparison, the original figure is available as Supplementary Information accompanying this amendment.

## Supplementary information

Original, uncorrected Fig. 4

