# Supplementary material for: Asian summer monsoon orbital variability directly paced by CO2 and precession, not eccentricity
Source: Nat Commun. 2026 Aug 22;17:10067. doi: 10.1038/s41467-026-76856-y (PMC13594180; doi:10.1038/s41467-026-76856-y)
Supplement: Supplementary file 1 [file 41467_2026_76856_MOESM1_ESM.pdf]

Supplementary information Asian summer monsoon orbital variability directly paced by CO2 and precession, not eccentricity

Authors: Jeanne Millot-Weil[1]*, Paul J. Valdes[1,2], Alexander Farnsworth[1,2]

[1]School of Geographical Sciences, University of Bristol, Bristol, United Kingdom, [2] Institute of Tibetan Plateau Research (ITP), Chinese Academy of Sciences, Beijing, China

*Corresponding author: Jeanne Millot-Weil (xk22684@bristol.ac.uk)

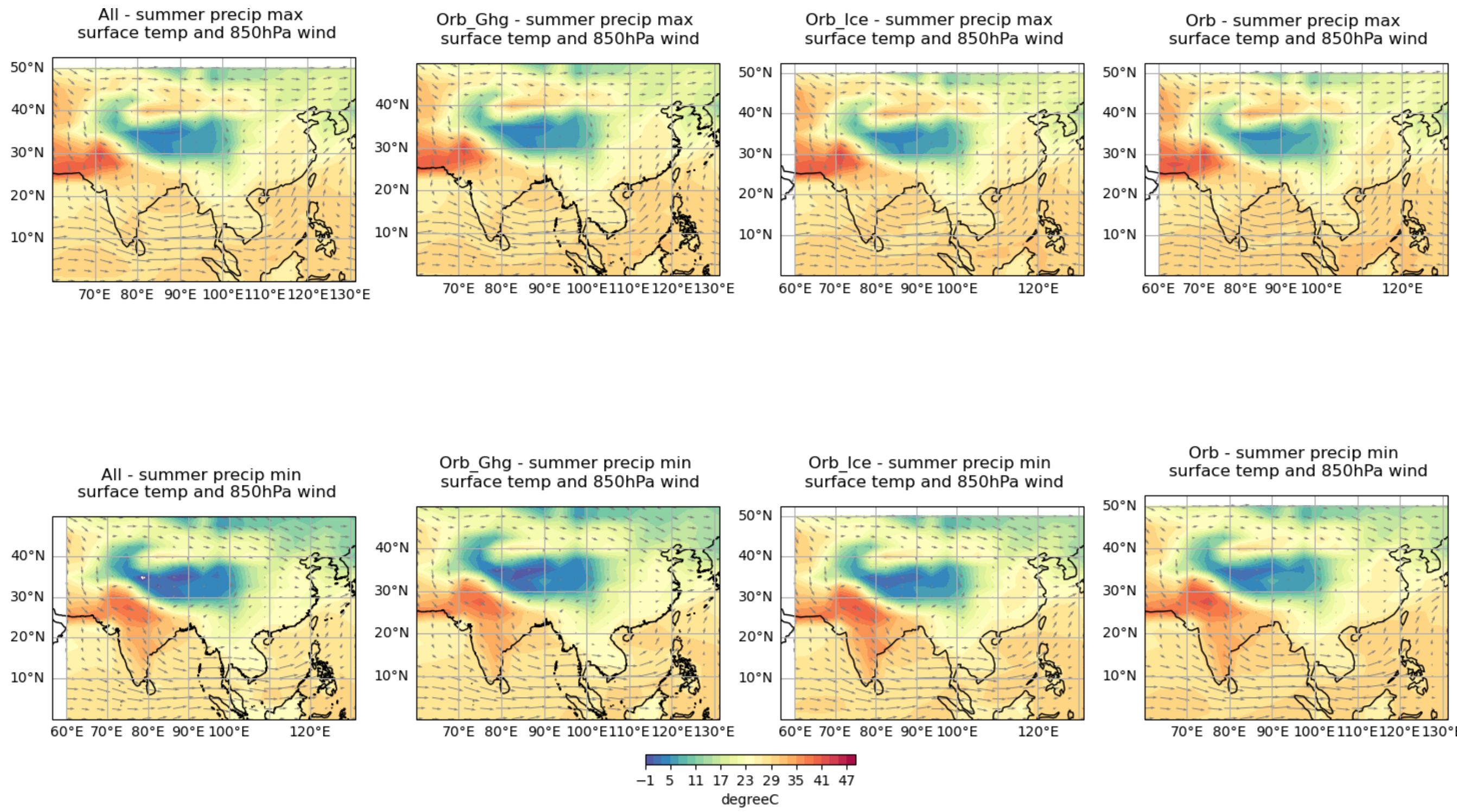

Supplementary Figure 1: **Average surface air temperature (°C) and 850hPa wind (m/s) during strong and weak monsoon periods over the last 800-Kyr for each forcing experiment**. Top (bottom) row indicates periods of strengthened (weakened) summer monsoons activity which correspond to the mean of snapshots for May-September MJJAS precipitation that exceeds the third quartile (below the first quartile). Quantiles are calculated using summer precipitation from every snapshots. Average surface temperature for minimum monsoon are : $\text{Tmin}_{All} = 22.6^o$, $\text{Tmin}_{Orb\_Ghg} = 22.9^o$, $\text{Tmin}_{Orb\_Ice} = 24.1^o$, $\text{Tmin}_{Orb} = 24.5^o$. Average surface temperature for max monsoon are : $\text{Tmax}_{All} = 24.9^o$, $\text{Tmax}_{Orb\_Ghg} = 24.9^o$, $\text{Tmax}_{Orb\_Ice} = 25.5^o$, $\text{Tmax}_{Orb} = 25.8^o$. Maps created using Cartopy [1].

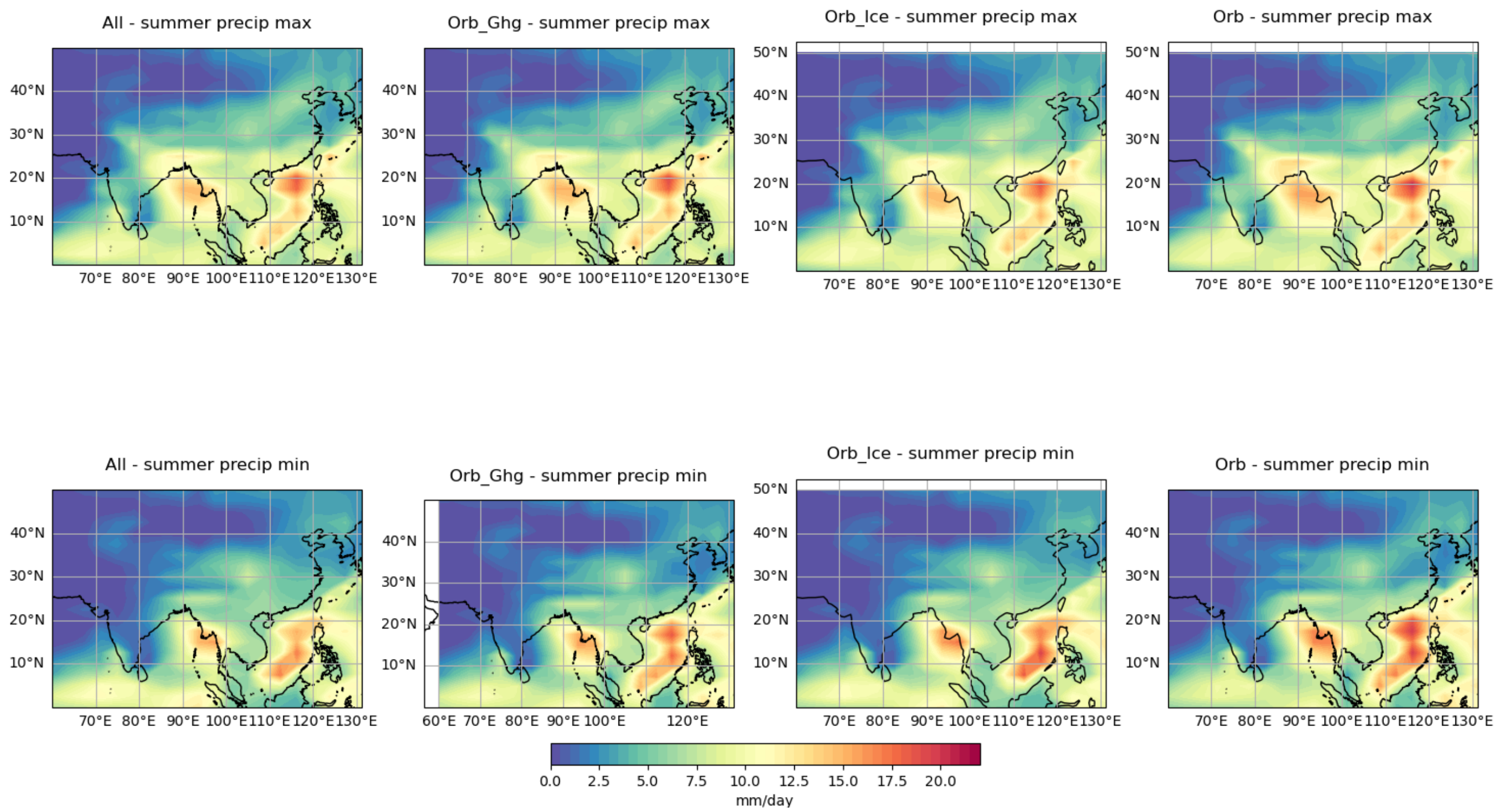

Supplementary Figure 2: **Average precipitation (mm/day) during strong and weak monsoon periods over the last 800 Kyr for each forcing experiment**. Top (bottom) row indicates periods of strengthened (weakened) summer monsoons activity which correspond to the mean of snapshots for May-September (MJJAS) precipitation that exceeds the third quartile (below the first quartile). Quantiles are calculated using summer precipitation from every snapshots. Maps created using Cartopy [1].

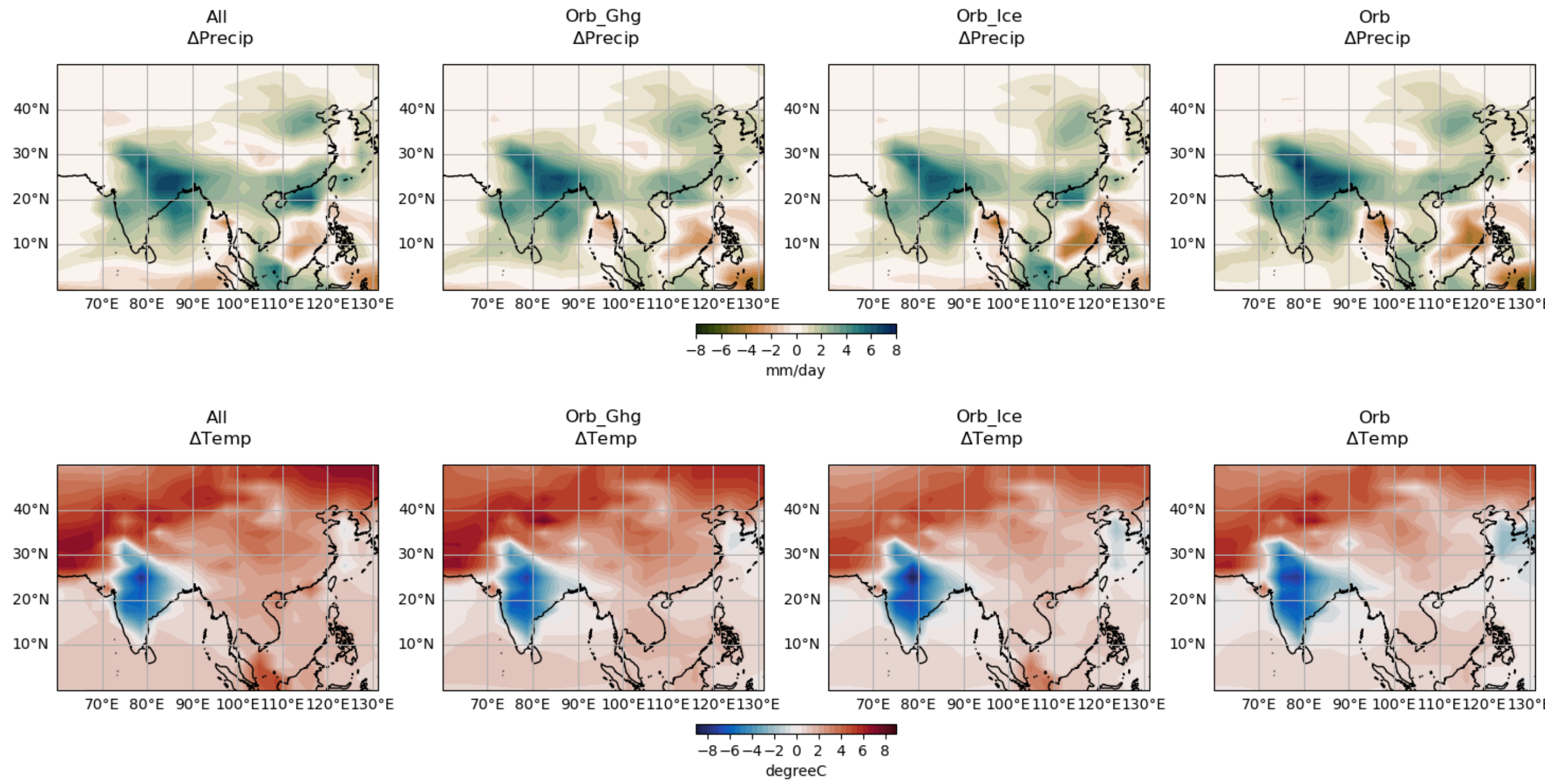

Supplementary Figure 3: **Precipitation and surface air temperature anomaly between stronger and weaker summer monsoons periods over the last 800 Kyr for each forcing experiment**. Top row indicates precipitation anomaly (mm/day) while the bottom row indicates surface air temperature (°C) anomalies as the difference between stronger and weaker summer monsoon activity which correspond to the mean of snapshots for May-September (MJJAS) precipitation that exceeds the third quartile (below the first quartile). Quantiles are calculated using all snapshot summer precipitation. Average ΔTemperature are : $\Delta T_{All} = 2.3^o$, $\Delta T_{Orb\_Ghg} = 2.0^o$, $\Delta T_{Orb\_Ice} = 1.4^o$, $\Delta T_{Orb} = 1.3^o$. Average ΔPrecipitation are : $\Delta Precip_{All} = 0.78$ mm/day, $\Delta Precip_{Orb\_Ghg} = 0.75$ mm/day, $\Delta Precip_{Orb\_Ice} = 0.73$ mm/day, $\Delta Precip_{Orb} = 0.71$ mm/day. Maps created using Cartopy [1].

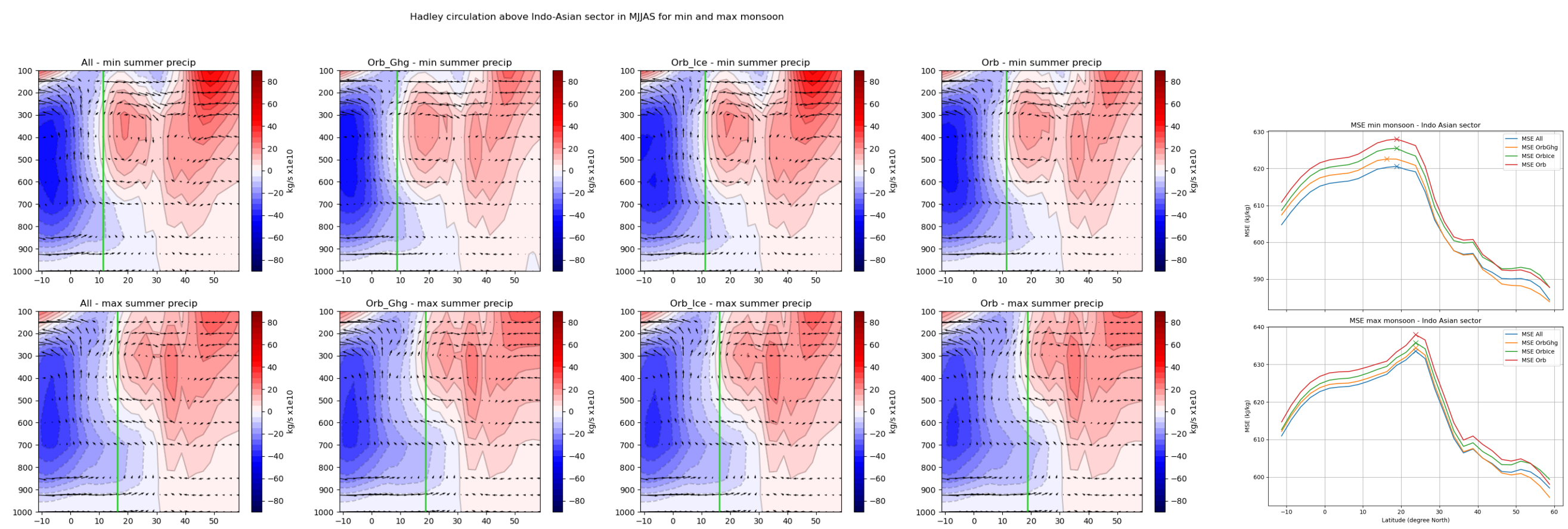

(a) (b)

Supplementary Figure 4: **(a) Atmosphere streamfunction and vertical wind during strong and weak monsoon periods over the last 800 Kyr for each forcing experiment**. Stronger (weaker) summer monsoons averaged between 60^oE and 101^oE (Indian sector) correspond to the mean of snapshots for May-September (MJJAS) precipitation that exceeds the third quartile (below the first quartile). Quantiles are calculated using all snapshot summer precipitation. The vertical green line corresponds to where the vertical average streamfunction between 700hPa and 300hPa is equal to 0. (b) Subcloud moist static energy (MSE) for the minimal summer monsoon cases (top) and maximal summer monsoon (bottom) for each simulation set. MSE is shown for the month of peak MSE in each case. Crosses mark the locations of maximum MSE.

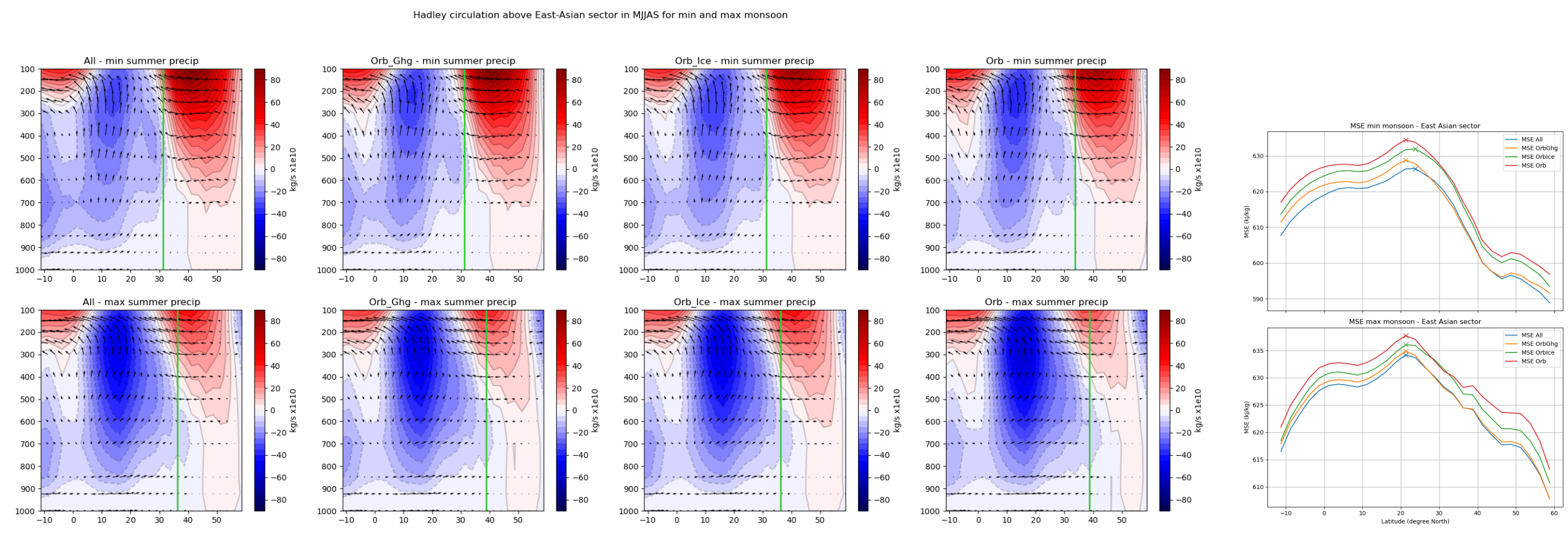

(a) (b)

Supplementary Figure 5: **(a) Atmosphere stream function and vertical wind during strong and weak monsoon periods over the last 800 Kyr for each forcing experiment**. Stronger (weaker) summer monsoons averaged between 101°E and 120°E (East Asian sector) correspond to the mean of snapshots for May-September (MJJAS) precipitation that exceeds the third quartile (below the first quartile). Quantiles are calculated using all snapshot summer precipitation. The vertical green line corresponds to where the vertical average streamfunction between 700hPa and 300hPa is equal to 0. (b) Subcloud moist static energy (MSE) for the minimal summer monsoon cases (top) and maximal summer monsoon (bottom) for each simulation set. MSE is shown for the month of peak MSE in each case. Crosses mark the locations of maximum MSE.

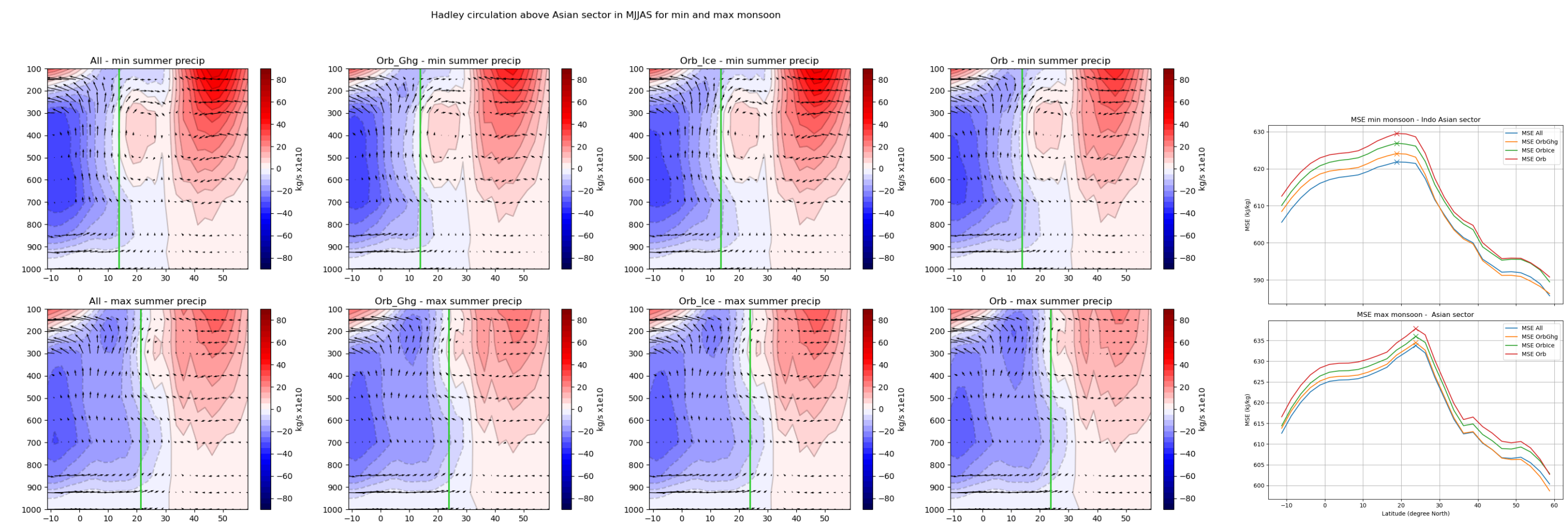

(a) (b)

Supplementary Figure 6: **(a) Atmosphere stream function and vertical wind during strong and weak monsoon periods over the last 800 Kyr for each forcing experiment**. Stronger (weaker) summer monsoons averaged between 60^oE and 120^oE (broad Asian sector) correspond to the mean of snapshots having MJJAS precipitation exceeds the third quartile (below the first quartile). Quantiles are calculated using all snapshot summer precipitation. The vertical green line corresponds to where the vertical average streamfunction between 700hPa and 300hPa is equal to 0. (b) Subcloud moist static energy (MSE) for the minimal summer monsoon cases (top) and maximal summer monsoon (bottom) for each simulation set. MSE is shown for the month of peak MSE in each case. Crosses mark the locations of maximum MSE.

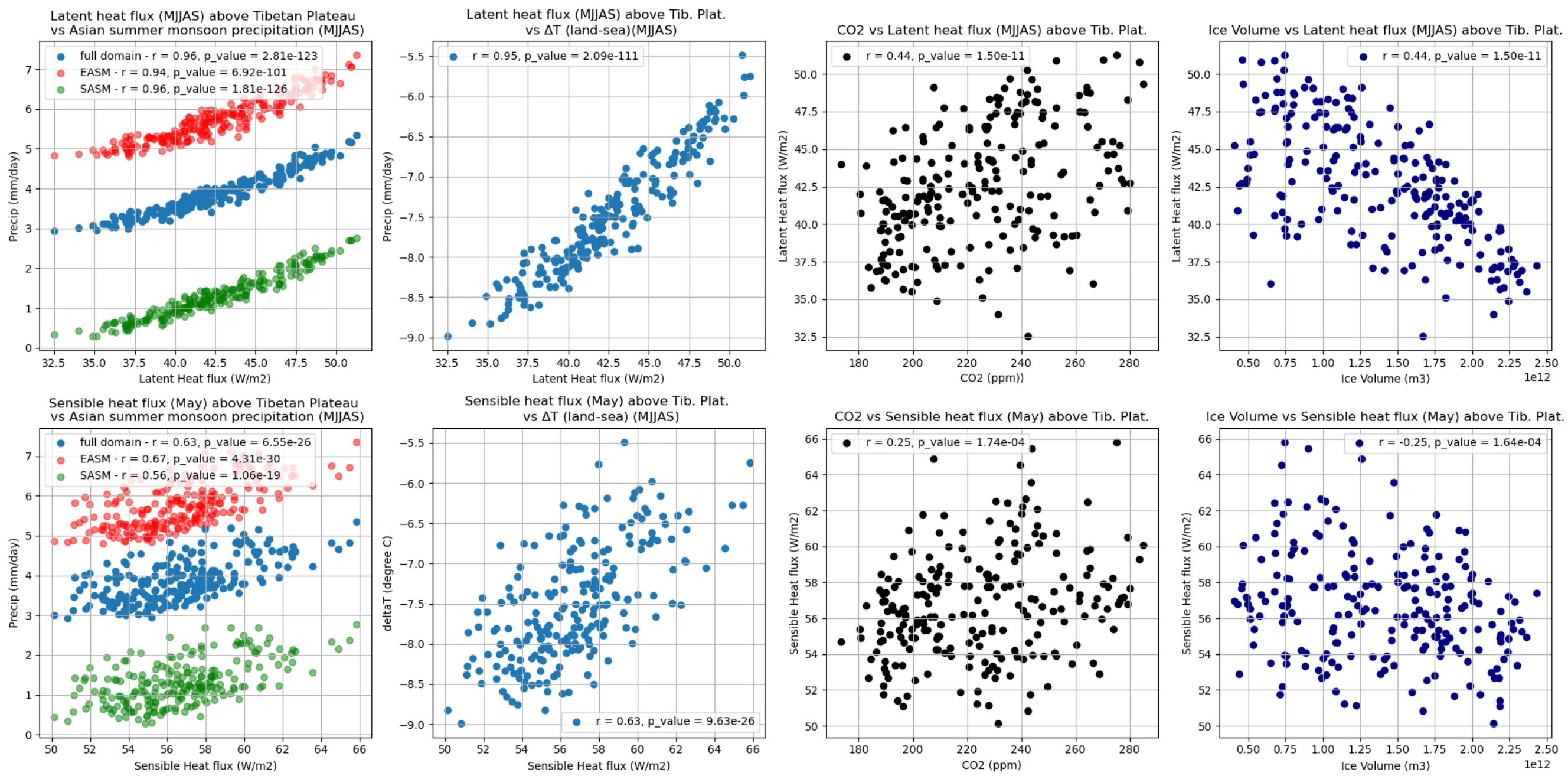

Supplementary Figure 7: **Relationships between surface heat fluxes and Asian summer monsoon in the All_forcings simulations.** The first (second) row shows relationships with latent (sensible) heat flux over the Tibetan Plateau (25–45°N, 65–110°E), calculated in MJJAS (May). From left to right: relationships with (i) Asian summer monsoon precipitation over the full study region (blue shaded area; Fig.(1) main text), East Asia (EASM; 90–130°E, red), and South Asia (SASM; 60–90°E, green), (ii) land–sea thermal contrast (MJJAS; Fig.(1) main text), (iii) atmospheric CO_2 forcing, and (iv) prescribed ice sheet volume. Pearson correlation coefficients (r) and associated p-values (95% confidence level) are indicated for each relationship.

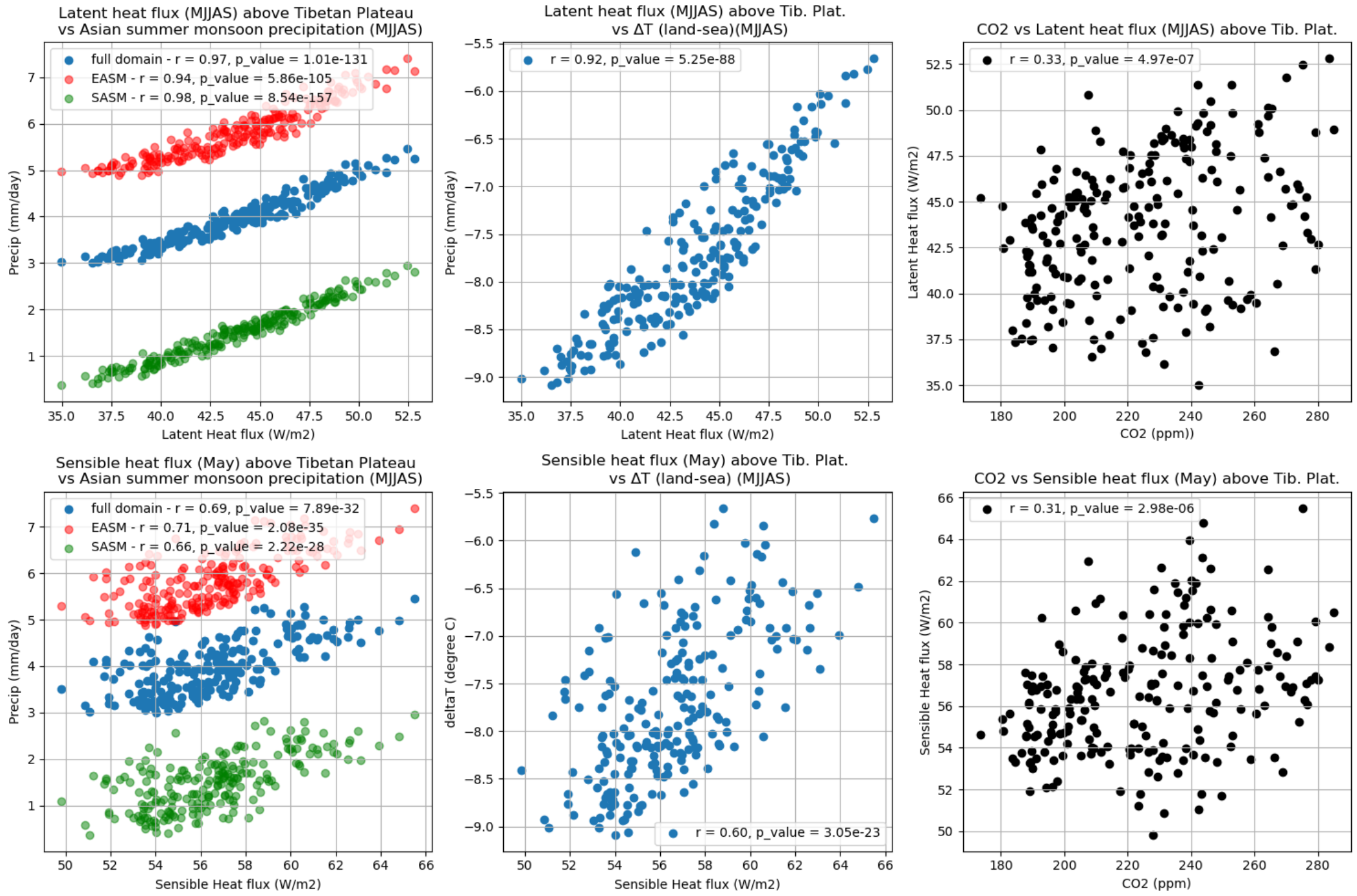

Supplementary Figure 8: **Relationships between surface heat fluxes and Asian summer monsoon in the OrbGhg simulations.** The first (second) row shows relationships with latent (sensible) heat flux over the Tibetan Plateau (25–45°N, 65–110°E), calculated in MJJAS (May). From left to right: relationships with (i) Asian summer monsoon precipitation over the full study region (blue shaded area; Fig.(1) main text), East Asia (EASM; 90–130°E, red), and South Asia (SASM; 60–90°E, green), (ii) land–sea thermal contrast (MJJAS; Fig.(1) main text) and (iii) atmospheric CO_2 forcing. Pearson correlation coefficients (r) and associated p-values (95% confidence level) are indicated for each relationship.

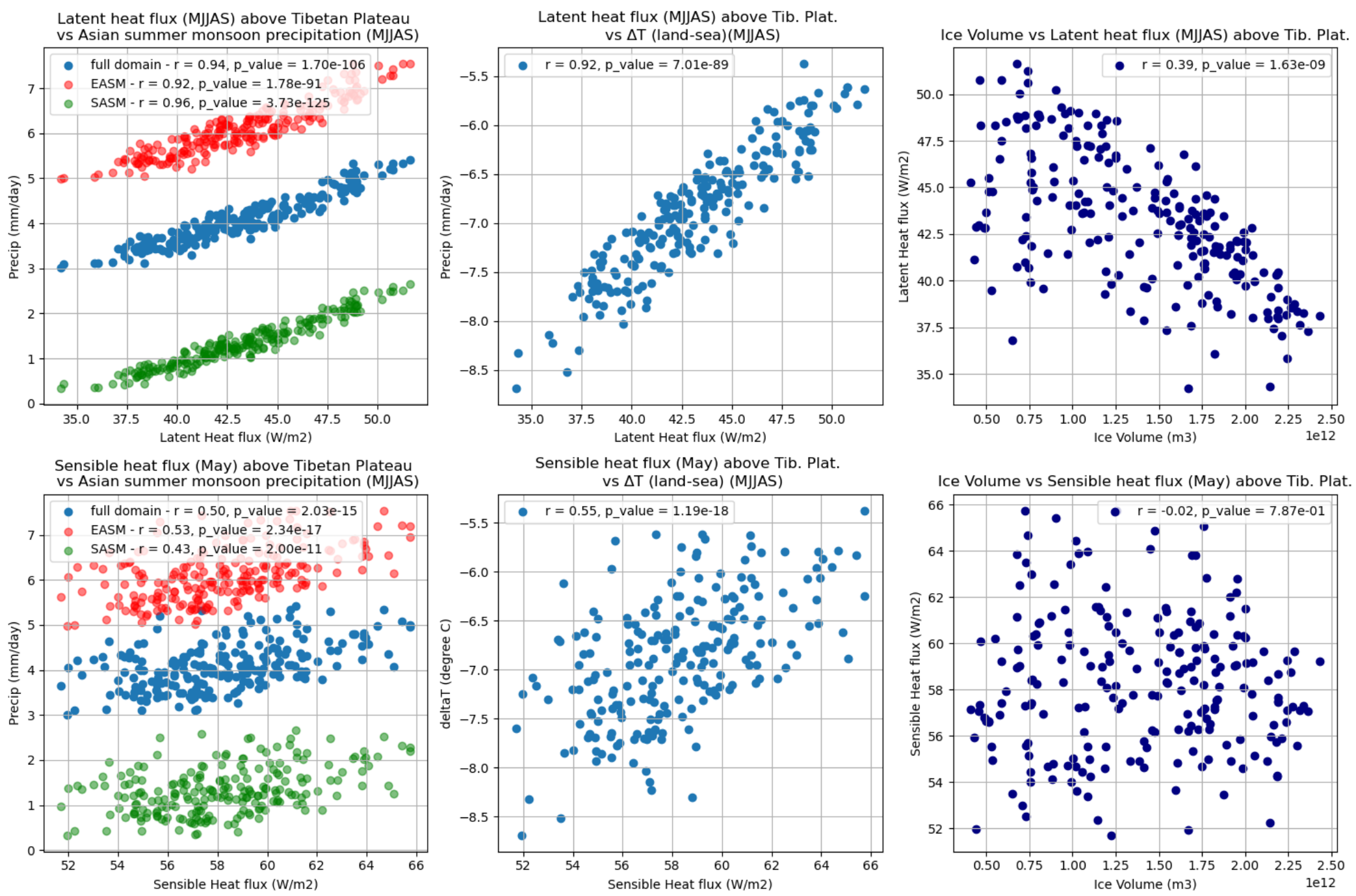

Supplementary Figure 9: **Relationships between surface heat fluxes and Asian summer monsoon in the OrbIce simulations.** The first (second) row shows relationships with latent (sensible) heat flux over the Tibetan Plateau (25–45°N, 65–110°E), calculated in MJJAS (May). From left to right: relationships with (i) Asian summer monsoon precipitation over the full study region (blue shaded area; Fig.(1) main text), East Asia (EASM; 90–130°E, red), and South Asia (SASM; 60–90°E, green), (ii) land–sea thermal contrast (MJJAS; Fig.(1) main text) and (iii) prescribed ice sheet volume. Pearson correlation coefficients (r) and associated p-values (95% confidence level) are indicated for each relationship.

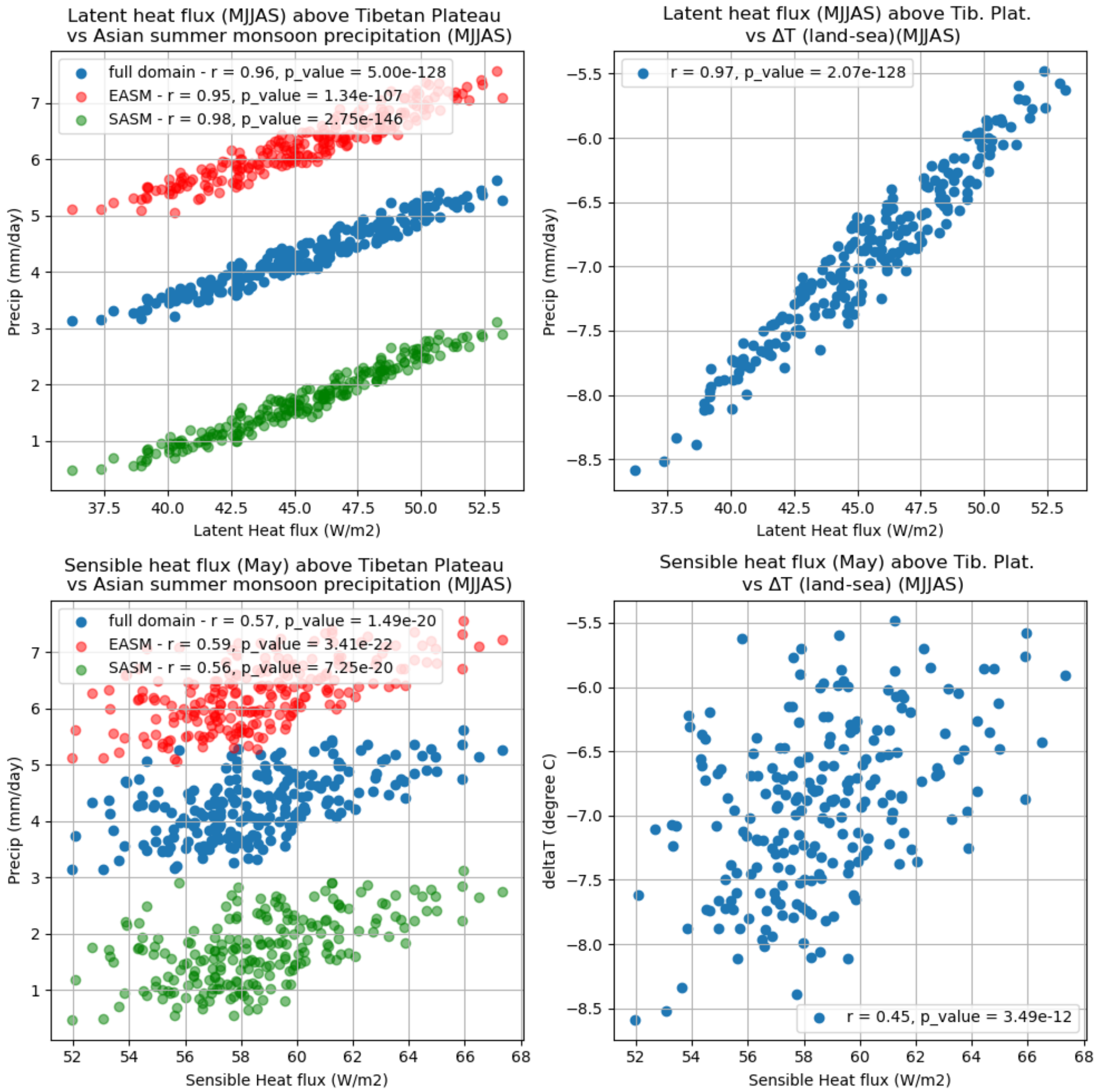

Supplementary Figure 10: **Relationships between surface heat fluxes and Asian summer monsoon in the Orb simulations.** The first (second) row shows relationships with latent (sensible) heat flux over the Tibetan Plateau (25–45°N, 65–110°E), calculated in MJJAS (May). From left to right: relationships with (i) Asian summer monsoon precipitation over the full study region (blue shaded area; Fig.(1) main text), East Asia (EASM; 90–130°E, red), and South Asia (SASM; 60–90°E, green) and (ii) land–sea thermal contrast (MJJAS; Fig.(1) main text). Pearson correlation coefficients (r) and associated p-values (95% confidence level) are indicated for each relationship.

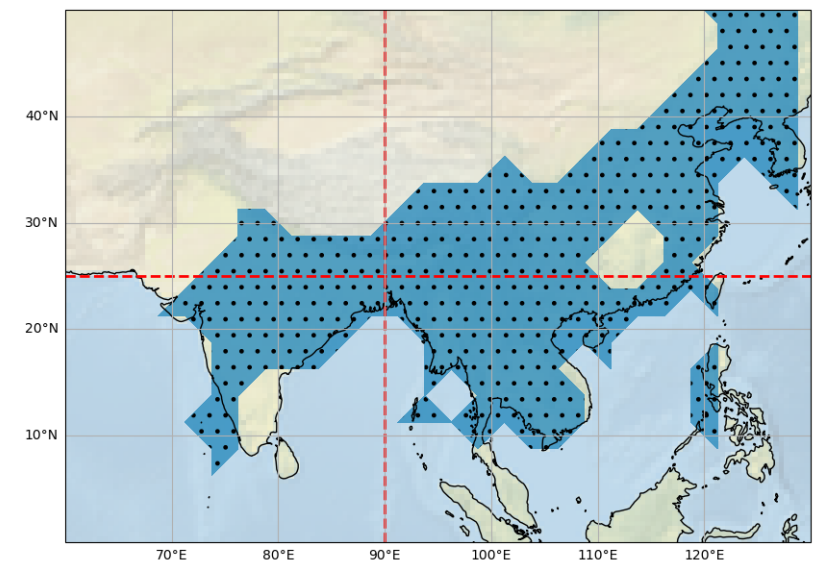

(a)

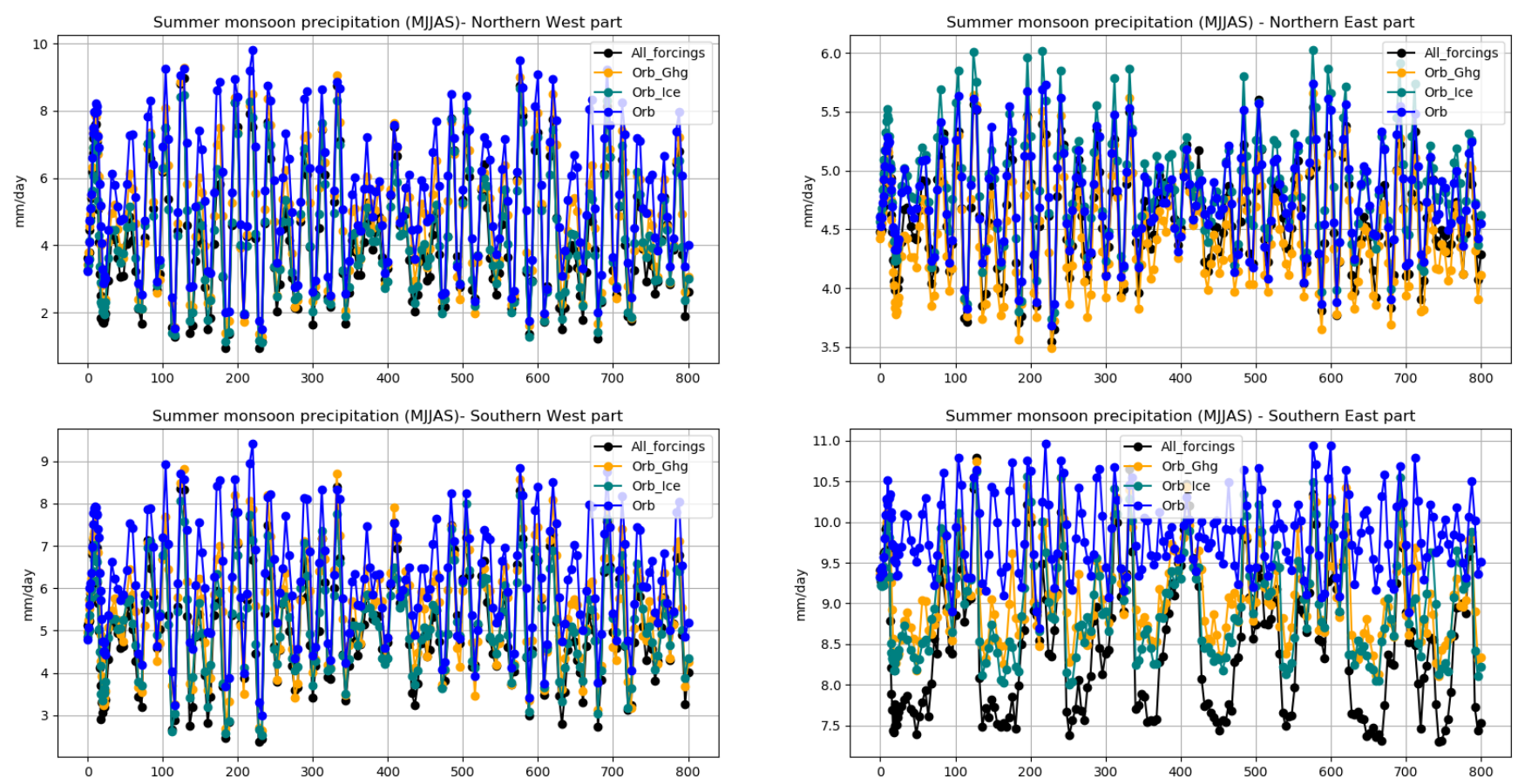

(b)

Supplementary Figure 11: **Quadrant precipitation timeseries over the last 800 Kyr for each forcing experiment**. (a) Asian summer monsoon region (see Methods), red stippled line separates regions of interest for timeseries. (b) Four forcing experiments (All in black, Orb_Ghg in green, Orb_Ice in purple and Orb in blue) precipitation timeseries (mm/day) for each quadrant shown in (a) averaged over May to September. Maps created using Cartopy [1].

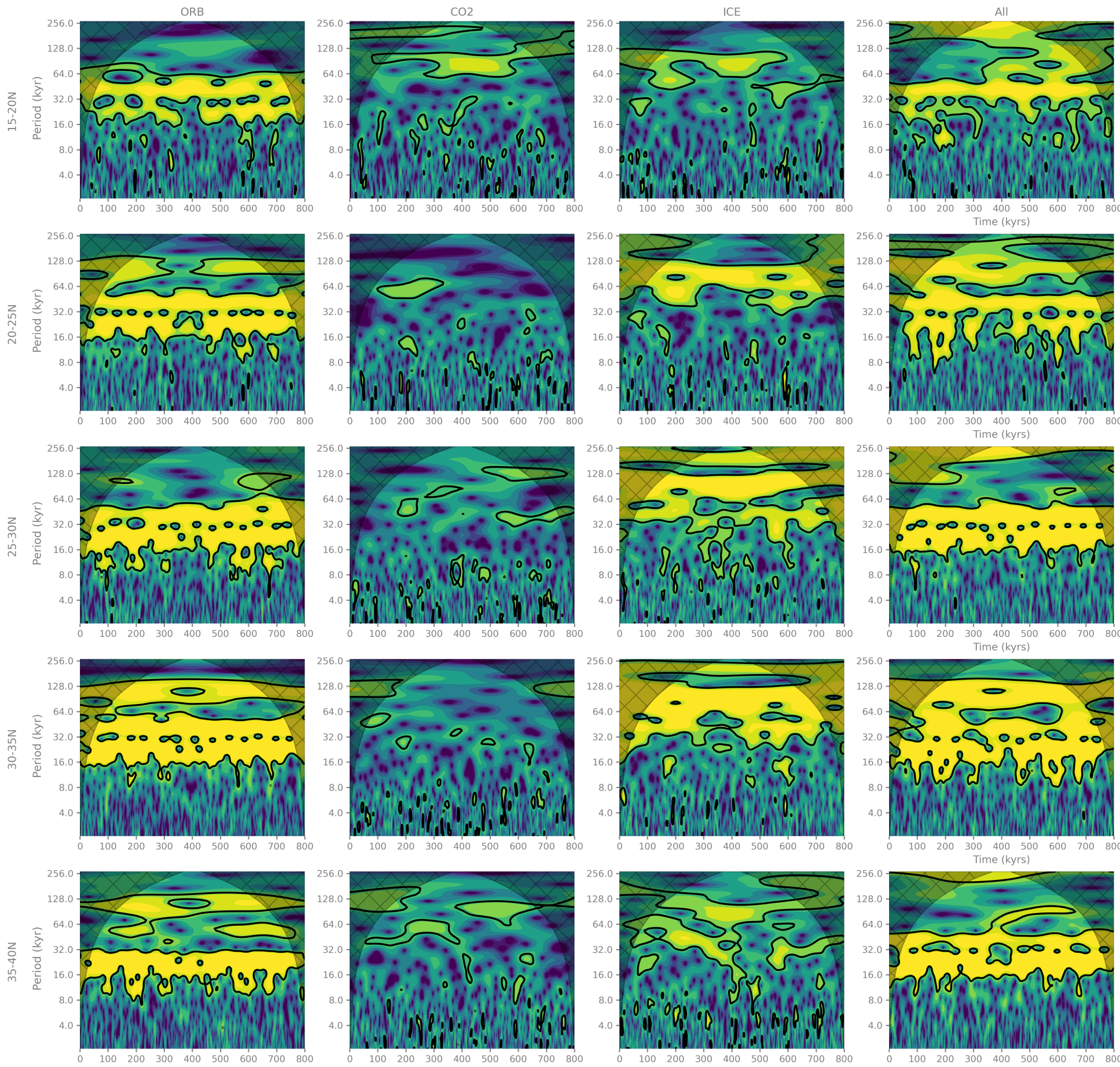

Supplementary Figure 12: **Wavelet Power Spectra for timeseries from each simulation set between (15°-40°N, 105°/120°E)**. Thick black lines contour depicts regions at the 95% confidence level relative to a red-noise process. Shaded and hatched regions denote the cone of influence of the mother wavelet. The first, second and third column depict the transient simulations from [2]; ORB, CO2, ICE and All respectively. Rows show, from top to bottom, results for 15°-20°N, 20°-25°N, 25°-30°N, 30°-35°N and 35°-40°N latitudinal bands.

References

1. Met Office. *Cartopy: a cartographic Python library with a Matplotlib interface* (Exeter, Devon, 2010 - 2015). `https://cartopy.readthedocs.io`.
2. Su, B., Sun, Y. & Zhou, M. Orbital-accelerated transient simulations of glacial-interglacial climate cycles for the last 800,000 years. *Scientific Data* **12,** 961. ISSN: 2052-4463. `https://www.nature.com/articles/s41597-025-05297-x` (June 2025).
