# Supplementary material for: Asian summer monsoon orbital variability directly paced by CO2 and precession, not eccentricity
Source: Nat Commun. 2026 Aug 22;17:10067. doi: 10.1038/s41467-026-76856-y (PMC13594180; doi:10.1038/s41467-026-76856-y)
Supplement: Supplementary file 2 [file 41467_2026_76856_MOESM2_ESM.pdf]

nature portfolio

Peer Review File

Asian summer monsoon orbital variability directly paced by CO2 and precession, not eccentricity.

Corresponding Author: Ms Jeanne Millot-Weil

This file contains all reviewer reports in order by version, followed by all author rebuttals in order by version.

Version 0:

Reviewer comments:

Reviewer #1

(Remarks to the Author)
The evolution of Asian summer monsoon since the mid-Pleistocene transition (~700 ka) has long been debated over the past few decades, with loess, lake, and marine records showing a strong eccentricity cycle (100 kyr) while the speleothem δ18O records showing a strong precession cycle (200 kyr), which is known as "Chinese 100 kyr problem" (Cheng et al., 2021). Also see Li et al. (2024) for an introduction.

The paper by Millot-Weil et al. investigates the Asian summer monsoon over the past 800 ka using climate simulation at 219 snapshots performed with the HadCM3 model, and they concluded that Asian summer monsoon variations are predominantly driven by the direct influence of precession and greenhouse gas changes, and the role of eccentricity and ice sheet variations are neglectable, which only impact the monsoon amplitude (monsoon intensity), but not its pace.

I am not an expert on climate model simulation. But I find several mistakes in this paper about the basic concept of monsoon definition, which may affect the conclusion of this paper. Also, there are serious problems in the writing of this paper, which are not in accordance with academic ethic. For instance, several proxy records are mentioned and presented in this paper, but the original references are not properly cited. Moreover, there are many technical errors in this paper.

Since the analysis methods their conclusion relies on are questionable, I cannot judge whether the conclusion is reliable.

Details are as follow:
1. The Asian monsoon includes both summer monsoon and winter monsoon. This paper only investigates the summer monsoon. The author should make it clear in the title and other area in the manuscript.

2. Definition of monsoon domain. The author used the region (lat: 0-50 N, lon: 40-130 E, in their Fig. 2-3) to represent the Asian summer monsoon domain. This is incorrect, because this region also includes the westerly region (arid region) and the equatorial region, which are not Asian summer monsoon region.

The summer monsoon climate is characterized by rainy summer and dry winter, and the accompanied reversal of wind direction between the summer and winter (Wang & Ding, 2008). The definition of summer monsoon domain has been clearly shown in previous studies. For instance, the global monsoon domain is defined as the region where local summer-minus-winter precipitation rate exceeding 2.0 mm/day and the local summer precipitation exceeding 55% of the annual total (Wang et al., 2012). The local summer is from May to September (MJJAS) for Northern Hemisphere and from November to March (NDJFM) for Southern Hemisphere.

In the Method section, the author should follow these studies to clearly define the monsoon region where they use to calculate the Asian summer monsoon rainfall change. After this change, the author should re-perform all calculations. This change may affect the conclusion of this paper and the author should revise their manuscript accordingly. Since Asian summer monsoon rainfall is mainly occur on land, I suggest the author use the Global Precipitation Climatology Centre (GPCC) rainfall data to define the Asian summer monsoon region. The GPCC climatology data can be found here: (https://opendata.dwd.de/climate_environment/GPCC/html/gpcc_normals_v2022_doi_download.html)

3. The summer season is not clearly defined. It seems that the author used June-to-September for calculation, but they

showed JJS in Fig. 2, what this means? Just misspell? I suggest them to analyze both JJA (months 6-7), MJJAS (months 5-9), and annual rainfall, how the different?

4. Many proxy records were not properly cited. Especially, the original references were not cited. For instance, the Hulu/Sanbao speleothem δ18O records were presented in Fig. 2, but without citation and the original reference were not listed. Note that the review paper by Sun et al. (2022) cannot replace the original reference.

5. In the Introduction section, the review of literature is inappropriate and can not truly reflect the opinion of the original references. First, the author may not read the original references carefully and proxy records from different time periods were discussed and compared together, which is not objective, leading to artificial bias. For instance, the reference of Sun et al. (2006) indicated that “Spectral analyses of the stacked monsoon proxies indicate that characteristics of both the summer and winter monsoons are dominated mainly by variance in the eccentricity (410- and 100-kyr), obliquity (41-kyr) and precession (23- and 19-kyr) bands over the past 3.4 Myr”. However, Millot-Weil et al. stated that “loess deposits and stable oxygen isotopes from Chinese speleothem are dominated by obliquity (≈40 kyr) and precession pacing (≈20 kyr)”.

6. The format of references is wrong and unprofessional.

7. The method used to assess model’s ability to simulate accurately the Asian monsoon system is not correct. The model’s ability to simulate modern monsoon climate should be comparing the model results at modern time with modern observations, including rainfall distribution, wind field, and so on.

8. Line 88: δ18O records from marine cores and speleothem are not a temperature proxy.
Planktonic foraminifera δ18O records are a proxy for salinity, and Benthic foraminifera δ18O records are a proxy for deep sea temperature and global ice volume. Sea surface temperature (SST) has been reconstructed by Mg/Ca ratio in many studies, and they are the one should be compared with model results of SST. There are also many Land temperature records (Lu et al., 2019, 2022), which can be compared with land surface temperature (LST) from model results.

9. Line 115-117: “for the strongest monsoons, the Orb set is overall cooler than the two other sets”, cooler? Are you sure? The same for the weak monsoons.

10. What JJS on Fig. S1 means? JJAS?

11. “geographic repartition” in the paper is inappropriate, “spatial distribution” should be more appropriate?

12. Line 191: East Asian region (15-20∘N) is not Northern portion, but Southern portion.

13. The edge effects might distort the result of wavelet power spectrum analysis, and the cone of influence (COI) should be performed and shown (Grinsted et al., 2004).

14. The model data used for analysis is essential for repeatability and verifiability of this study, as well as for comparison and dispersing (citation) in the research community, the model data should be stored on public data website like Zenodo or Figshare. A Data availability statement is also needed to comply with the journal policy.

The literature I mention in the comments:
Cheng, H. et al. Orbital-scale Asian summer monsoon variations: Paradox and exploration. Science China Earth Sciences 64, 529-544 (2021).
Li, X. et al. Loess deposits in the low latitudes of East Asia reveal the ~20-kyr precipitation cycle. Nature Communications 15, 1023 (2024).
Wang, B. & Ding, Q. Global monsoon: Dominant mode of annual variation in the tropics. Dynamics of Atmospheres and Oceans 44, 165-183 (2008).
Wang, B., Liu, J., Kim, H.-J., Webster, P. J. & Yim, S.-Y. Recent change of the global monsoon precipitation (1979–2008). Climate Dynamics 39, 1123-1135 (2012).
Sun, Y. et al. A review of orbital-scale monsoon variability and dynamics in East Asia during the Quaternary. Quaternary Science Reviews 288, 107593 (2022).
Sun, Y., Clemens, S. C., An, Z. & Yu, Z. Astronomical timescale and palaeoclimatic implication of stacked 3.6-Myr monsoon records from the Chinese Loess Plateau. Quaternary Science Reviews 25, 33-48 (2006).
Grinsted, A., Moore, J. C. & Jevrejeva, S. Application of the cross wavelet transform and wavelet coherence to geophysical time series. Nonlinear processes in geophysics 11, 561-566 (2004).
Lu, H. et al. 800-kyr land temperature variations modulated by vegetation changes on Chinese Loess Plateau. Nature Communications 10, 1958 (2019).
Lu, H. et al. Decoupled Land and Ocean Temperature Trends in the Early-Middle Pleistocene. Geophysical Research Letters 49, e2022GL099520 (2022).

Reviewer #2

(Remarks to the Author)
Summary
Millot-Weil et al. used three sets of HadCM3 simulations to disentangle the impacts of orbital forcing, greenhouse gases, and ice sheets on Asian monsoon pacing over the last 800,000 years. They are particularly interested in better understanding the controlling processes that lead to large variations in monsoon pacing on longer timescales, which has been long debated in the literature with differing perspectives from modeling and proxy-based studies. The authors found that precession primarily modulates monsoon variability (temperature and precipitation), confirming findings of previous studies. Greenhouse gases impose a ~100 kyr signal on SST variability associated with the monsoon. Including ice sheet variations dampens the impacts of precession and GHG variations, but does not alter the monsoon's pace. The simulation set is uniquely designed to provide a valuable contribution to our understanding of Asian monsoon variability in the past and reveals some new information about Asian monsoon pacing over long timescales. The reported results are reasonably supported by the analysis and simulations. However, I do have a few major concerns with the current scope of the paper that I will describe in more detail below.

Minor comments

- Line 23 should be speleothems (?)
- Line 43 should be ice sheets
- Line 73 perhaps switch "orbits" to "orbital cycles"
- Line 202 should be "proxy-model comparison"
- Line 222 "an other" should be "another"
- Figure 2, Figure S1, Figure S2: Why is N Hemisphere summer defined as JJS or June-July-September, omitting August? Is this is a typo (should be JJAS)?

Major comments

- Lines 31 to 34 (also stated on Lines 77-79): the traditional view of monsoonal circulation as a large-scale sea breeze driven by land-sea contrast has been challenged by theories that characterize monsoonal circulation with variations in the Intertropical Convergence Zone and moist static energy (e.g., Gagdil 2018, https://doi.org/10.1007/s12040-017-0916-x; Geen et al., 2020, https://doi.org/10.1029/2020RG000700). Do your simulations suggest that the traditional perspective is correct? It would be helpful to acknowledge these other theories in your introduction and then again acknowledge these theories in Section 2.1 in the context of describing Figure 2.
- Section 2.2: I appreciate that you disentangle the monsoon pacing associated with orbital, greenhouse gas, and ice variations. These are interesting results. However, I am curious about the climate dynamics associated with the introduction of greenhouse gas and ice sheet forcing, which can be addressed by the simulations. For example, why does the introduction of varying greenhouse gases make eccentricity scale-variations in SSTs apparent? Also, is the mechanism that causes ice sheets to influence monsoon intensity in your simulations similar to previous studies (e.g., citations on Lines 175-177) and why does this mechanism not impact pacing?
- When you aggregate model output by N Hemisphere summer (likely JJAS, see minor comment above), how do you account for the impact of orbital forcing on the varying length of fixed-angular months over time (i.e., the paleo-calendar effect, e.g., Bartlein & Shafer, 2019, https://doi.org/10.5194/gmd-12-3889-2019)? If you are using fixed-length monthly output from the simulation based on a modern calendar, your results include the effects of orbital variations on insolation and the changing month lengths in JJAS.

Reviewer #3

(Remarks to the Author)
Review to Millot-Weil et al.:

The paper "Asian monsoon orbital variability directly paced by CO_2 and precession, not eccentricity" investigates how different climate forcings (including insolation, ice sheets, and greenhouse gases) modulate the pace and magnitude of the Asian monsoon, and assesses the relative importance of each forcing using a large ensemble of snapshot simulations spanning the past 800,000 years. The study demonstrates that monsoon variability is predominantly governed by the direct influence of precession and greenhouse gas changes, while eccentricity and ice-sheet variations primarily affect the amplitude of monsoon intensity rather than its pacing. This conclusion contrasts with previous studies, such as Lyu et al. (2022), which emphasized a stronger role for icesheet forcing in driving the 100-kyr cycles. I think this modeling study provides important evidence to an ongoing scientific debate, and I am particularly impressed by the substantial effort invested in conducting comprehensive all-forcing and sensitivity simulations with the HadCM3 model, which is specifically tuned for paleoclimate applications.
However, I find that the discussion section lacks sufficient depth, which is somewhat disappointing. The direct influence of precession on the 20-kyr Asian monsoon pace is clear, consistent, and already well established in previous studies. I would expect the authors to further investigate and discuss the mechanisms through which greenhouse gases, in combination with orbital forcing (though not orbital eccentricity itself), influence the pacing of the Asian monsoon over the 100-kyr and longer cycles, particularly with respect to oceanic precipitation & temperature and land temperature variations. Unfortunately, the discussion is almost entirely devoted to explaining the differences from Lyu et al. (2022). As a result, the manuscript largely reports what the model simulations show, without providing deeper insights into why these responses occur. This leaves the reader without a clearer understanding of the physical and dynamical processes underlying monsoon variability across different climate periods over the past 800,000 years. Moreover, I'm concerned about the reliability and credibility of the

model simulations and associated results (see comments below). I general, from the perspective of novelty and scientific advancement, I'm afraid that the current version may not fully meet the standards of a high-impact journal. Please find the detailed comments and questions below.

Major comments:

(1) The abstract is currently bland and does not clearly convey the central scientific debate, why the findings of this study are important, or how they contribute to advancing our understanding of Asian monsoon variability. Moreover, the novelty of the work is not highlighted. As it stands, the abstract reads more like a list of methods and results rather than a concise statement of the research question, significance, and contribution. I recommend a major revision of the abstract for the broader paleoclimate and monsoon communities.

(2) Line 144-146: "These results suggest on the one hand that eccentricity variations have an indirect impact on temperature and precipitation over the sea through variable greenhouse gas concentrations, and on the other hand, greenhouse gas variations have a negligible impact over the land on the Asian monsoon." and Line 156-157: "Additionally, greenhouse gas variations appear to be responsible for the 100 kyr signal on SSTs but not eccentricity directly." This is interesting results and non-trivial finding, as it points to a complex interplay in how eccentricity signals are transmitted indirectly and differently over ocean versus land. Readers will naturally want to understand the underlying mechanisms behind it. However, the manuscript does not return to this result in the discussion, nor does it attempt to investigate or explain the processes responsible.
The entire discussion is devoted to explaining the differences from Lyu et al. (2022), without returning to non-trivial results and arguments on 100-kyr cycles, this deteriorates the scientific novelty and contribution to the Asian monsoon paleoclimate community. A deeper exploration of why and how eccentricity-driven greenhouse gas variations influence the ocean but exert negligible effects over land would substantially enhance the scientific depth and overall contribution of this work.

(3) Model robustness and results credibility: More efforts are needed to demonstrate the robustness of this new version of the model simulations. It is surprising that different versions of the same HadCM3 model produce such large discrepancies in identifying the dominant forcing of the Asian monsoon over the 100-kyr and longer cycles. This raises concerns about the credibility of the results. I would strongly encourage the authors to validate their findings by performing a similar analysis with an entirely different climate model. In particular, the recently published orbital-accelerated transient simulations of the last 800,000 years conducted with the CESM1.2.2 model (Su et al., 2025) provide an excellent opportunity for direct comparison. Using these simulations to test whether the 100-kyr cycles of Asian monsoon variability are indeed largely driven by CO_2 would greatly strengthen the conclusions of this study.

(4) The authors use five proxy records to validate the model, with only one land-based speleothem record located near ~30°N. I understand that there are very few terrestrial records extending back 800,000 years. However, given the importance of the Asian monsoon domain—particularly its northern margin—I encourage the authors to include additional land-based records, even if they only cover a portion of the entire research period. Incorporating additional land-based records would provide more robust validation of the model simulations of monsoon variability.

Minor comments:
Line 161-162: It is confusing for readers why the manuscript suddenly introduces the global wavelet spectrum and why it is necessary. Providing one or two sentences to clearly state its purpose and how it contributes to the overall argument would greatly improve clarity.

Please define "JJS" in figure caption of Fig. 2. Is "JJS" denote June-July-Augst" or "June-July-September"?

Figures visualization and explanation are not clear enough for arguments.
Line 116-117: "for the strongest monsoons, the Orb set is overall cooler than the two other sets (Fig.(S1)) whereas we observe the opposite pattern for the weakest monsoons time periods." I find it difficult to discern the differences in surface temperature between stronger and weaker monsoon states from shading of Fig. S1 alone. The current presentation does not clearly support the authors' argument. I encourage the authors to add an additional row of subplots in Fig. S2 to directly illustrate the surface temperature differences between stronger and weaker monsoons for the three simulation sets.

Fig. 3 requires improvement. Overlaying the $\delta^{18}O$ records with the corresponding model outputs (SST, temperature, or precipitation) at the same locations would greatly help readers directly assess the similarities and differences between data and simulations. Presenting each time series in a separate subplot not only wastes space but also makes comparison more difficult.

Line 165-166: "In this respect, Fig.(S3) shows precipitation time series
166 for four sub-domains and reveals a similar decoupling…". Please add a map with a framing box to show the location of each sub-domain shown in Fig. S3.

Line 81-82: "temperature" should be "temperature difference or gradient"?

Line 133-134: "Wavelet analyses show significant power at precession timescales (≈21kyr) throughout the 800 kyr apart from around 400 kyr, for every variables studied". and Line 139 "There is also a clear 400 kyr signal present in simulated SSTs and precipitation over the ocean …". However, from Fig. 5, I do not see a "clear" 400-kyr cycle across all sets of simulations. In particular, no spectral peaks above the 95% confidence level are indicated in the 400-kyr band. Could you

clarify how this argument is derived?

References:

Su, B., Sun, Y., & Zhou, M. (2025). Orbital-accelerated transient simulations of glacial-interglacial climate cycles for the last 800,000 years. Scientific Data, 12(1), 961.

Version 1:

Reviewer comments:

Reviewer #1

(Remarks to the Author)

In the revised manuscript, the authors have addressed most of my concerns arose in the first round of review. However, I still have several major concerns that need the authors to address and clarify.

First, the writing of this paper is poor and there are many mistakes in the expression. For instance, many sentences lack the subject and/or objects. I would suggest the authors to ask a polishing company to edit and polish their paper. Nonetheless, I will still point out some obvious issues. For instance, “summer Asian Monsoon” was used in many places of this paper, the correct form should be “Asian summer monsoon”.

Second, the captions/instructions of Tables and Figures are very short and not clear. For instance, “Figure 1: Study area with proxy location from: 1) [39] (Hulu cave) δ18O…”, you should write like this: “Figure 1: The study region and location of proxy records. (1) speleothem δ18O records from Hulu/Sanbao caves in southern China [39]”. In addition, your reference here is wrong. The right one is “Cheng, H. et al. The Asian monsoon over the past 640,000 years and ice age terminations. Nature 534, 640–646 (2016).” This is a composite cave δ18O record over the last 640 kyr, not just from Hulu cave. See the paper above for more details. Similarly, modify the captions of all Tables and Figures to provide sufficient information. I would suggest the authors to read papers published on Nature Communications to see how Tables and Figures are presented.

Third, based on Fig. 5 in the Supplementary file, the precipitation timeseries from southern west part (Indian subcontinent) show strong glacial-interglacial changes (100 kyr cycle) in the All forcing, Orb_Ghg, and Orb_Ice. If this is not a mistake in the labels made by the authors, it is an interesting finding that the Indian summer monsoon rainfall is dominated by glacial-interglacial changes (100 kyr cycle). Why not discuss it in more depth?

Specific comments:

Line 25: “These orbital parameters”, The orbital parameters.
Line 27: “feedback”, have feedback.
Line 28-29: “that can impact the Asian monsoon”, including the Asian monsoon.
Line 50-51: “Moreover, [23] considered not only……”, Moreover, Lyu et al. [23] considered not only……
Line 53: The same to line 50-51.
Line 54: the same.
Line 72: “summer Asian Monsoon”, Asian summer monsoon.
Line 96: “summer Indian monsoon”, Indian summer monsoon.
Line 103: “summer Asian Monsoon”, Asian summer monsoon.
Line 109: “SST”, define it in the first time. Sea surface temperature (SST).
Line 111: “in the basin in question”, in the same basin.
Line 229: “summer Asian Monsoon”, Asian summer monsoon.
Line 229: “South East sector”, East sector.
Line 255: “summer Asian Monsoon”, Asian summer monsoon.
Fig. 5 and Fig. 6: “mousson area from GPCC”, monsoon area from GPCC?
Fig. 6: Some graphs need to adjust their coordinates to make the results clear, for example, the four panels on the right and the two in the lower left.

(Remarks on code availability)

Reviewer #2

(Remarks to the Author)

Summary:

Millot-Weil et al. have revised their manuscript to include some additional analysis of monsoon dynamics in the simulations and discussion in the context of previous work, although their main approach and conclusions remain the same as the prior version of the manuscript. I appreciate that the authors have acknowledged more recent definitions of monsoon circulation and added more in-depth explanation of mechanisms as noted in my previous review. However, I still have some major concerns regarding the analytical framework, communication of the results, and scientific impact of the conclusions relative to previous work in the field, which unfortunately make me question whether this revised manuscript is appropriate for this journal. Please see more detailed comments below.

Minor Comments:
• Many instances of “precipitations”, and other plural words that should be singular “variables” (line 178), “simulations” (line 179)
• Fig S1: No labels on the colorbar
• Many qualitative lines that would benefit from quantitative comparison to make the argument more clear (e.g., Lines 155-158, 164-165)
• Line 161: Switch “simulated” to “resolved”
• Using the term “observations” too much and for different uses, you should use one word more consistently (e.g., line 180 observations is used to described results from Fig. 4 and then also used on line 317 to describe proxy-observations)
• Many sentences where the grammar and sentence structure make it difficult to understand the intended meaning of it (lines 112-114, line 149, lines 202-203)

Major Comments:
• Abstract: this does not read with a strong statement of significance for the paper. We do learn that precession is the most important driver of monsoon pacing, but this has been explained by previous studies (e.g., lines 44-55) and it is not clear why the approach of this study is sufficiently novel to warrant publication in this journal.
• Lines 81-104: I appreciate that you have acknowledged the definition of the monsoon as a variation of atmospheric circulation and location of the ITCZ, rather than a land-sea breeze. However, I understand that you are largely refuting this definition without clear justification. Your new plots of ITCZ location and Hadley circulation (Figs. S3 and S4) show that the ITCZ location indeed shifts poleward during stronger monsoons/stronger summer precipitation, particularly in the East Asian region. This appears to support the ITCZ-based monsoon definition, but you have not included corresponding plots to show the distribution of precipitation and moist static energy which would be needed to evaluate the ITCZ-based definition of a monsoon. Also, on lines 93-95, you claim that “The ITCZ… never attains further than ~15N above the Indian sector, and ~35N above East Asia”. Doesn’t this statement only apply to the weakest summer precipitation cases (top row) of Fig. S3 and S4? I am not sure how these plots refute the ITCZ-based monsoon definition. So together with Fig. 2, I am not convinced of your final interpretation that “in the model and at orbital timescale, the traditional comprehension of the summer Asian monsoon is still accurate”.
• Results & Discussion: The lack of mechanistic explanation for the relative roles of CO2, orbital forcing, and land ice on monsoon variability noted by my previous review and other reviewers is a hinderance to the scientific impact of this study. This is an additional reason why I do not recommend publication in this journal.
• Regarding my comment in the initial review on the paleo-calendar effect, I understand why the authors chose to not incorporate it as it would present a substantial amount of work to calendar adjust all original monthly simulation files. However, I do not believe the statement that “by taking an average over several months, [the authors] smooth the impact of any calendar shift.” Primarily this is because there is a temporally varying spatial imprint of the calendar effect and it is not just a matter of varying month-length anomalies of a few days. I would like to see an acknowledgement of the impact the paleo-calendar effect could have on their results.

(Remarks on code availability)

Reviewer #3

(Remarks to the Author)
The author has reasonably addressed my major comments, and the structure of the paper, as well as the abstract and discussion, have been substantially improved. By including simulations from Su et al. and by comparing the model time series with additional proxy data, the credibility of the model simulations is strengthened and the arguments of the paper are more robust. I therefore recommend that the paper be accepted for publication.

(Remarks on code availability)

Version 2:

Reviewer comments:

Reviewer #1

(Remarks to the Author)
The paper of Jeanne Millot-Weil et al. has been greatly improved based on the comments of reviewers. However, there are still a lot of editorial mistakes and some other minor errors in this version. For the editorial mistakes, it needs the publishing editor to comment and I just list some of them in below.
The major contribution of this paper is that it provides a dataset of climate simulation over the past 800 kyr. The authors also indicate where the data are available. However, I find it very difficult to access the model data based on the link provided by the authors, which hindered the validation of this paper. It is also not conducive to the spread and development of science. Therefore, I strongly suggest the authors to share the model data in ways that are easy to access, such as zenodo or

figshare.
Figure 1:
“1) speleothem” should be “1) Speleothem”.
“2) benthic” should be “2) Benthic”.
“4) δ18O sea water”, 4) Sea water δ18O.
6) foraminiferal, 6) Foraminiferal.
7) foraminiferal, 7) Foraminiferal.
Line 37: “Since then”, revise to “However”
Line 42: “greenhouse gases increasing”, greenhouse gases in increasing.
Line 53: “pCO2”, define it or revise to “CO2 concentration“。”
Line 53-54: “East Asian summer precipitation”, East Asian summer monsoon precipitation.
Line 54: delete “from Chinese loess deposit”.
Line 56-57: “play a primary role on East Asian precipitation intensity”, play a primary role in regulating East Asian monsoon precipitation intensity.
Line 57-60: “Lyu et al. [24] however, considered not only the East Asian monsoon intensity but also its pace over the last 800-kyr and showed a direct impact of ice sheet and precession cycle but this used a Gaussian emulator based on previous HadCM3 simulations.” Recently, Lyu et al. [24] considered not only the East Asian monsoon intensity but also its pace over the last 800-kyr and showed a direct impact of ice sheet and precession cycle. However, this study used a Gaussian emulator, not direct simulations, based on previous HadCM3 simulations.
Line 63: “an emulator based approach”, an emulator-based approach.
Line 79: “pCO2”, CO2 concentration.
Line 82: “Quaternary Asian summer Monsoon”, Quaternary Asian summer monsoon.
Line 84: “out performing”, outperforming.
Line 125-126: “red dots - no2, no3, no4, no5”, what this means?
Line 127: “pink dot - no1”, ???
Line 130: “yellow dots - no7, no6, no8”, ???
Line 131: “yellow dots - no7, no6, no8”, ???
Line 132: “Fig.(3) (a)”, Fig. 3a.
Line 133: “Fig.(3) (b)”, Fig. 3b.
Line 135: “Tab.(2)”, Tab. 2.
Line 146: “Fig.(3) (b)”, Fig. 3b.
Line 146-147: “The average bias between model and proxy SSTs is +1.73 oC and -1.71 oC respectively, in average for the LST”, sick sentence. The average bias between model and proxy SSTs is +1.73 ℃，and the average bias between model and proxy LST is -1.71 ℃?
Line 149: “(Tab.(2))”, Tab. 2.
Line 152-153: “Correlations were not significant according to the p value.”, the correlations between what?
Line 161: “Fig.(S1)”, Fig. S1.
Line 162: “Fig.(S1)”, Fig. S1.
Line 163: “Fig.(S5) & (S6)”, Fig. S5 & S6.
Line 164: “Fig.(S2) & (S3)”, Fig. S2 & S3.
Line 174: “Fig.(S3)”, see above.
Line 179: “Fig. (4)”, “Fig. (1)”
Line 193: “Figs. (S4–S6)”, Figs. S4–S6.
Line 201: “Fig. (1)”, Fig. 1.
Line 202: “Fig.(5)”
Line 206: “Fig.(4)”
Line 207: “Fig.(1)”
Line 209: “Fig.(5)”
Line 218: “Fig.(5)”
Line 224: “Fig.(2)”
Line 229: “Fig.(6)”
Line 230: “Fig.(5)”, “Fig.(6)”
Line 232: “Fig.(1)”
Line 235-236: “Fig.(S4 & S5”
Line 236: Fig.(S7)
Line 238: “South East summer Asian monsoon domain”, southeastern Asian summer monsoon domain.
Line 251-252: “Fig.(S1), (S2) and (S3)”
Line 264: “east Asian summer monsoon”, East Asian summer monsoon.
Line 272: “Fig.(7)”, “Fig.(5)”
Line 273: “Fig.(7)”
Line 280: “GHG’s”, greenhouse gases
Line 281: “at 100 kyr. Thus confirming”, at 100 kyr, thus confirming
Line 283: “GHG”, greenhouse gases, or define it in the first time you use it.
Line 289: “Northern hemisphere”, Northern Hemisphere
Line 304: “Fig.(8)”
Line 306: “Fig.(8)”
Line 307: “A persistent 100-kyr signal emerges in the Northern part of the East Asian region (15-20◦N)”, revise “Northern part” to “southern part”.
Line 308: “Fig.(8)”

Line 315: “around 400kyr”, at around 400kyr
Line 322: “Fig.(6) and Fig.(S7)”
Line 330: “Fig.(2)”
Line 331: “Tab. (2)”
Line 356-358: “These results also motivate further process-based analysis to better quantify the contribution of orbital-scale forcings shape atmosphere dynamics that underpin monsoon behaviour”, revise it for better understanding.

(Remarks on code availability)

Reviewer #2

(Remarks to the Author)
I appreciate the revisions that Millot-Weil et al. have made to their manuscript. The current version of the manuscript has stronger scientific justification and writing. They have broadened the definition of a monsoon and investigated it more thoroughly, corrected grammatical errors, and evaluated the potential impact of the paleo-calendar effect on their results. Improvements to the text also provide a clearer explanation of the advancement in methodology that has been made compared to previous studies on similar topics (e.g., Lyu et al. [24], Lu et al.[25], Su et al., [46]). While I believe this paper will be a great fit in another journal, I do not believe the results are not impactful enough for publication in Nature Communications. Here are a few comments that detail my rationale:

Major Comments
- One of the main conclusions of the paper is a “precipitation over the entire region is strongly correlated with the land-ocean temperature gradient” (line 256-257), this does not provide an improved mechanistic understanding of monsoon drivers, which is possible to more thoroughly investigate with full-complexity climate model simulations. I believe that the additional analyses you have done in the supplement (Figure S2, S4, S5, S6) help demonstrate that the spatial extent and drivers of the monsoon is more complicated than land-sea thermal contrast.
- One additional main finding of the study is that “Spatial heterogeneity in monsoon dynamics reconciles conflicting proxy records, linking discrepancies to regional characteristics and different sensitivities to imposed forcings” (lines 9-11). This implies that you have improved our understanding of mechanisms driving complex signals archived proxy records in the region. Instead, my understanding is that you have shown that the “Pearson’s correlation (Tab.(2)) between the geological data and model show significant correlation between proxy and model temperatures, although higher coefficients are found for the SST rather than for LST” (lines 148-150). So you do not provide a mechanistic explanation of regionally conflicting proxy records, which would drive higher impact of the study.
- You explain that “our analysis encompasses a wide domain, whereas the Asian summer monsoon comprises two distinct subsystems (South Asian sector and East Asian sector) which differ in their dynamics and are often studied separately” (lines 257-259) and ultimately I believe this limits the impact of the study because the results more general, rather than providing a detailed mechanistic understanding that could come from a more constrained, regional analysis.

Minor comment
Line 227: forcing is misspelled

(Remarks on code availability)

Version 3:

Reviewer comments:

Reviewer #1

(Remarks to the Author)
Thank the authors for carefully revise their paper again. I have reviewed this paper three times previously, and I do not want to comment on the grammar anymore. For my major concern, the data are still difficult to access. Although the authors replied that “the size and quantity of simulations amount for several TBs”, the size of one month of simulation is only 33 KB. For monthly precipitation data, there are 100 years for one snapshot, 12 months per year, and 219 snapshots in total, the size is only about 8 GB. When adding temperature data, the size is only ~16 GB. This is not too large to be stored on public websites such as zenodo or figshare. In the website provided by the authors, one need to repeat 12*219 = 2628 times to get the data of one climate variable, this is really a big and unnecessary work. In summary, the authors should make their data

easy to access, even only the long-term monthly mean of each snapshot is provided. This is where the value of this paper lies.

(Remarks on code availability)

Reviewer #2

(Remarks to the Author)
Thank you to Millot-Weil et al. for providing a detailed analysis of the land-sea thermal contrast and its relationship with seasonal heat fluxes across the different simulations. The authors have reasonably addressed my comments. I do not find the simulation data to be accessible through the BRIDGE web pages, as the webpages appear to be only summary statistics/plots and the raw simulation output is archived to tape. Perhaps this is reasonable given the size of the dataset, but I am unable to test the scripts associated with the analysis.

(Remarks on code availability)
I am unable to run the code because the simulation output is not accessible.

Reviewer #4

(Remarks to the Author)
The orbital forcing of Asian monsoon evolution is a key research topic in Quaternary paleoclimatology and Milankovitch theory. Using the full-complexity HadCM3B model, the authors performed high-resolution snapshot simulations of paleoclimatic changes over the past 800 ka, quantified the impacts of distinct forcings, and re-evaluated the respective roles of orbital eccentricity and atmospheric CO2 in modulating the Asian summer monsoon (ASM). This study presents solid paleoclimate modeling work and puts forward meaningful hypotheses regarding the dynamical mechanisms governing ASM variations. Overall, the authors have adequately addressed the previous reviewer comments and implemented corresponding revisions throughout the manuscript. Below are two major suggestions and one minor discussion point for the authors' reference.
First, both the title and main conclusions indicate that precession and greenhouse gas variability jointly dominate the long-term evolutionary pacing of the ASM. However, the abstract only highlights the dominant control of greenhouse gases on the 100-kyr ASM periodicity while neglecting the contribution of precession. This inconsistency renders the abstract incomplete and inconsistent with the main findings of the study, and corresponding revisions are therefore recommended.
Second, the statement in Lines 247-248—"and the significant 100 kyr power in both land and ocean precipitation is reduced or removed when ice sheet forcing is included"—is insufficiently objective and requires revision. Notably, the 100-kyr spectral power of land precipitation exhibits no statistical significance even in simulations excluding ice sheet forcing, which contradicts the above description.
In Lines 304-318, the authors attribute the discrepancies between their results and previous studies to differences in simulation strategies, and acknowledge that snapshot simulations may induce biases in the representation of deep-ocean circulation. It is well established that changes in Northern Hemisphere high-latitude insolation and ice sheet exert profound impacts on the Atlantic Meridional Overturning Circulation (AMOC). Substantial evidence from both orbital-scale paleoclimatic reconstructions and modern simulations suggests a tight linkage between AMOC variability and Asian monsoon changes (Cheng et al., 2016; Dong et al., 2025). Given that the model may inadequately resolve deep-ocean circulation, it is worth discussing whether such model limitations account for the absence of long-period cyclicity in the simulated ice-volume and orbital-forcing responses. This is merely an open scientific question for academic discussion and does not require mandatory revisions to the current manuscript.
Overall, the manuscript is well-organized and provides valuable insights into Asian monsoon dynamics. I recommend publication pending minor revisions as suggested above.

Ref:
Cheng, H.*, Edwards, R.L.*, Sinha, A., Spötl, C., Yi, L., Chen, S.T., Kelly, M., Kathayat, G., Wang, X.F., Li, X.L., Kong, X.G., Wang, Y.J., Ning, Y.F., Zhang, H.W., The Asian monsoon over the past 640,000 years and ice age terminations, Nature, 534(7609)(2016), 640-646.
Dong, X.Y.*, Chen, R.S., Niu, X.W., Hummel, C., Kathayat, G., Liu, Y.T., Zhao, J.Y., Zhang, H.W., Shi, Z.G., Zhang, X., Yang, Y., Cheng, H.*, Early warning signals for Asian summer monsoon tipping and implications for future monsoon changes, The Innovation Geoscience, 3(4)(2025), 100158-1-100158-9.

(Remarks on code availability)

Open Access This Peer Review File is licensed under a Creative Commons Attribution 4.0 International License, which permits use, sharing, adaptation, distribution and reproduction in any medium or format, as long as you give appropriate credit to the original author(s) and the source, provide a link to the Creative Commons license, and indicate if changes were made.
In cases where reviewers are anonymous, credit should be given to 'Anonymous Referee' and the source.
The images or other third party material in this Peer Review File are included in the article's Creative Commons license, unless indicated otherwise in a credit line to the material. If material is not included in the article's Creative Commons license and your intended use is not permitted by statutory regulation or exceeds the permitted use, you will need to obtain permission directly from the copyright holder.
To view a copy of this license, visit https://creativecommons.org/licenses/by/4.0/

Response to Reviewers Comments

Reviewer #1 (Remarks to the Author):

The evolution of Asian summer monsoon since the mid-Pleistocene transition (~700 ka) has long been debated over the past few decades, with loess, lake, and marine records showing a strong eccentricity cycle (100 kyr) while the speleothem δ18O records showing a strong precession cycle (200 kyr), which is known as “Chinese 100 kyr problem” (Cheng et al., 2021). Also see Li et al. (2024) for an introduction.

The paper by Millot-Weil et al. investigates the Asian summer monsoon over the past 800 ka using climate simulation at 219 snapshots performed with the HadCM3 model, and they concluded that Asian summer monsoon variations are predominantly driven by the direct influence of precession and greenhouse gas changes, and the role of eccentricity and ice sheet variations are neglectable, which only impact the monsoon amplitude (monsoon intensity), but not its pace.

I am not an expert on climate model simulation. But I find several mistakes in this paper about the basic concept of monsoon definition, which may affect the conclusion of this paper. Also, there are serious problems in the writing of this paper, which are not in accordance with academic ethic. For instance, several proxy records are mentioned and presented in this paper, but the original references are not properly cited. Moreover, there are many technical errors in this paper.

Since the analysis methods their conclusion relies on are questionable, I cannot judge whether the conclusion is reliable.

Thank you very much for your insights and helpful suggestions, we have made changes to the manuscript accordingly which have improved the paper.

Details are as follow:

1. The Asian monsoon includes both summer monsoon and winter monsoon. This paper only investigates the summer monsoon. The author should make it clear in the title and other area in the manuscript.

The manuscript has been modified to make this clearer that we only focus on the summer monsoon. The title has been modified as well to avoid any misunderstanding.

2. Definition of monsoon domain. The author used the region (lat: 0-50 N, lon: 40-130 E, in their Fig. 2-3) to represent the Asian summer monsoon domain. This is incorrect, because this region also includes the westerly region (arid region) and the equatorial region, which are not Asian summer monsoon region.

While absolutely true, such a region is often used for modelling studies to characterise the monsoon region. The difficulty here is that using a pure Modern monsoon domain could mean that an increase in the domain size could be missed (i.e. under some time periods perhaps the arid region is no longer arid with the monsoon extending into it).

However, we agree that using domain defined by observations is warranted and have carried out the same set of analyse with it. After comparing both methods we show that the conclusions are unaffected by either choice, however in some instances there is an increase in some correlations. We have chosen to include the observed Modern domain in the manuscript now as suggested.

The summer monsoon climate is characterized by rainy summer and dry winter, and the accompanied reversal of wind direction between the summer and winter (Wang & Ding, 2008). The definition of summer monsoon domain has been clearly shown in previous studies. For instance, the global monsoon domain is defined as the region where local summer-minus-winter precipitation rate exceeding 2.0 mm/day and the local summer precipitation exceeding 55% of the annual total (Wang et al., 2012). The local summer is from May to September (MJJAS) for Northern Hemisphere and from November to March (NDJFM) for Southern Hemisphere.

In the Method section, the author should follow these studies to clearly define the monsoon region where they use to calculate the Asian summer monsoon rainfall change. After this change, the author should re-perform all calculations. This change may affect the conclusion of this paper and the author should revise their manuscript accordingly. Since Asian summer monsoon rainfall is mainly occur on land, I suggest the author use the Global Precipitation Climatology Centre (GPCC) rainfall data to define the Asian summer monsoon region. The GPCC climatology data can be found here:
(https://opendata.dwd.de/climate_environment/GPCC/html/gpcc_normals_v2022_doi_download.html)

The domain of study has been narrowed to exclude the western arid part of the region. The new domain of study is delimited between 0 and 50 degrees North in latitude and between 60 and 130 degrees East in longitude. We employed the Global Precipitation Climatology Center rainfall data between 1951 and 2000 at 2.5 degree of grid resolution (the closest to the model one). We have chosen the oldest time slice available on the database to avoid as much as possible anthropogenic greenhouse gas impact. From these rainfall data, we calculated where the local summer(MJJAS)-minus-winter(NDJFM) precipitation rate exceeds 2.0 mm/day and the local summer precipitation exceeding 55% of the annual total (as was previously done). The area obtained is shown in Fig.(1), on the blue hatched area. The summer monsoon precipitation and precipitation over land used in the analysis has been computed using this summer monsoon precipitation area.

3. The summer season is not clearly defined. It seems that the author used June-to-September for calculation, but they showed JJS in Fig. 2, what this means? Just misspell? I suggest them to analyze both JJA (months 6-7), MJJAS (months 5-9), and annual rainfall, how the different?

We apologise for the confusion, JJS is shorthand for June-September. The analysis have been re-conducted for annual rainfall, JJA and MJJAS (see the following figures in this order). JJA and MJJAS both show a strong correlation between the land-sea thermal contrast and precipitation, which disappears when looking at just the annual mean. This shows strong support for a monsoon signal. For here on in, we have chosen to perform our analysis using MJJAS to better match the description of the summer monsoon area.

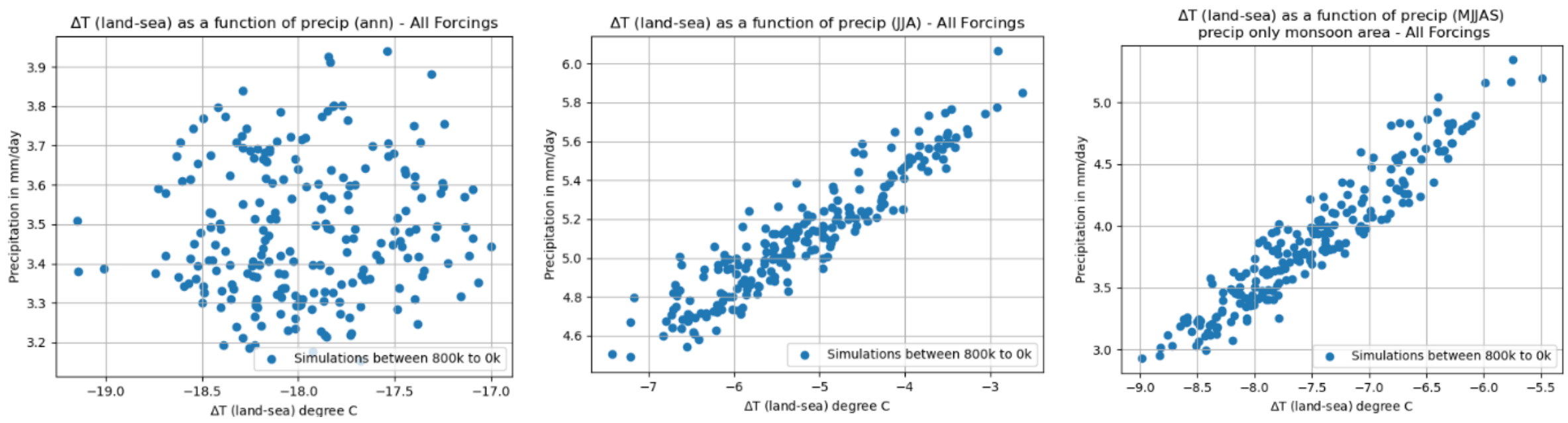

Fig: Relationship in All_forcings simulations set between precipitation and temperature contrast between the land and the sea in annual mean (left plot), in June-July-August (middle plot), and in May-June-July-August-September (right plot).

4. Many proxy records were not properly cited. Especially, the original references were not cited. For instance, the Hulu/Sanbao speleothem δ18O records were presented in Fig. 2, but without citation and the original reference were not listed. Note that the review paper by Sun et al. (2022) cannot replace the original reference.

Good spot, thank you. The references of the geological records used to perform the model-data comparison have been modified to include the original references.

5. In the Introduction section, the review of literature is inappropriate and can not truly reflect the opinion of the original references. First, the author may not read the original references carefully and proxy records from different time periods were discussed and compared together, which is not objective, leading to artificial bias. For instance, the reference of Sun et al. (2006) indicated that "Spectral analyses of the stacked monsoon proxies indicate that characteristics of both the summer and winter monsoons are dominated mainly by variance in the eccentricity (410- and 100-kyr), obliquity (41-kyr) and precession (23- and 19-kyr) bands over the past 3.4 Myr". However, Millot-Weil et al. stated that "loess deposits and stable oxygen isotopes from Chinese speleothem are dominated by obliquity (≈40 kyr) and precession pacing (≈20 kyr)".

The geological studies were more carefully read and the introduction has been reworded to avoid misinterpretation or biased statement that cannot truly reflect the literature.

6. The format of references is wrong and unprofessional.

The format of references has been modified using the "nature" style from the LateX package biblatex. If the references format still needs to be improved to meet the journal's formatting requirements, we will modify it according to the editor's demand.

7. The method used to assess model's ability to simulate accurately the Asian monsoon system is not correct. The model's ability to simulate modern monsoon climate should be comparing the model results at modern time with modern observations, including rainfall distribution, wind field, and so on.

We agree, such an analysis is vitally important. Indeed, the model's ability to simulate accurately the present-day Asian monsoon has already been assessed through a CMIP5 models intercomparison and can be found in (Sperber et al., 2013) as well as many other studies. Indeed, compared to other CMIP5/6 models it is still one of the best at reproducing

the mean state, hit rate and monsoon domain for East Asia. This reference has been mentioned in the Assessment part of the study. The model has also been successfully employed for a range of different time periods to investigate the impact of the monsoon showing good skill at reproducing paleo-observations (e.g. Farnsworth et al. 2019).

8. Line 88: δ18O records from marine cores and speleothem are not a temperature proxy. Planktonic foraminifera δ18O records are a proxy for salinity, and Benthic foraminifera δ18O records are a proxy for deep sea temperature and global ice volume. Sea surface temperature (SST) has been reconstructed by Mg/Ca ratio in many studies, and they are the one should be compared with model results of SST. There are also many Land temperature records (Lu et al., 2019, 2022), which can be compared with land surface temperature (LST) from model results.

Thank you for this suggestion. The model-data comparison part has been changed to clarify the choice of the records used. δ18O records are used to assess the variability of the model outputs rather than comparing with temperature value. To compare the temperature values from the model with geological reconstructions we have included; for sea surface temperature reconstruction Mg/Ca ratio recorded in the Arabian sea (Tarique et al., 2023), in the Bay of Bengal (Govil et al., 2011) and in the South China sea (Wei et al., 2007), and for the land surface temperature, three reconstructions from loess records (Lu et al., 2019) (Lu et al., 2022). Figure 3 has been changed to include these new records and to make the comparison easier to see.

9. Line 115-117: “for the strongest monsoons, the Orb set is overall cooler than the two other sets”, cooler? Are you sure? The same for the weak monsoons.

Thank you for raising the mistakes. The calculation has been run again and visible on the legend of the Fig S1. However, instead of classifying the weakest and strongest by looking at the mean summer monsoon precipitation (bigger or smaller than the global mean across the entire 800-kyr), we have chosen to define the weakest as the one with weaker summer rainfall than the 1rst quantile and the strongest as the one with stronger rainfall that the 3rd quantile (quantiles calculated using the entire time slice).

10. What JJS on Fig. S1 means? JJAS?

See above for JJS explanation. This has now been modified to MJJAS in line with the earlier recommendation from the reviewer.

11. “geographic repartition” in the paper is inappropriate, “spatial distribution” should be more appropriate?
This has been modified.

12. Line 191: East Asian region (15-20◦N) is not Northern portion, but Southern portion.

This has been modified.

13. The edge effects might distort the result of wavelet power spectrum analysis, and the cone of influence (COI) should be performed and shown (Grinsted et al., 2004).

The cone of influence is now included to the wavelet power spectra, following the method presented in (Grinsted et al., 2004).

14. The model data used for analysis is essential for repeatability and verifiability of this study, as well as for comparison and dispersing (citation) in the research community, the model data should be stored on public data website like Zenodo or Figshare. A Data availability statement is also needed to comply with the journal policy.

Statement for data and code availability have been added at the end of the manuscript, stating the location of the publicly available model datasets and scripts used here.

The literature I mention in the comments:

Cheng, H. et al. Orbital-scale Asian summer monsoon variations: Paradox and exploration. Science China Earth Sciences 64, 529-544 (2021).
Li, X. et al. Loess deposits in the low latitudes of East Asia reveal the ~20-kyr precipitation cycle. Nature Communications 15, 1023 (2024).
Wang, B. & Ding, Q. Global monsoon: Dominant mode of annual variation in the tropics. Dynamics of Atmospheres and Oceans 44, 165-183 (2008).
Wang, B., Liu, J., Kim, H.-J., Webster, P. J. & Yim, S.-Y. Recent change of the global monsoon precipitation (1979–2008). Climate Dynamics 39, 1123-1135 (2012).
Sun, Y. et al. A review of orbital-scale monsoon variability and dynamics in East Asia during the Quaternary. Quaternary Science Reviews 288, 107593 (2022).
Sun, Y., Clemens, S. C., An, Z. & Yu, Z. Astronomical timescale and palaeoclimatic implication of stacked 3.6-Myr monsoon records from the Chinese Loess Plateau. Quaternary Science Reviews 25, 33-48 (2006).
Grinsted, A., Moore, J. C. & Jevrejeva, S. Application of the cross wavelet transform and wavelet coherence to geophysical time series. Nonlinear processes in geophysics 11, 561-566 (2004).
Lu, H. et al. 800-kyr land temperature variations modulated by vegetation changes on Chinese Loess Plateau. Nature Communications 10, 1958 (2019).
Lu, H. et al. Decoupled Land and Ocean Temperature Trends in the Early-Middle Pleistocene. Geophysical Research Letters 49, e2022GL099520 (2022).

References used in the response:

Sperber, K.R., Annamalai, H., Kang, IS. *et al.* The Asian summer monsoon: an intercomparison of CMIP5 vs. CMIP3 simulations of the late 20th century. *Clim Dyn* **41**, 2711–2744 (2013). https://doi.org/10.1007/s00382-012-1607-6

Mohd Tarique et al. “Enhanced CO2 Degassing From the Tropical Indian Ocean During Cold Climatic Events of the Last Glacial Cycle”. In: Paleoceanography and Paleoclimatology 38.12 (Dec. 2023), e2022PA004570. issn: 2572-4517, 2572-4525. doi: 10.1029/2022PA004570. url: https://agupubs.onlinelibrary.wiley.com/doi/10.1029/2022PA004570 (visited on 10/22/2025).

Pawan Govil and Pothuri Divakar Naidu. “Variations of Indian monsoon precipitation during the last 32kyr reflected in the surface hydrography of the Western Bay of Bengal”. In: Quaternary Science Reviews 30.27 (Dec. 2011), pp. 3871–3879. issn: 02773791. doi: 10 . 1016 / j . quascirev . 2011 . 10 . 004. url: https ://linkinghub.elsevier.com/retrieve/pii/S0277379111003180 (visited on 10/22/2025).

Gangjian Wei et al. “High-resolution sea surface temperature records derived from foraminiferal Mg/Ca ratios during the last 260 ka in the northern South China Sea”. In: Palaeogeography, Palaeoclimatology, Palaeoecology 250.1 (June 2007), pp. 126–138. issn:

00310182. doi: 10.1016/j.palaeo.2007.03.005.url:https://linkinghub.elsevier.com/retrieve/pii/S0031018207001526 (visited on 10/22/2025).

Hongxuan Lu et al. “800-kyr land temperature variations modulated by vegetation changes on Chinese Loess Plateau”. In: Nature Communications 10.1 (Apr. 29, 2019), p. 1958. issn: 2041-1723. doi: 10.1038/s41467-019-09978-1. url: https://www.nature.com/articles/s41467-019-09978-1 (visited on 10/03/2025).

Hongxuan Lu et al. “Decoupled Land and Ocean Temperature Trends in the Early-Middle Pleistocene”.In: Geophysical Research Letters 49.17 (Sept. 16, 2022), e2022GL099520. issn: 0094-8276, 1944-8007. doi:10.1029/2022GL099520. url:https://agupubs.onlinelibrary.wiley.com/doi/10.1029/2022GL099520(visited on 10/01/2025)

Reviewer #2 (Remarks to the Author):

Summary
Millot-Weil et al. used three sets of HadCM3 simulations to disentangle the impacts of orbital forcing, greenhouse gases, and ice sheets on Asian monsoon pacing over the last 800,000 years. They are particularly interested in better understanding the controlling processes that lead to large variations in monsoon pacing on longer timescales, which has been long debated in the literature with differing perspectives from modeling and proxy-based studies. The authors found that precession primarily modulates monsoon variability (temperature and precipitation), confirming findings of previous studies. Greenhouse gases impose a ~100 kyr signal on SST variability associated with the monsoon. Including ice sheet variations dampens the impacts of precession and GHG variations, but does not alter the monsoon’s pace. The simulation set is uniquely designed to provide a valuable contribution to our understanding of Asian monsoon variability in the past and reveals some new information about Asian monsoon pacing over long timescales. The reported results are reasonably supported by the analysis and simulations. However, I do have a few major concerns with the current scope of the paper that I will describe in more detail below.

Thank you very much for the all the thoughtful suggestions, they have certainly helped improve our manuscript.

Minor comments

• Line 23 should be speleothems (?)
• Line 43 should be ice sheets
• Line 73 perhaps switch “orbits” to “orbital cycles”
• Line 202 should be “proxy-model comparison”
• Line 222 “an other” should be “another”

• Figure 2, Figure S1, Figure S2: Why is N Hemisphere summer defined as JJS or June-July-September, omitting August? Is this is a typo (should be JJAS)?

Thank you for catching these misspelled words and typo. They have been modified. For Fig(2), Fig(S1) and Fig(S2) the legend has been changed to MJJAS (May, June, July, August and September; as suggested by Reviewer 1) because we modified our months of study to these months (rather than JJAS).

Major comments

• Lines 31 to 34 (also stated on Lines 77-79): the traditional view of monsoonal circulation as a large-scale sea breeze driven by land-sea contrast has been challenged by theories that characterize monsoonal circulation with variations in the Intertropical Convergence Zone and moist static energy (e.g., Gagdil 2018, https://doi.org/10.1007/s12040-017-0916-x; Geen et al., 2020, https://doi.org/10.1029/2020RG000700). Do your simulations suggest that the traditional perspective is correct? It would be helpful to acknowledge these other theories in your introduction and then again acknowledge these theories in Section 2.1 in the context of describing Figure 2.

A good suggestion. The former version of the paper was only taking into account the traditional description of the monsoon circulation while more recent works provide another understanding of the monsoon system and driving dynamics which characterize it via a seasonal shift of the Intertropical Convergence Zone (ITCZ) and moist static energy. This more recent view has been acknowledge in both the introduction (lines 33-36) as well as helping contextualise some of our Results part (lines 77–80 and lines 86-100). Further analysis have also been added to the supplementary to see Hadley circulation and vertical winds over two Asian domains (Indian sector and East Asian sector) for the four simulations sets (weaker and stronger monsoons) (Fig.(S3 & S4)) which allows use to delineate the location of the ITCZ and sea-breeze imprint of the monsoon. These figures reveal a change in intensity/extent of the sea-breeze circulation rather than a latitudinal ITCZ shift is responsible. This suggests that, at orbital-time scale, the traditional view on the monsoon system is valid. On deeper (>5 Ma) timescales during warmer climates of the past, we have seen a significant shift in the ITCZ (expansion as well as contraction in some periods). However, for Quaternary time periods large deviations in the locations of the summer position of the ITCZ were not shown in these sets of simulations.

• Section 2.2: I appreciate that you disentangle the monsoon pacing associated with orbital, greenhouse gas, and ice variations. These are interesting results. However, I am curious about the climate dynamics associated with the introduction of greenhouse gas and ice sheet forcing, which can be addressed by the simulations. For example, why does the introduction of varying greenhouse gases make eccentricity scale-variations in SSTs apparent? Also, is the mechanism that causes ice sheets to influence monsoon intensity in your simulations similar to previous studies (e.g., citations on Lines 175-177) and why does this mechanism not impact pacing?

A great point raised! Understanding the climate dynamics associated with the introduction of different orbital-scale parameters would be very interesting but this question is beyond the scope of the study. Indeed, while Fig(S1), Fig(S2), Fig(S3&S4) can give some hints about how the intensity of different variables (temperature, precipitation, streamfunction) is affected by different combinations of orbital-scale parameters, we cannot see any drastic differences on the climate circulation that can help us to better understand the mechanisms, mainly

because we are showing averages of several simulations with different boundary conditions. When looking at large spectrum of variability, the only way to gain understanding of the mechanisms is to switch off one of the orbital-scale parameters and see how the timeseries are affected by it, both in amplitude (intensity) and pace. To investigate the dynamics, we would need to shorten the timescale, and rather focus on individual dates to run sensitivity studies to plainly focus on the climate circulation. The limitations of the presented study have been clearly mentioned in the discussion (lines 223-228).

Concerning the eccentricity signal above the sea when adding the CO2 forcing, we can only raise different hypothesis: comparison with (Lyu et al.2019) and Fig(S5) are showing a 100-kyr cycle only in the most southern parts of the domain, thus signal above the sea could also reveal a stronger implication of the greenhouse gases near the Equator, or we could think that more complex interactions over the land are interacting (topography for example) with the temperature and precipitation signals.

These questions are all very interesting and future works will be undertaken to further investigate the role of the orbital-scale parameters on a time scale that corresponds to the monsoon dynamics.

• When you aggregate model output by N Hemisphere summer (likely JJAS, see minor comment above), how do you account for the impact of orbital forcing on the varying length of fixed-angular months over time (i.e., the paleo-calendar effect, e.g., Bartlein & Shafer, 2019, https://doi.org/10.5194/gmd-12-3889-2019)? If you are using fixed-length monthly output from the simulation based on a modern calendar, your results include the effects of orbital variations on insolation and the changing month lengths in JJAS.

Thank you for raising this question. After careful consideration, we have decided to not modify our code to account for the impact of the paleo-calendar effect as we thought it would have a negligible impact on our results because we use the average of our variables over 5 months (MJJAS) (~150 days). Indeed, (Bartlein & Shafer, 2019) present month-length anomalies of -3 or + 3 days (first figure of the paper). By taking an average over several months, we smooth the impact of any calendar shift.

Reviewer #3 (Remarks to the Author):

Review to Millot-Weil et al.:

The paper “Asian monsoon orbital variability directly paced by CO□ and precession, not eccentricity” investigates how different climate forcings (including insolation, ice sheets, and greenhouse gases) modulate the pace and magnitude of the Asian monsoon, and assesses the relative importance of each forcing using a large ensemble of snapshot simulations spanning the past 800,000 years. The study demonstrates that monsoon variability is predominantly governed by the direct influence of precession and greenhouse gas changes, while eccentricity and ice-sheet variations primarily affect the amplitude of monsoon intensity rather than its pacing. This conclusion contrasts with previous studies, such as Lyu et al.

(2022), which emphasized a stronger role for icesheet forcing in driving the 100-kyr cycles. I think this modeling study provides important evidence to an ongoing scientific debate, and I am particularly impressed by the substantial effort invested in conducting comprehensive all-forcing and sensitivity simulations with the HadCM3 model, which is specifically tuned for paleoclimate applications.
However, I find that the discussion section lacks sufficient depth, which is somewhat disappointing. The direct influence of precession on the 20-kyr Asian monsoon pace is clear, consistent, and already well established in previous studies. I would expect the authors to further investigate and discuss the mechanisms through which greenhouse gases, in combination with orbital forcing (though not orbital eccentricity itself), influence the pacing of the Asian monsoon over the 100-kyr and longer cycles, particularly with respect to oceanic precipitation & temperature and land temperature variations. Unfortunately, the discussion is almost entirely devoted to explaining the differences from Lyu et al. (2022). As a result, the manuscript largely reports what the model simulations show, without providing deeper insights into why these responses occur. This leaves the reader without a clearer understanding of the physical and dynamical processes underlying monsoon variability across different climate periods over the past 800,000 years. Moreover, I'm concerned about the reliability and credibility of the model simulations and associated results (see comments below). I general, from the perspective of novelty and scientific advancement, I'm afraid that the current version may not fully meet the standards of a high-impact journal. Please find the detailed comments and questions below.

Thank you very much for your thorough assessment and time taken to review our manuscript, your comments have been particularly helpful in reframing some of our arguments and further implications.

Major comments:

(1) The abstract is currently bland and does not clearly convey the central scientific debate, why the findings of this study are important, or how they contribute to advancing our understanding of Asian monsoon variability. Moreover, the novelty of the work is not highlighted. As it stands, the abstract reads more like a list of methods and results rather than a concise statement of the research question, significance, and contribution. I recommend a major revision of the abstract for the broader paleoclimate and monsoon communities.

A fair point, the original abstract was a bit too forensic. The abstract has now been reworked to better convey the scientific debate by mentioning the tangled impact of orbital-scale parameters and the novelty of the work, notably the method used which allows to encompass long time scale with climate model simulations, and reveals direct influence of precession and greenhouse gas changes on Asian summer monsoon's pace while ice sheet variations impact the amplitude of the changes, at a large space scale.

It has also been shorten to meet the requirement of the editor (150 words or less).

(2) Line 144-146: "These results suggest on the one hand that eccentricity variations have an indirect impact on temperature and precipitation over the sea through variable greenhouse gas concentrations, and on the other hand, greenhouse gas variations have a negligible impact over the land on the Asian monsoon." and Line 156-157: "Additionally, greenhouse gas variations appear to be responsible for the 100 kyr signal on SSTs but not eccentricity directly." This is interesting results and non-trivial finding, as it points to a

complex interplay in how eccentricity signals are transmitted indirectly and differently over ocean versus land. Readers will naturally want to understand the underlying mechanisms behind it. However, the manuscript does not return to this result in the discussion, nor does it attempt to investigate or explain the processes responsible.
The entire discussion is devoted to explaining the differences from Lyu et al. (2022), without returning to non-trivial results and arguments on 100-kyr cycles, this deteriorates the scientific novelty and contribution to the Asian monsoon paleoclimate community. A deeper exploration of why and how eccentricity-driven greenhouse gas variations influence the ocean but exert negligible effects over land would substantially enhance the scientific depth and overall contribution of this work.

A very fair point, on reflection we agree that too much time was devoted to comparing with Lyu, et al. (2021). Partly this is because we are keen to show that accelerated and emulating methods have a distinct drawback. However, understanding the underlying mechanisms responsible for the various regional response of the orbital-scale parameters is indeed very interesting and this question needs to be addressed. However, a full diagnostic disentanglement of the drivers/mechanisms for each series is beyond the scope of the study. When looking at large spectrum of variability, the only way to gain understanding of the mechanisms is to shut down one of the orbital-scale parameters and see how the timeseries are affected by it, both in amplitude (intensity) and pace. To investigate the dynamics, we would need to shorten the timescale, and rather focus on individual dates to run sensitivity studies to plainly focus on the climate circulation. Indeed, Fig(S1), Fig(S2), Fig(S3&S4) can give some hints about how the intensity of different variables (temperature, precipitation, streamfunction) is affected by different combinations of orbital-scale parameters, we cannot see any drastic differences on the climate circulation that can help us to better understand the mechanisms, mainly because we are showing averages of several simulations with different boundary conditions.

Future works will be undertaken to further investigate the role of the orbital-scale parameters on a time scale that corresponds to the monsoon dynamics.

These limitations have been clearly added to the paper (lines 223-228).

(3) Model robustness and results credibility: More efforts are needed to demonstrate the robustness of this new version of the model simulations. It is surprising that different versions of the same HadCM3 model produce such large discrepancies in identifying the dominant forcing of the Asian monsoon over the 100-kyr and longer cycles. This raises concerns about the credibility of the results. I would strongly encourage the authors to validate their findings by performing a similar analysis with an entirely different climate model. In particular, the recently published orbital-accelerated transient simulations of the last 800,000 years conducted with the CESM1.2.2 model (Su et al., 2025) provide an excellent opportunity for direct comparison. Using these simulations to test whether the 100-kyr cycles of Asian monsoon variability are indeed largely driven by CO_2 would greatly strengthen the conclusions of this study.

The discrepancy of the results obtained between (Lyu et al., 2019) and ours does raise a question of credibility, especially since they used the same climate model, although a different version of it and they used different methods (e.g. statistical emulation) than ours to

produce their timeseries. To go further in the comparison between their outcomes and ours, we provide an additional set of simulations called Orb_Ice (including orbital parameters and ice sheet changes but greenhouse gases are set constant at pre-industrial level) that compare directly with their "OrbIce". The wavelet power spectra of this Orb_Ice timeseries is shown on Fig.(8) and show comparable results than in (Lyu et al., 2019): significant precession period for every latitude slices and significant eccentricity period for the two most Southern slices (15-20degree and 20-25degree). As such, our results only differ by the contribution of the greenhouse gases to explain the 100-kyr cycle on this localised area.

Additionally, as suggested by the reviewer, we took advantage of the recent work from (Su et al., 2025) giving transient accelerated simulations covering the last 800-kyr to further test the robustness of our results. Fig.(7) shows similar wavelet power spectra using (Su et al., 2025) simulations (ALL, CO2, ICE, ORB) than presented in Fig.(5) and Fig.(S6) shows the comparable wavelet power spectra to (Lyu et al., 2019) works and our Fig.(8). It is important to note that CO2 and ICE cannot be fully comparable with our respective Orb_Ghg and Orb_Ice, since they do not include orbital parameters changes. However, (Su et al., 2025) show equally for CO2 and ICE a significant eccentricity signal in Fig.(7) suggesting that both forcings can be responsible for a 100-kyr cycle. It raises therefore a question about how CO2 and ice sheet forcing combined with insolation changes affect the summer Asian monsoon system. If we look at our results in the light of (Su et al., 2025) outputs, we can hypothesize than orbital parameters impact is prevailing over the ice sheet one, but not over the greenhouse gas forcings.

Concerning the comparison with (Lyu et al., 2019) works, (Su et al., 2025) simulations reveal a significant 100-kyr period for the CO2 simulation in the most southern part of the domain, matching with our results with Orb_Ghg. This suggests that greenhouse gases are not negligible in explaining the Asian summer monsoon eccentricity-pace.

Finally, we would like to emphasize the fact that equilibrium simulations are obtained by resolving the physics of the climate system, which is not the case using an emulator, and they avoid the timing uncertainties in the climate response that are inevitable when the orbital-scale parameters are accelerated with a strong coefficient (x100 in (Su et al., 2025)). As such, the "snapshots" method presented in this study likely provides the most robust outputs.

(4) The authors use five proxy records to validate the model, with only one land-based speleothem record located near ~30°N. I understand that there are very few terrestrial records extending back 800,000 years. However, given the importance of the Asian monsoon domain—particularly its northern margin—I encourage the authors to include additional land-based records, even if they only cover a portion of the entire research period. Incorporating additional land-based records would provide more robust validation of the model simulations of monsoon variability.

A good point. We have included in the revised version of the manuscript six additional geological records to compare with model's outputs. Especially, three land-surface temperature reconstructions from loess records (Lu et al., 2019) (Lu et al., 2022), and also three sea surface temperature reconstruction Mg/Ca ratio recorded in the Arabian sea (Tarique et al., 2023), in the Bay of Bengal (Govil et al., 2011) and in the South China sea (Wei et al., 2007). These additional records are used to identified temperature bias in the model, while we clarified that we used the delta18O records to mainly test the variability of the simulations sets. These comparisons are seen on a revised version of Fig(3). Timeseries from the model show good agreement with the new temperature proxies included and

provide as such a more robust validation of the model's outputs. Thank you for suggesting this.

Minor comments:
Line 161-162: It is confusing for readers why the manuscript suddenly introduces the global wavelet spectrum and why it is necessary. Providing one or two sentences to clearly state its purpose and how it contributes to the overall argument would greatly improve clarity.

The purpose of including global spectrum to our wavelet is to show the different strengths of the significant periods for the 4 variables studied. We have more clearly introduced the purpose of this analysis on lines 202-203 now.

Please define "JJS" in figure caption of Fig. 2. Is "JJS" denote June-July-Augst" or "June-July-September"?

For Fig(2), Fig(S1) and Fig(S2) the legend has been changed to MJJAS (May, June, July, August and September) because we modified our months of study to these months (rather than JJAS).

Figures visualization and explanation are not clear enough for arguments.
Line 116-117: "for the strongest monsoons, the Orb set is overall cooler than the two other sets (Fig.(S1)) whereas we observe the opposite pattern for the weakest monsoons time periods." I find it difficult to discern the differences in surface temperature between stronger and weaker monsoon states from shading of Fig. S1 alone. The current presentation does not clearly support the authors' argument. I encourage the authors to add an additional row of subplots in Fig. S2 to directly illustrate the surface temperature differences between stronger and weaker monsoons for the three simulation sets.

Fig.(S1) and Fig.(S2) have been revised to match with the new domain chosen for the study (western arid part has been removed) and to improve their significance. In particular, calculations have been run again and are visible on the legend of the Fig. (S1). However, instead of classifying the weakest and strongest by looking at the mean summer monsoon precipitation (bigger or smaller than the global mean across the entire 800-kyr), we have chosen to define the weakest as the one with weaker summer rainfall than the 1rst quantile and the strongest as the one with stronger rainfall that the 3rd quantile (quantiles calculated using the entire time slice).

Fig.(S2) now includes another row to show the temperature difference between stronger and weaker monsoons for the four simulations sets.

Fig. 3 requires improvement. Overlaying the $\delta^{18}O$ records with the corresponding model outputs (SST, temperature, or precipitation) at the same locations would greatly help readers directly assess the similarities and differences between data and simulations. Presenting each time series in a separate subplot not only wastes space but also makes comparison more difficult.

Fig.3 has been modified to facilitate the comparison between model and geological data. Both proxies and corresponding model's outputs have been overlayed on a same plot.

Line 165-166: "In this respect, Fig.(S3) shows precipitation time series
166 for four sub-domains and reveals a similar decoupling…". Please add a map with a framing box to show the location of each sub-domain shown in Fig. S3.

The results of the former Fig.(S3) are now seen on Fig.(S5) with a clearer legend and a map (a) showing the sub-domains defined to compute the precipitation timeseries.

Line 81-82: "temperature" should be "temperature difference or gradient"?

Thank you for noticing the mistake. It has been corrected (line 85).

Line 133-134: "Wavelet analyses show significant power at precession timescales (≈21kyr) throughout the 800 kyr apart from around 400 kyr, for every variables studied". and Line 139 "There is also a clear 400 kyr signal present in simulated SSTs and precipitation over the ocean …". However, from Fig. 5, I do not see a "clear" 400-kyr cycle across all sets of simulations. In particular, no spectral peaks above the 95% confidence level are indicated in the 400-kyr band. Could you clarify how this argument is derived?

Thank you for raising this issue. This argument has been removed because indeed, apart from a very qualitative look at the timeseries seen on Fig.(4), nothing in our analysis is validating this argument. The timeseries are too short to test the hypothesis.

References:

Su, B., Sun, Y., & Zhou, M. (2025). Orbital-accelerated transient simulations of glacial-interglacial climate cycles for the last 800,000 years. Scientific Data, 12(1), 961.

Full references of the new records included in our analysis:

Mohd Tarique et al. "Enhanced CO2 Degassing From the Tropical Indian Ocean During Cold Climatic Events of the Last Glacial Cycle". In: Paleoceanography and Paleoclimatology 38.12 (Dec. 2023), e2022PA004570. issn: 2572-4517, 2572-4525. doi: 10.1029/2022PA004570. url: https://agupubs.onlinelibrary.wiley.com/doi/10.1029/2022PA004570 (visited on 10/22/2025).

Pawan Govil and Pothuri Divakar Naidu. "Variations of Indian monsoon precipitation during the last 32kyr reflected in the surface hydrography of the Western Bay of Bengal". In: Quaternary Science Reviews 30.27 (Dec. 2011), pp. 3871–3879. issn: 02773791. doi: 10 . 1016 / j . quascirev . 2011 . 10 . 004. url: https ://linkinghub.elsevier.com/retrieve/pii/S0277379111003180 (visited on 10/22/2025).

Gangjian Wei et al. "High-resolution sea surface temperature records derived from foraminiferal Mg/Ca ratios during the last 260 ka in the northern South China Sea". In: Palaeogeography, Palaeoclimatology, Palaeoecology 250.1 (June 2007), pp. 126–138. issn: 00310182. doi: 10.1016/j.palaeo.2007.03.005.url:https://linkinghub.elsevier.com/retrieve/pii/S0031018207001526 (visited on 10/22/2025).

Hongxuan Lu et al. “800-kyr land temperature variations modulated by vegetation changes on Chinese Loess Plateau”. In: Nature Communications 10.1 (Apr. 29, 2019), p. 1958. issn: 2041-1723. doi: 10.1038/s41467-019-09978-1. url: https://www.nature.com/articles/s41467-019-09978-1 (visited on 10/03/2025).

Hongxuan Lu et al. “Decoupled Land and Ocean Temperature Trends in the Early-Middle Pleistocene”.In: Geophysical Research Letters 49.17 (Sept. 16, 2022), e2022GL099520. issn: 0094-8276, 1944-8007. doi:10.1029/2022GL099520. url:https://agupubs.onlinelibrary.wiley.com/doi/10.1029/2022GL099520(visited on 10/01/2025)

REVIEWER COMMENTS

Reviewer #1 (Remarks to the Author):

In the revised manuscript, the authors have addressed most of my concerns arose in the first round of review. However, I still have several major concerns that need the authors to address and clarify.

Thank your for your meticulous review and your comments, which have helped improve our paper. We have modified the text and figures following your guidance.

First, the writing of this paper is poor and there are many mistakes in the expression. For instance, many sentences lack the subject and/or objects. I would suggest the authors to ask a polishing company to edit and polish their paper. Nonetheless, I will still point out some obvious issues. For instance, “summer Asian Monsoon” was used in many places of this paper, the correct form should be “Asian summer monsoon”.

We agree and have taken further care and attention. The manuscript has been carefully read by native English speakers to avoid mistakes and improve expression. We have also used the Language editing check from Springer Nature to help us spot any remaining errors.

Second, the captions/instructions of Tables and Figures are very short and not clear. For instance, “Figure 1: Study area with proxy location from: 1) [39] (Hulu cave) δ18O…”, you should write like this: “Figure 1: The study region and location of proxy records. (1) speleothem δ18O records from Hulu/Sanbao caves in southern China [39]”. In addition, your reference here is wrong. The right one is “Cheng, H. et al. The Asian monsoon over the past 640,000 years and ice age terminations. Nature 534, 640–646 (2016).” This is a composite cave δ18O record over the last 640 kyr, not just from Hulu cave. See the paper above for more details. Similarly, modify the captions of all Tables and Figures to provide sufficient information. I would suggest the authors to read papers published on Nature Communications to see how Tables and Figures are presented.

The titles and captions of figures and tables supporting the manuscript have been revised to include more detail and to be clearer. Especially, Fig.(1) caption has been modified to provide the necessary information and the reference of the Hulu/Sanbao record we used has been corrected. Thank you for spotting this mistake.

Third, based on Fig. 5 in the Supplementary file, the precipitation timeseries from southern west part (Indian subcontinent) show strong glacial-interglacial changes (100 kyr cycle) in the All forcing, Orb_Ghg, and Orb_Ice. If this is not a mistake in the labels made by the authors, it is an interesting finding that the Indian summer monsoon rainfall is dominated by glacial-interglacial changes (100 kyr cycle). Why not discuss it in more depth?

Thank you for your comment. The titles above each plot were wrong and where the summer monsoon rainfall is dominated by 100-kyr cycle is above South East Asia rather than above India. Each title of each subplot of the study have been carefully checked to avoid any other similar mistakes.

The 100-kyr cycle we observed in summer precipitation above South East Asia fully matches the signal we see in SST timeseries (Fig.(4)) because East Asian monsoon is particularly

sensitive to the thermal driver as described in Geen et al., 2020 and in Ding et al., 2005. This has been mentioned on lines 239 to 241.

Specific comments:

Thank you for pointing these writing mistakes. They have all been corrected. Fig.(6) has been modified as well to improve the readability.

Line 25: “These orbital parameters”, The orbital parameters.
Line 27: “feedback”, have feedback.
Line 28-29: “that can impact the Asian monsoon”, including the Asian monsoon.
Line 50-51: “Moreover, [23] considered not only……”, Moreover, Lyu et al. [23] considered not only……
Line 53: The same to line 50-51.
Line 54: the same.
Line 72: “summer Asian Monsoon”, Asian summer monsoon.
Line 96: “summer Indian monsoon”, Indian summer monsoon.
Line 103: “summer Asian Monsoon”, Asian summer monsoon.
Line 109: “SST”, define it in the first time. Sea surface temperature (SST).
Line 111: “in the basin in question”, in the same basin.
Line 229: “summer Asian Monsoon”, Asian summer monsoon.
Line 229: “South East sector”, East sector.
Line 255: “summer Asian Monsoon”, Asian summer monsoon.
Fig. 5 and Fig. 6: “mousson area from GPCC”, monsoon area from GPCC?
Fig. 6: Some graphs need to adjust their coordinates to make the results clear, for example, the four panels on the right and the two in the lower left.

<u>References</u>:

Geen, R., Bordoni, S., Battisti, D. S., & Hui, K. (2020). Monsoons, ITCZs, and the concept of the global monsoon. *Reviews of Geophysics*, 58, e2020RG000700. **https://doi.org/10.1029/2020RG000700**

Yihui, D., Chan, J. The East Asian summer monsoon: an overview. *Meteorol. Atmos. Phys.* **89**, 117–142 (2005). https://doi.org/10.1007/s00703-005-0125-z

Reviewer #2 (Remarks to the Author):

Summary:

Millot-Weil et al. have revised their manuscript to include some additional analysis of monsoon dynamics in the simulations and discussion in the context of previous work, although their main approach and conclusions remain the same as the prior version of the manuscript. I appreciate that the authors have acknowledged more recent definitions of monsoon circulation and added more in-depth explanation of mechanisms as noted in my previous review. However, I still have some major concerns regarding the analytical framework, communication of the results, and scientific impact of the conclusions relative to previous work in the field, which unfortunately make me question whether this revised manuscript is appropriate for this journal. Please see more detailed comments below.

Thank you very much for your thorough assessment and time taken to review our manuscript, your comments have been particularly helpful in reframing some of our arguments and further implications. We have also made several improvements now to both the analysis, framing and communication of the work as well. We very much appreciate the time and support you have shown in reviewing our manuscript.

Minor Comments:

• Many instances of “precipitations”, and other plural words that should be singular “variables” (line 178), “simulations” (line 179)
• Fig S1: No labels on the colorbar
• Many qualitative lines that would benefit from quantitative comparison to make the argument more clear (e.g., Lines 155-158, 164-165)
• Line 161: Switch “simulated” to “resolved”
• Using the term “observations” too much and for different uses, you should use one word more consistently (e.g., line 180 observations is used to described results from Fig. 4 and then also used on line 317 to describe proxy-observations)
• Many sentences where the grammar and sentence structure make it difficult to understand the intended meaning of it (lines 112-114, line 149, lines 202-203)

Thank you for your comment on the language. The specific mistakes mentioned have been corrected and we made several improvement to the legibility of the text as well as making use of the language editing software from Springer Nature to help us spot any remaining errors.

Major Comments:

• Abstract: this does not read with a strong statement of significance for the paper. We do learn that precession is the most important driver of monsoon pacing, but this has been explained by previous studies (e.g., lines 44-55) and it is not clear why the approach of this study is sufficiently novel to warrant publication in this journal.

This is a very fair point. Our original abstract was perhaps to forensic. The abstract has been majorly revised to better convey the novelty of the method, results and implications.

• Lines 81-104: I appreciate that you have acknowledged the definition of the monsoon as a variation of atmospheric circulation and location of the ITCZ, rather than a land-sea breeze. However, I understand that you are largely refuting this definition without clear justification. Your new plots of ITCZ location and Hadley circulation (Figs. S3 and S4) show that the ITCZ

location indeed shifts poleward during stronger monsoons/stronger summer precipitation, particularly in the East Asian region. This appears to support the ITCZ-based monsoon definition, but you have not included corresponding plots to show the distribution of precipitation and moist static energy which would be needed to evaluate the ITCZ-based definition of a monsoon. Also, on lines 93-95, you claim that "The ITCZ… never attains further than ~15N above the Indian sector, and ~35N above East Asia". Doesn't this statement only apply to the weakest summer precipitation cases (top row) of Fig. S3 and S4? I am not sure how these plots refute the ITCZ-based monsoon definition. So together with Fig. 2, I am not convinced of your final interpretation that "in the model and at orbital timescale, the traditional comprehension of the summer Asian monsoon is still accurate".

Thank you for raising these concerns and suggesting new analysis. We agree that our statement was not clear enough. We have fully revised the first part of the results (lines 82 to 156) to improve the clarity of the argument and to better fit within the most up-to-date description and how to diagnose tropical vs. extratropical monsoon circulation.

Notably, we take advantage of the review from Geen et al., 2020 following your suggestion. They highlight in particular two different regimes of convergence zone among the various monsoon systems. The first one can be described as an ITCZ (eddy driven) dominated monsoon system, whereby if the convergence zone (and the main loci of precipitation) stay between the equator and ~10-15 degrees North. The second one is called "monsoon regime", whereby the convergence zone (and precipitation) is found around 20-40N and is primarily a near-angular-momentum-conserving regime, not eddy driven like the ITCZ. Today, this can be seen by the northward progression of the quasi-stationary subtropical Baiu-Meiyu front through the summer.

To explore which regime is described by the model, we have added the distribution of precipitation (Fig. (S2)) for the weakest and the strongest monsoons. In addition, the subcloud moist static energy profiles (MSE) (Fig.(S4-S5-S6 (b))) have been included to the Hadley circulations plots which show the position of the convergence zone. Moreover, we have decided to provide an additional figure showing the Hadley circulation and the MSE profile that covers the large domain we focus on, rather than showing only the two subsystems (SASM & EASM). These extra analysis show that the model generally falls within the "monsoon regime" described in Geen et al., 2020 (convergence zone and precipitation exceeding 10 degree N), apart for the weakest monsoons above the Indian domain that can be explained by ITCZ shift only. While more southern regions of the Asian monsoon region will be affected by smaller poleward movements of the ITCZ location, the extratropical monsoon regime controls its maximum poleward extent through the season. Both are changing in tandem between orbital states due to both being thermal driven circulation.

Geen et al., 2020 as well as Ding et al., 2005 and Walker et al., 2016 show that a "monsoon-like" development of the convergence zone is thermal driven. This is what we show on Fig.(2), where the correlation between high precipitation and thermal contrast between the land and the sea is very strong.

• Results & Discussion: The lack of mechanistic explanation for the relative roles of CO_2, orbital forcing, and land ice on monsoon variability noted by my previous review and other reviewers is a hinderance to the scientific impact of this study. This is an additional reason why I do not recommend publication in this journal.

By refining the definition of monsoon dynamics and incorporating additional analyses (notably the MSE profiles which align with the precipitation distribution) we better highlight the role of greenhouse gases in Asian monsoon variability. Because thermal forcing is critical for extratropical monsoon development (or the “monsoon-like convergence zone”) greenhouse gases, by modulating thermal gradients and SST, act as a direct driver of summer monsoon intensity across the broad Asian domain. This has been more clearly mentioned on lines 168-169, 175-176, 181-184, and 345.

• Regarding my comment in the initial review on the paleo-calendar effect, I understand why the authors chose to not incorporate it as it would present a substantial amount of work to calendar adjust all original monthly simulation files. However, I do not believe the statement that “by taking an average over several months, [the authors] smooth the impact of any calendar shift.” Primarily this is because there is a temporally varying spatial imprint of the calendar effect and it is not just a matter of varying month-length anomalies of a few days. I would like to see an acknowledgement of the impact the paleo-calendar effect could have on their results.

Thank you for raising your doubts about the calendar effect.

To better evaluate the potential impact of the paleo-calendar effect on our results, we took advantage of the data provided by (Bartlein & Shafer, 2019), especially the ones they used to produce their Fig. 11 and Fig.13. These figures show respectively the calendar effect on surface temperature and on precipitation (difference between model outputs adjusted to paleo-calendar effect and a model that does not include the effect), for the 12 month of the year and for 4 distinct time periods (6ka, 97ka, 127 ka and 116ka). We have selected the months our results lie on: May to September, that we have averaged. Then we selected our region of interest : lat : 0/50 N, lon : 60/130 E. The figures obtained for both surface temperature and precipitation are the following:

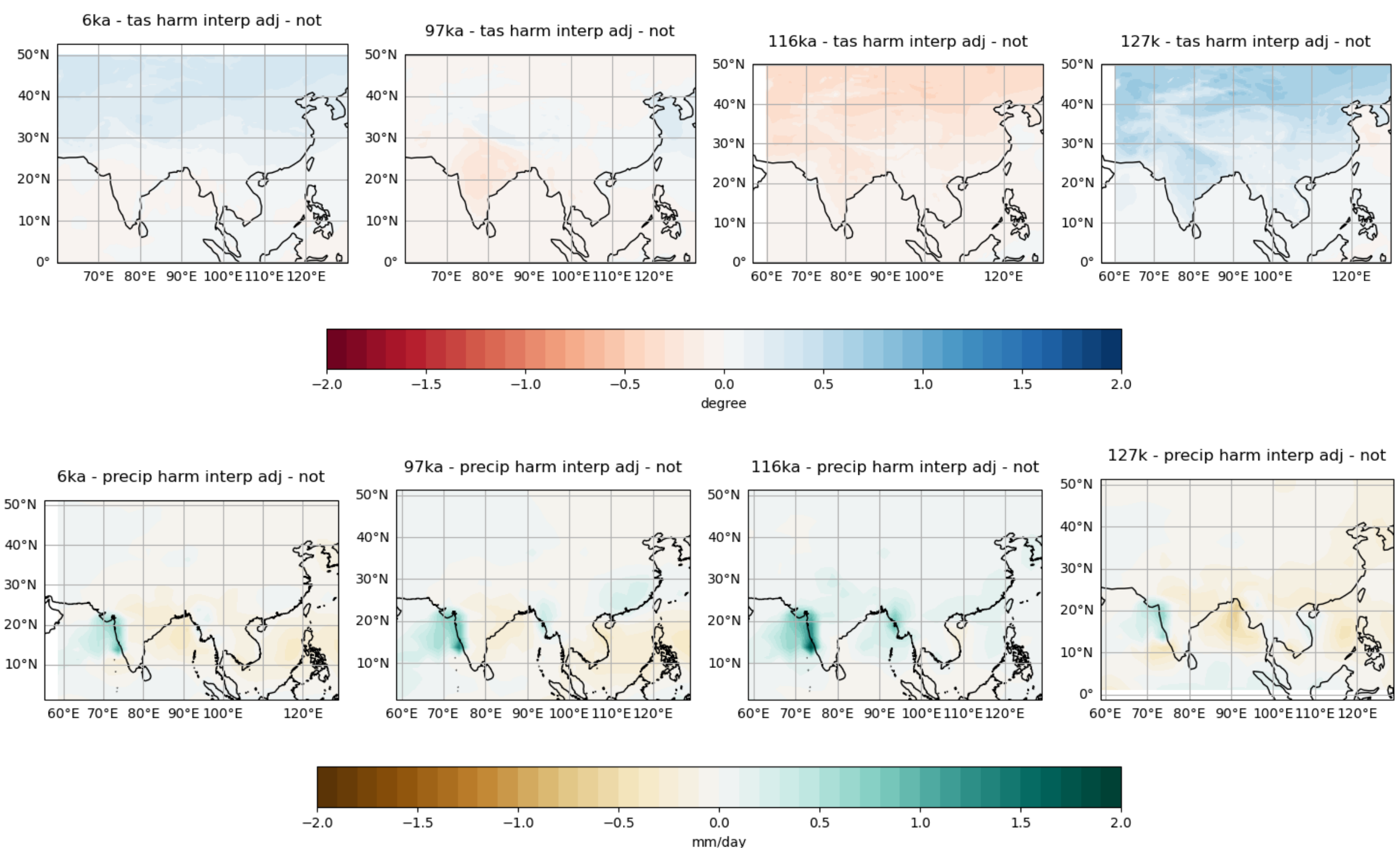

Fig: CFSAR (calendar adjusted values via harmonic interpolation) – (not adjusted) for both surface temperature (top) and precipitation (bottom), averaged over the summer months (May to September). Outputs from (Bartlein & Shafer, 2019).

These figures show that the impact on temperature is relatively small (surface temperature anomalies are within a range of +/-1.0 ℃) and on precipitation (+/-0.5 mm/day, with a slightly larger range on the Eastern Arabian sea). These values are below both observed and model interannual variability (Fig. S3, range of 8 degrees or 8 mm/day). As such, taking into account the paleo-calendar effect in our study, that focus on low latitudes and on an average over several months would have had only a minimal impact. However, we agree that this does need to still be highlighted, we have now clearly stated in the paper on lines 408-411.

References:

Geen, R., Bordoni, S., Battisti, D. S., & Hui, K. (2020). Monsoons, ITCZs, and the concept of the global monsoon. *Reviews of Geophysics*, 58, e2020RG000700. **https://doi.org/10.1029/2020RG000700**

Yihui, Ding & Chan, Johnny. (2005). The East Asian summer monsoon: An overview. Meteorology and Atmospheric Physics. 89. 117-142. 10.1007/s00703-005-0125-z.

Walker, J. M. and S. Bordoni (2016), Onset and withdrawal of the large-scale South Asian monsoon: A dynamical definition using change point detection, *Geophys. Res. Lett.*, 43, 11,815–11,822, doi:10.1002/2016GL071026.

Bartlein, P. J. and Shafer, S. L.: Paleo calendar-effect adjustments in time-slice and transient climate-model simulations (PaleoCalAdjust v1.0): impact and strategies for data analysis, Geosci. Model Dev., 12, 3889–3913, https://doi.org/10.5194/gmd-12-3889-2019, 2019.

REVIEWER COMMENTS

Reviewer #1 (Remarks to the Author):

The paper of Jeanne Millot-Weil et al. has been greatly improved based on the comments of reviewers. However, there are still a lot of editorial mistakes and some other minor errors in this version. For the editorial mistakes, it needs the publishing editor to comment and I just list some of them in below.
The major contribution of this paper is that it provides a dataset of climate simulation over the past 800 kyr. The authors also indicate where the data are available. However, I find it very difficult to access the model data based on the link provided by the authors, which hindered the validation of this paper. It is also not conducive to the spread and development of science. Therefore, I strongly suggest the authors to share the model data in ways that are easy to access, such as zenodo or figshare.

Thank you for your thorough review. We have now corrected all the editorial mistakes pointed below. Concerning where the data should be deposited, we are not opposed to use Zenodo or Figshare. However, the size and quantity of simulations amount for several TBs, generally exceeding the capacity of the mentioned depositories. This is why the repository available at https://www.paleo.bristol.ac.uk/ummodel/scripts/papers/ (please refer to the pdf called “Using_BRIDGE_webpages.pdf” on how to operate the system on that page) has been implemented about 20 years ago, to ensure that large model output datasets remain accessible. If the data used in this study remain difficult to access, we would be pleased to discuss alternative repository which can handle such large dataset with the editor.

The direct link to the datasets themselves can be found here:
https://www.paleo.bristol.ac.uk/ummodel/scripts/papers/Millot-Weil_et_al_2025.html

Following the links called “Detailed List of Runs” reveals each simulations as well. Or clicking on the link “You can have make you own analysis and plots by going here” also allows visual inspection of the data for some standard plots and timeseries, as well as the ability to do more detailed analysis via the “Analysis” Button. And of course, all data requests via email as is often the case will also be undertaken.

Figure 1:
“1) speleothem” should be “1) Speleothem”.
“2) benthic” should be “2) Benthic”.
“4) δ18O sea water”, 4) Sea water δ18O.
6) foraminiferal, 6) Foraminiferal.
7) foraminiferal, 7) Foraminiferal.
Line 37: “Since then”, revise to “However”
Line 42: “greenhouse gases increasing”, greenhouse gases in increasing.
Line 53: “pCO2”, define it or revise to “CO2 concentration“。”
Line 53-54: “East Asian summer precipitation”, East Asian summer monsoon precipitation.
Line 54: delete “from Chinese loess deposit”.
Line 56-57: “play a primary role on East Asian precipitation intensity”, play a primary role in regulating East Asian monsoon precipitation intensity.
Line 57-60: “Lyu et al. [24] however, considered not only the East Asian monsoon intensity but also its pace over the last 800-kyr and showed a direct impact of ice sheet and precession cycle but this used a Gaussian emulator based on previous HadCM3 simulations.” Recently, Lyu et al. [24] considered not only the East Asian monsoon intensity

but also its pace over the last 800-kyr and showed a direct impact of ice sheet and precession cycle. However, this study used a Gaussian emulator, not direct simulations, based on previous HadCM3 simulations.
Line 63: “an emulator based approach”, an emulator-based approach.
Line 79: “pCO2”, CO2 concentration.
Line 82: “Quaternary Asian summer Monsoon”, Quaternary Asian summer monsoon.
Line 84: “out performing”, outperforming.
Line 125-126: “red dots - no2, no3, no4, no5”, what this means?
Line 127: “pink dot - no1”, ???
Line 130: “yellow dots - no7, no6, no8”, ???
Line 131: “yellow dots - no7, no6, no8”, ???
Line 132: “Fig.(3) (a)”, Fig. 3a.
Line 133: “Fig.(3) (b)”, Fig. 3b.
Line 135: “Tab.(2)”, Tab. 2.
Line 146: “Fig.(3) (b)”, Fig. 3b.
Line 146-147: “The average bias between model and proxy SSTs is +1.73 oC and -1.71 oC respectively, in average for the LST”, sick sentence. The average bias between model and proxy SSTs is +1.73 ℃, and the average bias between model and proxy LST is -1.71 ℃?
Line 149: “(Tab.(2))”, Tab. 2.
Line 152-153: “Correlations were not significant according to the p value.”, the correlations between what?
Line 161: “Fig.(S1)”, Fig. S1.
Line 162: “Fig.(S1)”, Fig. S1.
Line 163: “Fig.(S5) & (S6)”, Fig. S5 & S6.
Line 164: “Fig.(S2) & (S3)”, Fig. S2 & S3.
Line 174: “Fig.(S3)”, see above.
Line 179: “Fig. (4)”, “Fig. (1)”
Line 193: “Figs. (S4–S6)”, Figs. S4–S6.
Line 201: “Fig. (1)”, Fig. 1.
Line 202: “Fig.(5)”
Line 206: “Fig.(4)”
Line 207: “Fig.(1)”
Line 209: “Fig.(5)”
Line 218: “Fig.(5)”
Line 224: “Fig.(2)”
Line 229: “Fig.(6)”
Line 230: “Fig.(5)”, “Fig.(6)”
Line 232: “Fig.(1)”
Line 235-236: “Fig.(S4 & S5”
Line 236: Fig.(S7)
Line 238: “South East summer Asian monsoon domain”, southeastern Asian summer monsoon domain.
Line 251-252: “Fig.(S1), (S2) and (S3)”
Line 264: “east Asian summer monsoon”, East Asian summer monsoon.
Line 272: “Fig.(7)”, “Fig.(5)”
Line 273: “Fig.(7)”
Line 280: “GHG’s”, greenhouse gases
Line 281: “at 100 kyr. Thus confirming”, at 100 kyr, thus confirming
Line 283: “GHG”, greenhouse gases, or define it in the first time you use it.
Line 289: “Northern hemisphere”, Northern Hemisphere
Line 304: “Fig.(8)”

Line 306: “Fig.(8)”
Line 307: “A persistent 100-kyr signal emerges in the Northern part of the East Asian region (15-20◦N)”, revise “Northern part” to “southern part”.
Line 308: “Fig.(8)”
Line 315: “around 400kyr”, at around 400kyr
Line 322: “Fig.(6) and Fig.(S7)”
Line 330: “Fig.(2)”
Line 331: “Tab. (2)”
Line 356-358: “These results also motivate further process-based analysis to better quantify the contribution of orbital-scale forcings shape atmosphere dynamics that underpin monsoon behaviour”, revise it for better understanding.

Reviewer #2 (Remarks to the Author):

I appreciate the revisions that Millot-Weil et al. have made to their manuscript. The current version of the manuscript has stronger scientific justification and writing. They have broadened the definition of a monsoon and investigated it more thoroughly, corrected grammatical errors, and evaluated the potential impact of the paleo-calendar effect on their results. Improvements to the text also provide a clearer explanation of the advancement in methodology that has been made compared to previous studies on similar topics (e.g., Lyu et al. [24], Lu et al.[25], Su et al., [46]).

Thank you for your comments on our latest version of manuscript.

Major Comments

- One of the main conclusions of the paper is a “precipitation over the entire region is strongly correlated with the land-ocean temperature gradient” (line 256-257), this does not provide an improved mechanistic understanding of monsoon drivers, which is possible to more thoroughly investigate with full-complexity climate model simulations. I believe that the additional analyses you have done in the supplement (Figure S2, S4, S5, S6) help demonstrate that the spatial extent and drivers of the monsoon is more complicated than land-sea thermal contrast.

The correlation showed on Figure 2 comes from the full-complexity climate model outputs and show a strong correlation between the land-sea thermal contrast and precipitation. While we agree that monsoon behaviour is complex and subtleties within an individual time period exist, the overall trend through the 800,000 years sequence shows a strong correlation with the land-ocean temperature gradient, we cannot disregard this result which needs to be stated in our manuscript. Additionally, we do not believe that the paper affirmed anywhere that the mechanisms behind the Asian summer monsoon only account for a land-sea thermal contrast, but show that it is a strong driver of some of the dynamics of the Asian monsoon as stated in (Geen et al., 2020). It is not a purely eddy-driven Hadley-cell excursion as has often been stated, in particular in driving the poleward extent of the monsoon system.

However, to further confirm the thermal forcing role in the Asian summer monsoon precipitation, we have included additional plots showing the relationship between diabatic

heating (summer latent heat flux; impacting monsoon intensification (Diaz et al., 2019) and spring sensible heat flux; initiating the monsoon circulation (Li et al., 2026) (Wu et al., 2009)) above the Tibetan Plateau and summer precipitation and land-sea thermal contrast (Figure 1 in this document and Fig.S7 in the manuscript). We found strong correlation between summer latent heat flux (lhf) and monsoon precipitation (Person's correlation test giving a correlation coefficient of 0.96 for a p_value = 2.81e123) and between summer lhf and summer land-sea thermal contrast (correlation coefficient of 0.95 for p_value = 2.09e-111). Similarly, spring uplifted sensible heat flux (shf) equally correlates with monsoon precipitation and land-sea thermal contrast (correlation coefficient of 0.63 for p_value = 4.55e-26 and correlation coefficient of 0.63 for p_value = 9.63e-26 respectively). These additional results confirm the impact of the thermal forcing in the Asian summer monsoon and have been included on lines 114-121.

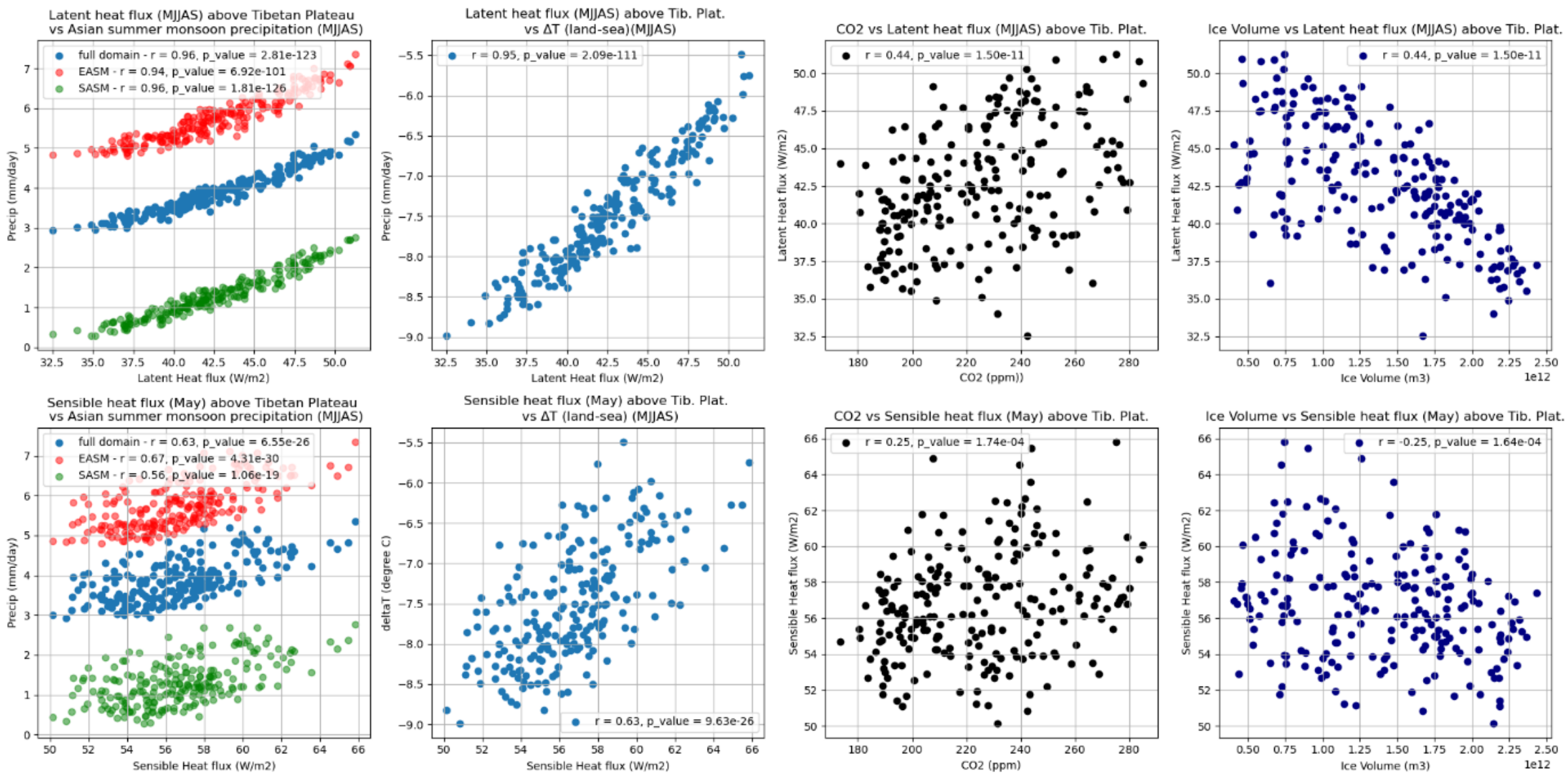

Figure 1: **Relationships between Tibetan Plateau heat fluxes and Asian summer monsoon variability in All_forcings simulations.** Top and bottom rows show latent and sensible heat fluxes, respectively, against monsoon precipitation, land–sea thermal contrast, the CO2 forcing, and the ice sheet forcing.

Moreover, similar diagnostics have been performed with the three other simulation sets (OrbGhg, OrbIce and Orb; Fig.2-3-4 in this document and Fig.S8-S9-S10 of the manuscript). They reveal the important relationship between greenhouse gases variations and spring shf whereas ice sheet variations show more impact on lhf rather than greenhouse gases. This additional result further supports the pacing role of greenhouse gases (through its impact on the monsoon initiator: spring shf), whereas ice sheets only impact its intensity (through amplifying: summer lhf). These results have been thoroughly described on lines: 185-198 and included in the conclusion on lines: 367 and 371.

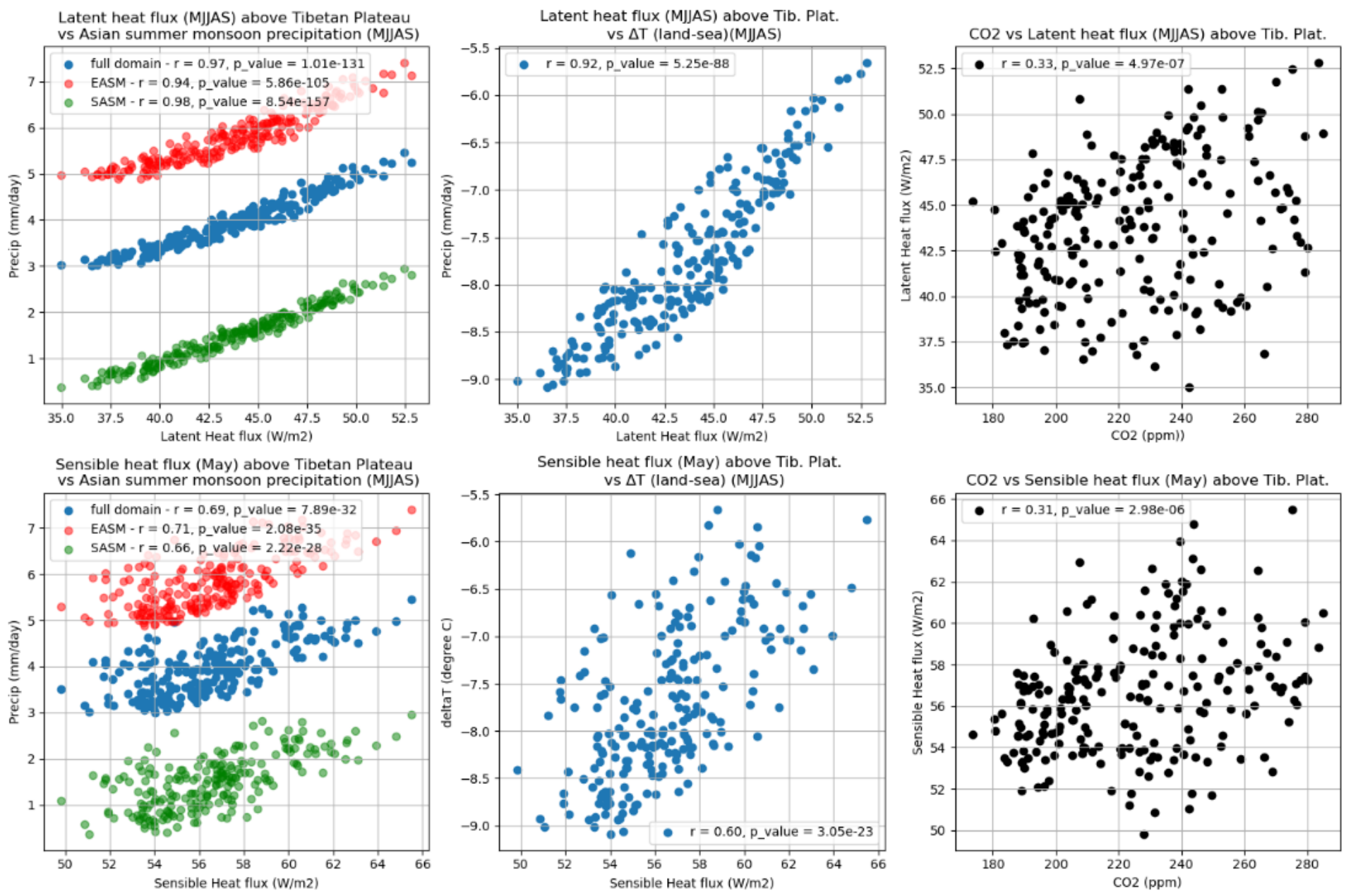

Figure 2: **Relationships between Tibetan Plateau heat fluxes and Asian summer monsoon variability OrbGhg simulations.** Top and bottom rows show latent and sensible heat fluxes, respectively, against monsoon precipitation, land–sea thermal contrast and the CO2 forcing.

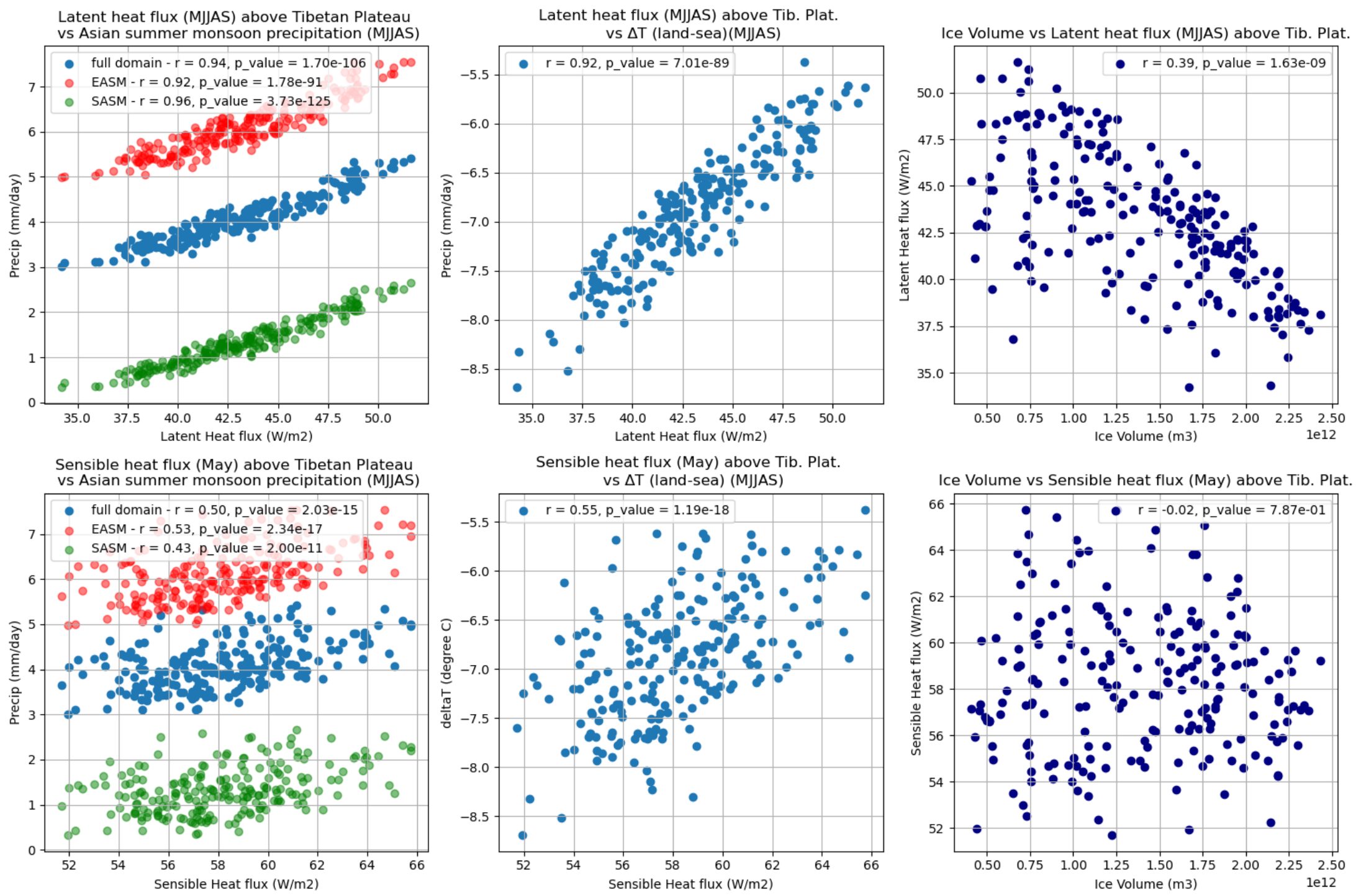

Figure 3: **Relationships between Tibetan Plateau heat fluxes and Asian summer monsoon variability OrbIce simulations.** Top and bottom rows show latent and sensible

heat fluxes, respectively, against monsoon precipitation, land–sea thermal contrast and the ice sheet forcing.

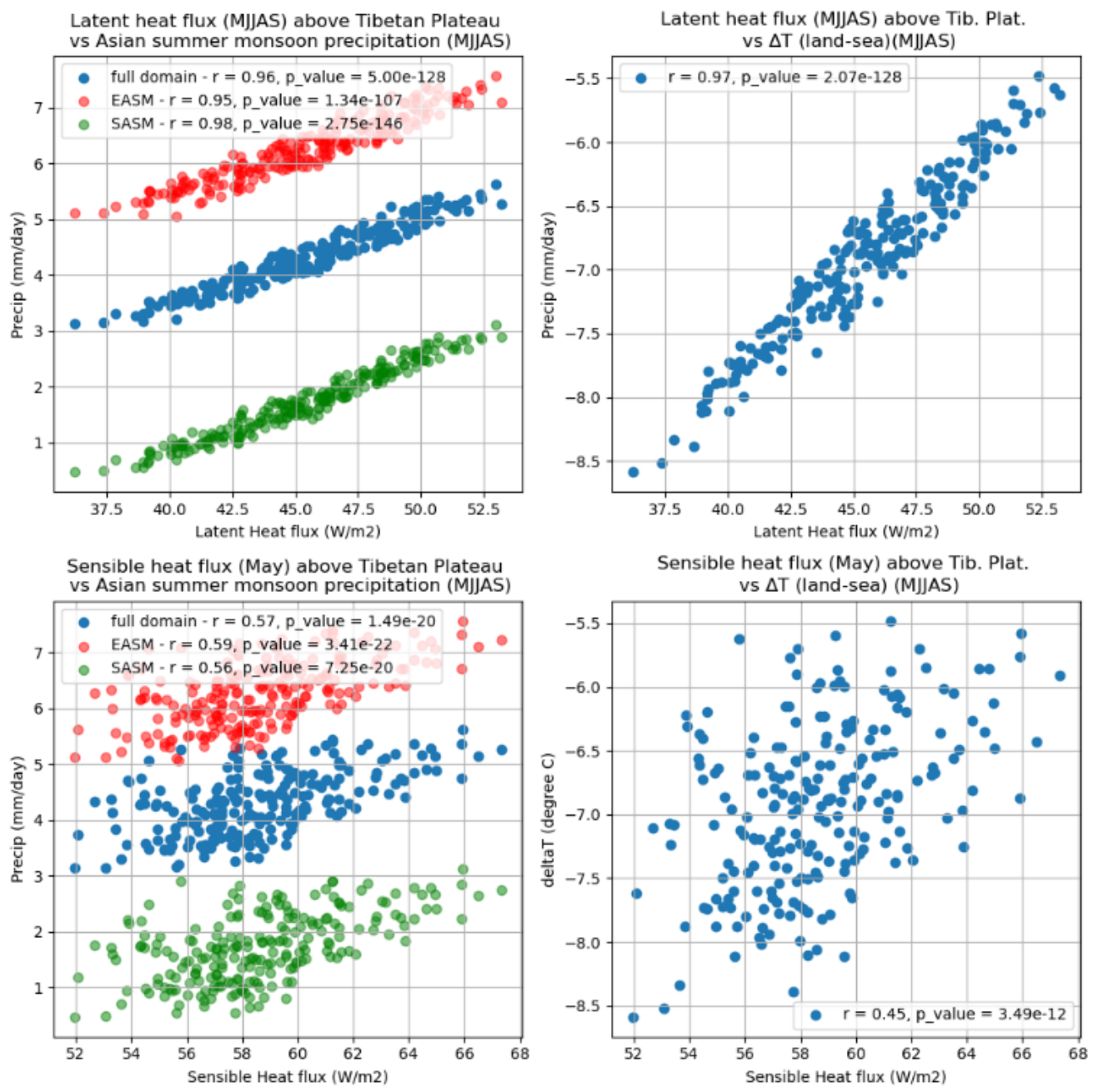

Figure 4: **Relationships between Tibetan Plateau heat fluxes and Asian summer monsoon variability OrbOnly simulations.** Top and bottom rows show latent and sensible heat fluxes, respectively, against monsoon precipitation and land–sea thermal contrast.

- One additional main finding of the study is that “Spatial heterogeneity in monsoon dynamics reconciles conflicting proxy records, linking discrepancies to regional characteristics and different sensitivities to imposed forcings” (lines 9-11). This implies that you have improved our understanding of mechanisms driving complex signals archived proxy records in the region. Instead, my understanding is that you have shown that the “Pearson’s correlation (Tab.(2)) between the geological data and model show significant correlation between proxy and model temperatures, although higher coefficients are found for the SST rather than for LST” (lines 148-150). So you do not provide a mechanistic explanation of regionally conflicting proxy records, which would drive higher impact of the study.

This is a very fair point. The formulation here is misleading. We have modified this sentence (lines 9-10) to better convey the results of the paper.

- You explain that “our analysis encompasses a wide domain, whereas the Asian summer monsoon comprises two distinct subsystems (South Asian sector and East Asian sector) which differ in their dynamics and are often studied separately” (lines 257-259) and ultimately I believe this limits the impact of the study because the results more general, rather than providing a detailed mechanistic understanding that could come from a more constrained, regional analysis.

We understand this point of view as studying separately the two sub-systems of the Asian summer monsoon will be the focus of future work. However, in the first order, the East Asian summer monsoon and the South Asian summer monsoon equally correlate to the sensible and latent heat fluxes that drive the Asian summer monsoon system (Figures 1-2-3-4 in this document or Figures S7-S8-S9-S10 of the manuscript). As such, at such a long timescale, studying a broad domain still gives valuable outcomes. We have clarified this on lines 121-123.

Minor comment
Line 227: forcing is misspelled

This typo has been corrected. Thank you for spotting it.

References:

Geen, R., Bordoni, S., Battisti, D. S., & Hui, K. (2020). Monsoons, ITCZs, and the concept of the global monsoon. *Reviews of Geophysics*, 58, e2020RG000700. **https://doi.org/10.1029/2020RG000700**

Li, J., Geen, R., Song, Z., Mao, J., Song, Y., Zhao, Y., et al. (2026). Impacts of surface sensible heating over the Tibetan Plateau on cloud fraction and surface radiation budget in surrounding Asia. Journal of Geophysical Research: Atmospheres, 131, e2025JD045385. **https://doi.org/10.1029/2025JD045385**

Wu, G. X., Liu, Y., Zhu, X., Li, W., Ren, R., Duan, A., and Liang, X.: Multi-scale forcing and the formation of subtropical desert and monsoon, Ann. Geophys., 27, 3631–3644, https://doi.org/10.5194/angeo-27-3631-2009, 2009.

REVIEWER COMMENTS

Reviewer #1 (Remarks to the Author):

Thank the authors for carefully revise their paper again. I have reviewed this paper three times previously, and I do not want to comment on the grammar anymore. For my major concern, the data are still difficult to access. Although the authors replied that "the size and quantity of simulations amount for several TBs", the size of one month of simulation is only 33 KB. For monthly precipitation data, there are 100 years for one snapshot, 12 months per year, and 219 snapshots in total, the size is only about 8 GB. When adding temperature data, the size is only ~16 GB. This is not too large to be stored on public websites such as zenodo or figshare. In the website provided by the authors, one need to repeat 12*219 = 2628 times to get the data of one climate variable, this is really a big and unnecessary work. In summary, the authors should make their data easy to access, even only the long-term monthly mean of each snapshot is provided. This is where the value of this paper lies.

Thank you for mentioning the tedium of accessing the model outputs.

We have now extracted the outputs used in the analyses presented in the paper. Specifically, May and June–September mean surface temperature and precipitation fields for each simulation set are stored in a single file, while sea surface temperature is stored separately because the atmosphere and ocean components have different spatial resolutions. All fields are provided on the global domain, and the time dimension of each file corresponds to the number of snapshots in the simulation set (219 time steps, with each time step representing one equilibrium snapshot).

These files are now available on zenodo (https://doi.org/10.5281/zenodo.21310840) along with a README text file to describe them, as well as the more comprehensive set of model data for this suite of simulations on the BRIDGE depository website.

Reviewer #2 (Remarks to the Author):

Thank you to Millot-Weil et al. for providing a detailed analysis of the land-sea thermal contrast and its relationship with seasonal heat fluxes across the different simulations. The authors have reasonably addressed my comments. I do not find the simulation data to be accessible through the BRIDGE web pages, as the webpages appear to be only summary statistics/plots and the raw simulation output is archived to tape. Perhaps this is reasonable given the size of the dataset, but I am unable to test the scripts associated with the analysis.

Reviewer #2 (Remarks on code availability):

I am unable to run the code because the simulation output is not accessible.

Thank you for sharing the difficulty to access the data we used in the paper. To facilitate the access, we have extracted to specific outputs used in the analysis (e.g. surface temperature, precipitation and sea surface temperature in May and June to September for each simulation set) to make them available on the public repository zenodo.
https://doi.org/10.5281/zenodo.21310840

Reviewer #4 (Remarks to the Author):

The orbital forcing of Asian monsoon evolution is a key research topic in Quaternary paleoclimatology and Milankovitch theory. Using the full-complexity HadCM3B model, the authors performed high-resolution snapshot simulations of paleoclimatic changes over the past 800 ka, quantified the impacts of distinct forcings, and re-evaluated the respective roles of orbital eccentricity and atmospheric CO_2 in modulating the Asian summer monsoon (ASM). This study presents solid paleoclimate modeling work and puts forward meaningful hypotheses regarding the dynamical mechanisms governing ASM variations. Overall, the authors have adequately addressed the previous reviewer comments and implemented corresponding revisions throughout the manuscript. Below are two major suggestions and one minor discussion point for the authors' reference.

Thank you for your thorough review. We very much appreciate the quick turnaround for this.

First, both the title and main conclusions indicate that precession and greenhouse gas variability jointly dominate the long-term evolutionary pacing of the ASM. However, the abstract only highlights the dominant control of greenhouse gases on the 100-kyr ASM periodicity while neglecting the contribution of precession. This inconsistency renders the abstract incomplete and inconsistent with the main findings of the study, and corresponding revisions are therefore recommended.

The abstract was indeed a little misleading. We have modified it to better convey the main results of the manuscript by mentioning the direct impact of precession on the Asian summer monsoon variability on lines 8-9.

Second, the statement in Lines 247-248—"and the significant 100 kyr power in both land and ocean precipitation is reduced or removed when ice sheet forcing is included"—is insufficiently objective and requires revision. Notably, the 100-kyr spectral power of land precipitation exhibits no statistical significance even in simulations excluding ice sheet forcing, which contradicts the above description.

Thank you for spotting this mistake. The sentence has been revised and now only mentioned ocean precipitation, which shows a statistically significant 100-kyr power, not the precipitation over the land (see lines 248-249).

In Lines 304-318, the authors attribute the discrepancies between their results and previous studies to differences in simulation strategies, and acknowledge that snapshot simulations may induce biases in the representation of deep-ocean circulation. It is well established that changes in Northern Hemisphere high-latitude insolation and ice sheet exert profound impacts on the Atlantic Meridional Overturning Circulation (AMOC). Substantial evidence from both orbital-scale paleoclimatic reconstructions and modern simulations suggests a tight linkage between AMOC variability and Asian monsoon changes (Cheng et al., 2016; Dong et al., 2025). Given that the model may inadequately resolve deep-ocean circulation, it is worth discussing whether such model limitations account for the absence of long-period cyclicity in the simulated ice-volume and orbital-forcing responses. This is merely an open scientific question for academic discussion and does not require mandatory revisions to the current manuscript.

This is a very interesting remark, and something we have also discussed at length together. In particular whether such simulations can or should be in equilibrium given the time lengths required for the surface, middle and the deep ocean to respond to surface forcings. i.e. is the deep ocean still responding to past forcings rather than current forcings in a given time period and what impact should that have on surface and teleconnected processes. It is a question we would like to directly address in the near future through a more constrained transient simulation to compare the snapshot against with the same setup.

Thank you for also mentioning the link between AMOC and Asian monsoon. We agree that both accelerated transient simulation and snapshot simulations will have limitations in representing deep ocean circulation, and its teleconnected impact on the Asian monsoon. Whether this has a large impact on the mean state of the monsoon or its interannual variability (or both) is also an open question. Only a non-accelerated transient simulation could adequately represent the linkage between AMOC and the Asian summer monsoon, which remain difficult to carry because of the high computational resources it needs and outside the scope of this paper.

It is important to note that the biases are different between both methodologies. In particular, (Liu et al., 2025) has shown that accelerating the orbital forcings by only a factor of 2 can substantially affect the AMOC response. On the other hand, our snapshot simulations do not have a fully equilibrated ocean, and have no memory of past climate states. Nevertheless, because each snapshot has been run independently, biases remain confined to individual simulations rather than accumulating throughout the time series, unlike in transient simulations.

These remarks have been included in the manuscript on lines 320 to 330.

Ref:

Liu, H., Liu, Y., Lisiecki, L., Gao, Y.,Zhang, M., & Lin, Q. (2025). Collapse ofthe Atlantic meridional ocean circulationinduced by precession: Sensitivity toorbital acceleration. Geophysical ResearchLetters, 52, e2025GL115941 **https://doi.org/10.1029/2025GL115941Digital Object Identifier (DOI)**

Overall, the manuscript is well-organized and provides valuable insights into Asian monsoon dynamics. I recommend publication pending minor revisions as suggested above.

Ref:
Cheng, H.*, Edwards, R.L.*, Sinha, A., Spötl, C., Yi, L., Chen, S.T., Kelly, M., Kathayat, G., Wang, X.F., Li, X.L., Kong, X.G., Wang, Y.J., Ning, Y.F., Zhang, H.W., The Asian monsoon over the past 640,000 years and ice age terminations, Nature, 534(7609)(2016), 640-646.
Dong, X.Y.*, Chen, R.S., Niu, X.W., Hummel, C., Kathayat, G., Liu, Y.T., Zhao, J.Y., Zhang, H.W., Shi, Z.G., Zhang, X., Yang, Y., Cheng, H.*, Early warning signals for Asian summer monsoon tipping and implications for future monsoon changes, The Innovation Geoscience, 3(4)(2025), 100158-1-100158-9.
