# Supplementary material for: Author Correction: Asian summer monsoon orbital variability directly paced by CO2 and precession, not eccentricity
Source: Nat Commun. 2026 Nov 4;17:11412. doi: 10.1038/s41467-026-78966-z (PMC13737005; doi:10.1038/s41467-026-78966-z)
Supplement: Supplementary file 1 [file 41467_2026_78966_MOESM1_ESM.pdf]

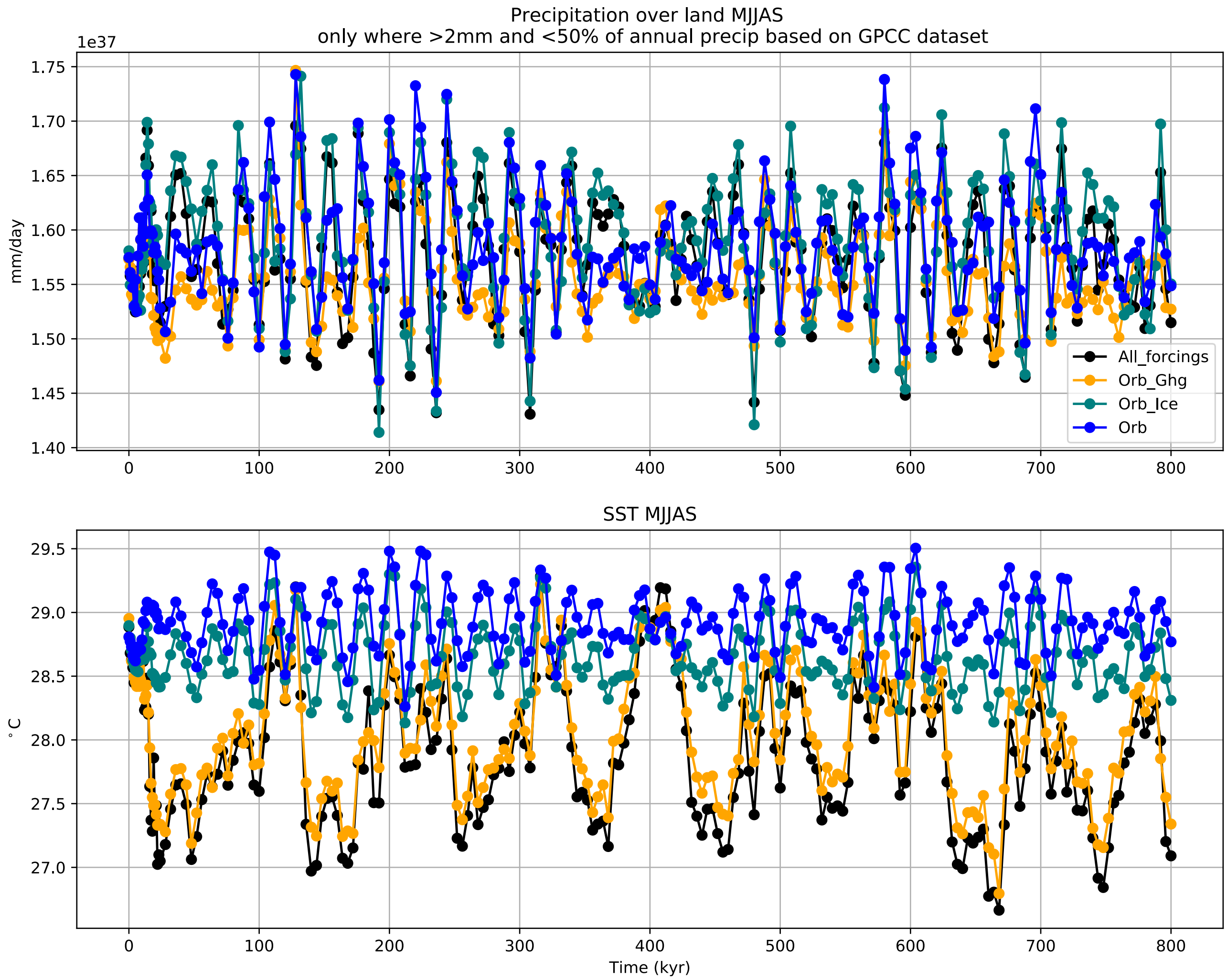

Fig. 4 | Summer monsoon Precipitation and sea surface temperature (SST) timeseries for each simulation set (May to September; MJJAS). Precipitation (mm/day) is averaged over the blue-shaded monsoon domain shown in Fig. 2 and SST is averaged over 0°-50°N, 60°-130°E. All_forcings is shown in black, Orb_Ghg in green, Orb_Ice in purple and Orb in blue.
